# Burden of non-communicable diseases among adolescents and young adults aged 10–24 years in Middle East and North Africa, 1990–2023: a systematic analysis of the Global Burden of Diseases Study 2023

**DOI:** 10.1016/j.eclinm.2026.103827

**Published:** 2026-03-13

**Authors:** Dian Kusuma, Abrari Noor Hasmi, Ankita Shukla, Iffat Elbarazi, Ali H. Mokdad, Basema Saddik

**Affiliations:** a Department of Public Health and Epidemiology, College of Medicine and Health Sciences, Khalifa University of Science and Technology, Abu Dhabi, United Arab Emirates; b Department of Mathematics, College of Computing and Mathematical Sciences, Khalifa University of Science and Technology, Abu Dhabi, United Arab Emirates; c Center of Excellence for Public Health, University of Sharjah, Sharjah, United Arab Emirates; d Institute of Public Health, College of Medicine and Health Sciences, United Arab Emirates University, Al Ain, United Arab Emirates; e Institute for Health Metrics and Evaluation, University of Washington, Seattle, USA; f School of Population Health, Faculty of Medicine and Health, University of New South Wales, Sydney, Australia

**Keywords:** Adolescent health, Chronic diseases, Middle East and North Africa, Global Burden of Disease Study 2023, Mortality, Disability-adjusted life-years

## Abstract

**Background:**

Non-communicable diseases (NCDs) are a major contributor to morbidity and mortality worldwide, yet their burden among adolescents and young adults in the Middle East and North Africa (MENA) region remains underexplored. Understanding age-, sex-, and country-specific patterns is critical in a region marked by conflict, demographic transition, and rapid urbanization.

**Methods:**

We used estimates from the Global Burden of Diseases, Injuries, and Risk Factors Study (GBD) 2023 to quantify mortality and disability due to NCDs among people aged 10–24 years across 21 MENA countries from 1990 to 2023. Causes were analyzed by three hierarchical levels of the GBD 2023 cause list. For each cause, mortality, years of life lost (YLLs), years lived with disability (YLDs), and disability-adjusted life-years (DALYs) with 95% uncertainty intervals (UIs) were extracted and stratified by sex, age group, and country. Temporal trends (1990–2023) and associations between Level 2 NCD DALY rates and each country's Socio-demographic Index (SDI) were assessed using correlation. Analyses were performed using R (version 2025.09.0) and Julia (version 1.10).

**Findings:**

In 2023, NCDs accounted for 76.0% (95% UI 71.5–79.6) of all YLDs and 29.0% (27.0–32.3) of total deaths among young people aged 10–24 years in MENA. Among NCDs, cardiovascular diseases and neoplasms were the leading causes of mortality (9.64 [7.64–12.14] and 6.93 [6.05–7.78] per 100,000, respectively), whereas mental disorders were the main contributors to YLDs (2916.2 [2013.4–4165.8] per 100,000) and DALYs (2916.3 [2013.4–4165.9] per 100,000). NCD mortality was higher in males than females (37.9 vs 18.6 per 100,000), while females had slightly higher DALY rates (8970.4 vs 8221.6 per 100,000). From 1990 to 2023, mortality due to NCDs declined by 33.6% and YLLs by 34.2%, reflecting steady improvements until 2019 before plateauing during and after the COVID-19 period. Over the same timeframe, YLDs declined modestly until 2019 but increased thereafter (+8.6% [–3.4 to 21.9]), largely due to mental and metabolic disorders. The largest declines were seen for digestive (−60.9%) and cardiovascular diseases (−43.6%), whereas increases occurred in mental (128.4%) and substance-use disorders (12.9%). Countries with higher SDI scores had lower DALY rates for cardiovascular, digestive, neoplastic, and other NCDs.

**Interpretation:**

Integrated, youth-responsive NCD and mental health services are needed to address rising disability and health loss among young people in the MENA region.

**Funding:**

Khalifa University of Science and Technology.

**Research in context**

**Evidence before this study**

We conducted a systematic search of PubMed for English-language research articles published up to September 30, 2025. The search strategy included the following terms in titles or abstracts: (“adolescent” OR “young people”) AND (“disability” OR “mortality”) AND (“Middle East and North Africa” OR “MENA” OR “Eastern Mediterranean” OR “EMRO”) AND (“non-communicable disease” OR “NCD”). We identified several studies addressing health among adolescents and young adults in MENA, but most focused on obesity, nutrition, or mental health, and very few comprehensively quantified the burden of NCDs. Existing analyses were either country-specific, disease-specific, or examined broader age groups within global or adult-focused studies. A regional analysis is warranted in MENA because countries share common demographic transitions, conflict-related exposures, health-system constraints, and policy frameworks, and regional estimates can support benchmarking, coordinated strategies, and cross-country learning. Although previous Global Burden of Disease (GBD) reports have estimated NCD patterns among adults in MENA, no prior study has systematically assessed both fatal and non-fatal NCD burden among people aged 10–24 years or examined how these trends have evolved through the COVID-19 period.

**Added value of this study**

This study provides the first comprehensive and standardized analysis of the burden of non-communicable diseases (NCDs) among young people aged 10–24 years across 21 countries in the Middle East and North Africa (MENA) region from 1990 to 2023. Using data from the Global Burden of Diseases, Injuries, and Risk Factors Study (GBD) 2023, it offers the most up-to-date and comparable estimates of mortality and disability across causes, age groups, sexes, and locations. GBD 2023 incorporates newly available data and enhanced modelling methods applied consistently across the full time series. Our analysis shows a substantial decline in mortality and years of life lost (YLLs)—particularly from cardiovascular and digestive diseases—until 2019, followed by a plateau during and after the COVID-19 period. In contrast, years lived with disability (YLDs) and overall disability-adjusted life-years (DALYs) increased after 2020, driven mainly by mental and metabolic disorders. These findings reveal a growing divergence between mortality and disability trends, reflecting persistent socioeconomic and health-system inequalities across the region.

**Implications of all the available evidence**

Our findings directly support the priorities outlined in the Arab Strategy for Maternal, Child, and Adolescent Health 2019–2030 and reflect a broader transition in adolescent health in the MENA region from declining mortality to rising chronic disability, a shift accentuated by the COVID-19 pandemic. While reductions in NCD–related mortality indicate progress, the predominance of mental disorders as contributors to disability among adolescents and young adults underscores the need for youth-responsive mental health services, consistent with Sustainable Development Goal (SDG) target 3.5. Concurrently, the continued contribution of cardiovascular diseases and neoplasms to premature mortality aligns with SDG target 3.4 and highlights the importance of early risk-factor prevention and continuity of care during adolescence and young adulthood. Strengthening adolescent-responsive NCD prevention, integrating early detection within primary care, and improving regional surveillance will be essential to address the growing disability burden and ensure equitable recovery.

## Introduction

Adolescence and young adulthood (defined here as ages 10–24 years) represent a critical window for establishing health trajectories that shape morbidity and mortality across the life course. This age group comprises around 24% of the world's population representing approximately 2 billion individuals.^1^ Despite their demographic and developmental significance, adolescents and young people have historically been overlooked in health policy frameworks. Initiatives such as the Global Strategy for Women's, Children's and Adolescents' Health and the Countdown to 2030 have sought to elevate adolescent health on the agenda. However, progress remains uneven and insufficient. By 2030, more than 1 billion adolescents are projected to live in countries facing preventable and treatable health challenges, including mental health disorders, obesity, anemia, and injury.^2^ Persistent barriers such as inadequate financing, limited age- and sex-disaggregated data, and emerging threats like climate change and digital exposure, continue to constrain meaningful improvements in adolescent well-being.^3^

Although often perceived as a period of relatively good health, adolescence and young adulthood is increasingly marked by the early onset of chronic, non-communicable conditions that extend into adulthood. Non-communicable diseases (NCDs) remain the leading global cause of mortality and disability, accounting for 43.8 million deaths and 1.73 billion disability-adjusted life-years (DALYs) in 2021.^4^ Recent evidence from the Global Burden of Disease (GBD) studies and related research shows that NCDs including neoplasms, cardiovascular diseases, and mental disorders are increasingly contributing to health loss in adolescents and young adults.^5^, ^6^, ^7^ This epidemiological shift underscores the need to quantify both fatal and non-fatal health loss when designing adolescent health interventions.

The MENA region faces distinct demographic and epidemiological challenges, encompassing 21 countries. As of 2023, it is home to approximately 140 million individuals aged 10–24 years, representing about one-quarter of the regional population.^8^ This demographic concentration presents both a development opportunity and a challenge if rising adolescent morbidity remains unaddressed. Countries across MENA span diverse stages of epidemiological transition, from high-income Gulf states to low-income and conflict-affected settings. Political instability over the past decade—including the 2010–2011 Arab Spring uprisings and prolonged conflicts in Syria, Yemen, Palestine, Sudan, and parts of Iraq and Libya—has strained health systems and disrupted service delivery, with inequities further exacerbated during the COVID-19 pandemic, when unequal access to testing, vaccines, mental-health support, and essential care services intensified existing health-system inequities across the region.^9^ Although major hostilities have recently subsided in Syria, long-term health, social, and economic consequences persist, disproportionately affecting young people.^10^^,^^11^ Against this backdrop of instability, many MENA countries are undergoing rapid urbanization, dietary transitions, and rising obesity and metabolic risk factors,^12^^,^^13^ while others continue to face limited access to preventive and mental health care.^14^ These overlapping challenges have accelerated the region's shift from infectious to non-communicable diseases, which now account for approximately 2.3 million deaths and 121.5 million DALYs across all ages.^4^

Despite growing recognition of NCDs as a regional priority, adolescents and young adults have received limited focused attention, even though they represent nearly one-third of the population in several MENA countries and are already exhibiting early manifestations of chronic disease risk. To date, no comprehensive regional assessment has quantified both the fatal and non-fatal NCD burden among adolescents. While regional initiatives have targeted key behavioral and metabolic risk factors, such as tobacco use, unhealthy diet, and physical inactivity, progress in reducing premature NCD mortality and disability remains uneven.^13^^,^^14^ Understanding how these risks translate into mortality and disability among young people is therefore essential for designing prevention strategies that can alter long-term disease trajectories. The wide variation in socioeconomic development, health system capacity, and demographic structure across MENA makes it an important context for examining differences in NCD patterns and their determinants among young people.

Building on this context, we used estimates from the GBD 2023,^15^^,^^16^ to address the current evidence gap on NCD burden in the MENA region among adolescents and young adults. Specifically, we aimed to: (1) provide a comprehensive assessment of mortality and disability due to NCDs among people aged 10–24 years across 21 MENA countries, disaggregated by cause, sex, age, location, and time (1990–2023); and (2) examine associations between DALY rates—comprising years of life lost (YLLs) and years lived with disability (YLDs)—and the Socio-demographic Index (SDI), a composite measure of income per capita, educational attainment, and fertility rate. Using comparable estimates from GBD 2023, we examined patterns of mortality and disability by cause, sex, and country to understand how health trajectories among young people in MENA are evolving. By situating these trends within the region's broader demographic and developmental transitions, this analysis establishes a critical baseline for adolescent health monitoring and provides much needed evidence to inform targeted prevention strategies and policy priorities in the post-pandemic era.

## Methods

### Overview

This study adopted the broad age definition for adolescence and young adulthood (10–24 years) to capture the biological, social, and neurocognitive development characteristic of this population. We used estimates from the Global Burden of Diseases, Injuries, and Risk Factors Study (GBD) 2023,^15^^,^^16^ which provides internally consistent, comparable health metrics for 204 countries and territories, including all 21 countries in the MENA region.

GBD 2023 generated cause-specific mortality and morbidity estimates for 292 causes of death and 375 diseases and injuries, quantified in terms of mortality, years of life lost (YLLs), years lived with disability (YLDs), and disability-adjusted life-years (DALYs). YLLs, YLDs, and DALYs were computed for every age–sex–location–year combination from 1990 to 2023.^15^^,^^16^

For fatal outcomes, GBD 2023 employed the Cause of Death Ensemble model (CODEm), a framework that tests multiple model classes and covariate sets and combines them based on out-of-sample predictive validity. CODEm incorporated 55,761 data sources, including vital registration, verbal autopsy, surveillance, and cancer-registry data. Deaths recorded as unspecified were reassigned using GBD's standardized redistribution algorithms for “garbage codes.” For non-fatal outcomes, GBD 2023 used the Bayesian meta-regression models DisMod-MR 2.1 and the updated DisMod-AT, which allows the inclusion of cohort and temporal effects and age-sex covariates. Spatiotemporal Gaussian process regression (ST-GPR) was used to smooth sparse data over time and space.^15^^,^^16^

All GBD 2023 estimates follow the Guidelines for Accurate and Transparent Health Estimates Reporting (GATHER). The GBD hierarchical cause list contains four levels of mutually exclusive and collectively exhaustive causes: Level 1 (communicable, maternal, neonatal, and nutritional diseases; non-communicable diseases; injuries); Level 2 (22 aggregated cause groups); Level 3 and 4 progressively specific sub-causes. For NCDs, level 2 categories were used to summarize overall patterns, while analyses were conducted at level 3 to characterize condition-specific mortality and disability, with results presented at both levels where relevant.

### Data analysis

Analyses were conducted for 21 MENA countries (Algeria, Bahrain, Egypt, Iran, Iraq, Jordan, Kuwait, Lebanon, Libya, Morocco, Oman, Palestine, Qatar, Saudi Arabia, Sudan, Syria, Tunisia, Türkiye, United Arab Emirates, Yemen, and Afghanistan) from 1990 to 2023, stratified by sex (male, female, both) and age group (10–14, 15–19, 20–24 years).

We reported mortality, years of life lost (YLLs), years lived with disability (YLDs), and disability-adjusted life-years (DALYs) as rates per 100,000 population. DALYs were calculated as the sum of YLLs and YLDs, where YLLs were derived by multiplying the number of deaths at each age by the standard life expectancy for that age, and YLDs were obtained by multiplying disease prevalence by the corresponding disability weight for each sequela, reflecting the severity of health loss. YLDs were further adjusted for comorbidity using a multiplicative function of disability weights to account for overlapping conditions. All estimates are presented as point values with 95% uncertainty intervals (UIs), defined as the 2.5th and 97.5th percentiles from 250 posterior draws. Differences between estimates were considered statistically significant when the 95% UIs did not overlap.^15^^,^^16^

Temporal trends were assessed by calculating the percentage change in age-standardized rates for mortality, YLLs, YLDs, and DALYs between 1990 and 2023. To explore contextual heterogeneity, we examined the association between Level 2 NCD DALY rates and each country's Socio-demographic Index (SDI) using Spearman's rank correlation. SDI is a composite of lag-distributed income per capita, mean years of schooling, and fertility rate among females younger than 25, scaled 0 (low) to 1 (high). All analyses were performed using R (version 2025.09.0 + 387) and Julia (version 1.10).

### Role of the funding source

The funder of the study had no role in study design, data collection, analysis, data interpretation, or writing of the report.

## Results

**Fig. 1:**
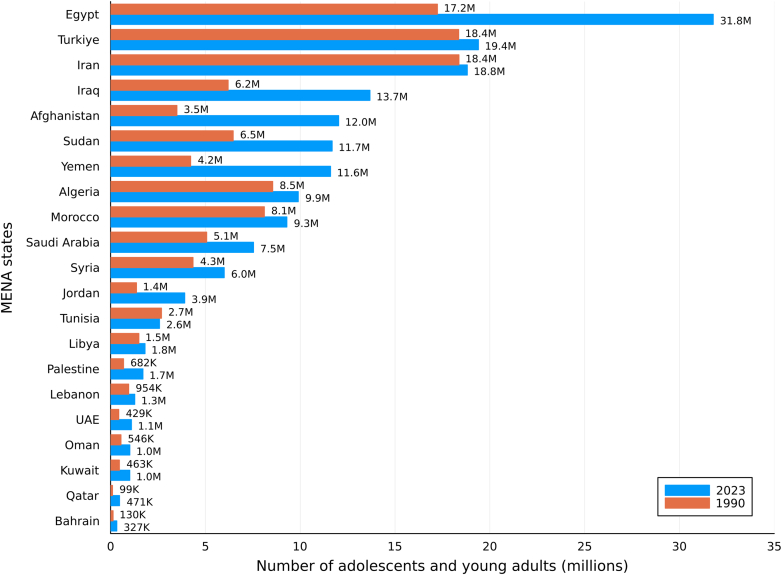
**Population shifts in young people aged 10–24 years in the MENA region by country, 1990–2023**. Note: Absolute change in population number from 1990 to 2023.

In 2023, about 197 million adolescents and young people aged 10–24 years lived in the MENA region, an increase from 145 million in 1990, representing a net growth of 52 million over the three decades. The largest populations of young people in 2023 were observed in Egypt (31.8 million), Türkiye (19.4 million), Iran (18.8 million), and Iraq (13.7 million), which together accounted for nearly half of the region's youth (Fig. 1). The greatest absolute population increases between 1990 and 2023 occurred in Egypt (+14.6 million), Türkiye (+13.4 million), and Iraq (+7.5 million), while more gradual growth was noted in Algeria, Morocco, and Sudan. In contrast, Iran and several Gulf Cooperation Council (GCC) countries experienced modest adolescent population growth, reflecting lower fertility and earlier stages of demographic transition.

### Mortality

In 2023, the overall all-cause mortality rate among young people aged 10–24 years in the MENA region was 94.6 (95% UI 90.5–97.9) deaths per 100,000 population (Appendix Figs. S1A and 2). Between 1990 and 2023, mortality trends varied by cause category. When disaggregated by Level 1 causes, injury-related mortality increased sharply between 2010 and 2015, coinciding with periods of the Arab Spring and regional conflicts, while mortality from NCDs and communicable, maternal, neonatal and nutritional (CMNN) causes declined steadily throughout this period. Decade-specific estimates (1990, 2000, 2010, 2023) and percentage changes are presented in Appendix Table S8A, and trends are displayed in Appendix Fig. S1B.

In 2023, NCDs accounted for 29.0% (27.0–32.3) of all deaths among young people (Appendix Table S2). At Level 2, the leading contributors were cardiovascular diseases (9.64 [7.64–12.14] per 100,000 population), neoplasms (6.93 [6.05–7.78] per 100,000 population), and other NCDs (4.70 [3.87–5.70] per 100,000 population), representing 76.5% of all NCD mortality. At Level 3, ischemic heart disease was the leading NCD-specific cause of death at 3.54 [2.63–4.68] per 100,000 population.

Across all Level 1 causes, NCDs ranked as the second leading cause of death for both males and females aged 10–24 years. Among females, NCDs accounted for 37.0% (34.1–40.5) of total deaths, with increasing proportions by age groups: 38.0% (10–14 years), 37.0% (15–19 years), and 47.0% (20–24 years). Among males, NCD-related deaths comprised 25.0% (22.0–29.4) of total deaths with proportions of 32.0%, 24.0%, and 23.0% across the three respective age categories (Appendix Table S3). Absolute mortality rates increased with age in both sexes—from 20.6 (18.8–23.6) per 100,000 in adolescents and young adults aged 10–14 years, to 25.1 (21.8–28.9) in those aged 15–19 years, and 30.2 (26.8–34.0) among young adults aged 20–24 years (Appendix Table S3). Patterns of NCD mortality by age and sex are illustrated in Appendix Fig. S3.

**Fig. 2:**
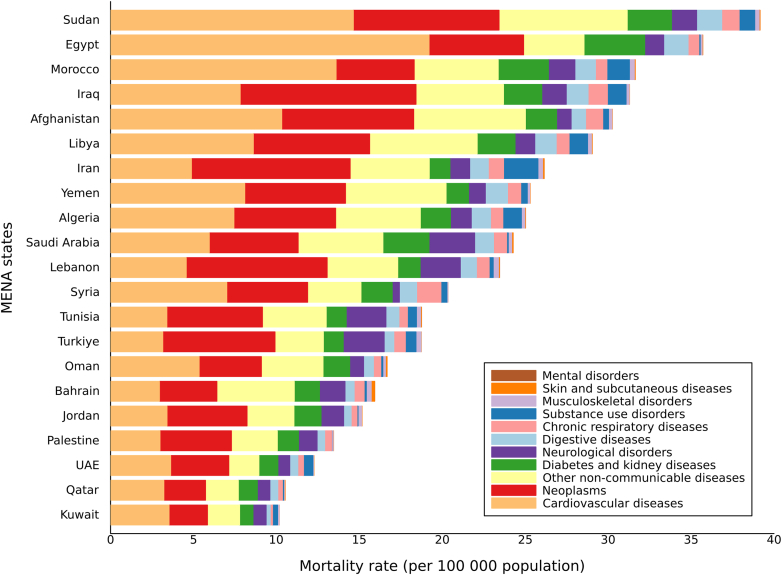
**Mortality rate per 100,000 population due to level 2 non-communicable diseases in 10–24 years in both sexes, MENA region by country, 2023**. Note: • This aggregate cause contains the following level 3 causes: congenital birth defects; urinary diseases; gynaecological diseases; haemoglobinopathies and haemolytic anaemias; endocrine, metabolic, blood, and immune disorders; and oral disorders. • Other non-communicable diseases include Congenital birth defects; Urinary diseases and male infertility; Gynecological diseases; Hemoglobinopathies and hemolytic anemias; Endocrine, metabolic, blood, and immune disorders; Oral disorders. • Mortality rate due to Level 1 NCD in 10–24 years in MENA for 2023 was 27.8 (95% UI: 25.3–30.8). • Mortality attributable to mental disorders among adolescents is relatively small compared with others; as a result, the corresponding segment may appear narrow in the stacked bars.

Substantial variation in NCD mortality was observed across countries (Fig. 2). In 2023, the highest Level 2 NCD mortality rates were recorded in Sudan and Egypt, each reporting rates more than three times higher than those in Kuwait, Qatar, and the United Arab Emirates. Six countries—Sudan, Egypt, Morocco, Iraq, Afghanistan, and Libya—had mortality levels significantly higher than the regional composite estimate (27.8 [25.3–30.8] per 100,000). Cardiovascular diseases were the leading Level 2 cause of NCD mortality in 13 countries, neoplasms in 7 countries (Iraq, Iran, Lebanon, Tunisia, Türkiye, Jordan, Palestine), and mental disorders in 1 country (Bahrain).

Between 1990 and 2023, the NCD mortality rate among young people declined from 41.9 (36.4–47.3) to 27.8 (25.3–30.8) per 100,000 population, a reduction of 33.6% (95% UI –22.1 to −43.7). However, after a steady decline to (27.2 [25.2–29.6]) in 2019, mortality rates plateaued during and after the COVID-19 period, reaching (27.1 [25.1–29.5] in 2020 and 27.8 [25.3–30.8] in 2023) (Table 1; Appendix Fig. S4). At Level 2, declines were the greatest for digestive diseases (−60.9% [–72.8 to −39.5]) and cardiovascular diseases (−43.6% [–60.1 to −20.6]), while increases were observed for mental disorders (128.4% [–46.2 to 1356.4])—entirely attributable to eating disorders—and substance use disorders (12.9% [–34.0 to 86.7]) (Table 1). Mental disorders include depressive disorders, anxiety disorders, bipolar disorder, schizophrenia, eating disorders, autism spectrum disorders, attention-deficit/hyperactivity disorder, conduct disorder, intellectual disability, and other mental disorders (Table 1). Although mental disorders contribute minimally to mortality—limited primarily to eating disorders at level 3—they are the leading contributors to disability and overall DALYs among adolescents and young adults (Table 1).

**Table 1:** Mortality and DALY rate per 100,000 population in adolescents and young adults aged 10–24 years in MENA countries in 1990 and 2023, and percentage change from 1990 to 2023.

	Mortality rate per 100,000 population			DALY rate per 100,000 population		
	1990	2023	Percentage change, 1990–2023	1990	2023	Percentage change, 1990–2023
All non-communicable diseases	41.86 (36.42–47.29)	27.81 (25.33–30.75)	−33.55% (−43.71 to −22.10)	9128.65 (7381.00–10894.37)	8607.79 (6747.88–10602.02)	−5.71% (−14.30 to 4.15)
**Neoplasms**	**8.74 (7.54–9.92)**	**6.93 (6.05–7.78)**	**−20.63% (−32.83 to −4.57)**	**644.51 (556.37–731.96)**	**510.72 (446.87–574.20)**	**−20.76% (−32.83 to −4.91)**
Esophageal cancer	0.01 (0.01–0.02)	0.01 (0.01–0.02)	−12.24% (−43.43 to 30.47)	0.90 (0.61–1.20)	0.79 (0.59–1.04)	−11.97% (−43.33 to 30.83)
Stomach cancer	0.22 (0.15–0.31)	0.13 (0.09–0.16)	−41.62% (−61.29 to −9.91)	15.74 (10.38–21.87)	9.19 (6.18–11.64)	−41.60% (−61.40 to −9.88)
Liver cancer	0.22 (0.14–0.31)	0.21 (0.14–0.29)	−6.58% (−40.54 to 57.05)	16.24 (10.34–22.19)	14.98 (10.08–21.08)	−7.76% (−41.06 to 54.67)
Larynx cancer	0.02 (0.01–0.02)	0.02 (0.01–0.02)	0.32% (−31.89 to 41.56)	1.19 (0.80–1.68)	1.19 (0.88–1.57)	0.54% (−31.69 to 41.96)
Tracheal, bronchus, and lung cancer	0.20 (0.14–0.27)	0.26 (0.19–0.34)	30.32% (−12.56 to 100.76)	14.00 (10.11–18.87)	18.13 (13.47–23.86)	29.48% (−13.09 to 99.61)
Breast cancer	0.06 (0.04–0.08)	0.11 (0.08–0.15)	80.07% (11.39–197.78)	4.43 (3.17–5.96)	8.16 (5.56–11.20)	84.22% (12.75–204.87)
Cervical cancer	0.07 (0.03–0.11)	0.05 (0.03–0.07)	−31.08% (−64.25 to 45.35)	4.85 (2.49–8.13)	3.37 (1.96–5.40)	−30.51% (−63.78 to 46.16)
Uterine cancer	0.01 (0.00–0.01)	0.01 (0.01–0.01)	36.96% (−23.14 to 140.12)	0.40 (0.25–0.59)	0.57 (0.36–0.86)	39.69% (−20.96 to 144.03)
Prostate cancer	0.00 (0.00–0.01)	0.01 (0.00–0.01)	32.26% (−25.00 to 116.03)	0.30 (0.20–0.45)	0.42 (0.27–0.63)	39.32% (−21.12 to 127.53)
Colon and rectum cancer	0.15 (0.11–0.21)	0.19 (0.14–0.25)	27.85% (−12.47 to 90.54)	10.71 (7.96–14.94)	13.84 (10.04–17.93)	29.13% (−12.08 to 92.33)
Lip and oral cavity cancer	0.02 (0.02–0.03)	0.03 (0.02–0.03)	11.91% (−26.38 to 59.84)	1.69 (1.24–2.31)	1.90 (1.41–2.48)	12.53% (−26.04 to 60.89)
Nasopharynx cancer	0.09 (0.06–0.12)	0.05 (0.03–0.06)	−46.31% (−70.67 to −15.64)	6.42 (4.26–9.09)	3.53 (2.39–4.95)	−44.95% (−69.68 to −14.50)
Other pharynx cancer	0.00 (0.00–0.01)	0.01 (0.00–0.01)	35.06% (−9.39 to 105.99)	0.28 (0.19–0.38)	0.38 (0.27–0.53)	35.15% (−9.30 to 106.04)
Gallbladder and biliary tract cancer	0.01 (0.01–0.01)	0.01 (0.01–0.01)	3.37% (−29.93 to 36.98)	0.59 (0.43–0.74)	0.61 (0.45–0.79)	3.82% (−29.45 to 37.74)
Pancreatic cancer	0.02 (0.01–0.02)	0.03 (0.02–0.04)	74.84% (21.25–164.54)	1.31 (0.93–1.69)	2.28 (1.71–2.98)	73.96% (20.75–163.40)
Malignant skin melanoma	0.01 (0.01–0.02)	0.01 (0.01–0.02)	−0.09% (−37.25 to 39.64)	0.99 (0.55–1.42)	1.03 (0.66–1.42)	3.90% (−35.00 to 43.29)
Non-melanoma skin cancer	0.01 (0.00–0.01)	0.01 (0.01–0.02)	72.73% (10.00–188.04)	0.42 (0.29–0.61)	0.72 (0.49–1.08)	72.19% (9.79–186.56)
Ovarian cancer	0.04 (0.03–0.07)	0.07 (0.05–0.10)	63.42% (−10.83 to 181.49)	3.16 (2.12–4.81)	5.19 (3.53–7.42)	64.21% (−10.00 to 183.71)
Testicular cancer	0.05 (0.03–0.08)	0.05 (0.04–0.08)	3.12% (−45.75 to 105.31)	3.73 (2.21–5.81)	4.16 (2.71–6.14)	11.72% (−42.72 to 122.28)
Kidney cancer	0.06 (0.04–0.08)	0.06 (0.04–0.08)	−2.08% (−36.11 to 39.37)	4.54 (3.08–6.10)	4.47 (3.12–5.89)	−1.50% (−35.73 to 39.57)
Bladder cancer	0.03 (0.02–0.04)	0.02 (0.01–0.03)	−25.55% (−51.80 to 36.15)	2.13 (1.36–3.15)	1.64 (1.13–2.26)	−22.80% (−49.59 to 39.59)
Brain and central nervous system cancer	1.27 (0.89–1.69)	1.21 (0.90–1.53)	−5.08% (−25.74 to 25.29)	94.81 (66.13–125.85)	89.53 (66.88–113.72)	−5.57% (−26.32 to 24.56)
Thyroid cancer	0.03 (0.02–0.04)	0.03 (0.02–0.04)	−7.34% (−37.96 to 39.56)	2.46 (1.79–3.44)	2.41 (1.82–3.24)	−2.10% (−35.30 to 50.10)
Mesothelioma	0.01 (0.00–0.01)	0.01 (0.00–0.01)	20.31% (−24.51 to 98.49)	0.41 (0.27–0.62)	0.49 (0.32–0.67)	20.16% (−24.58 to 98.23)
Hodgkin lymphoma	0.29 (0.16–0.43)	0.15 (0.08–0.22)	−49.38% (−67.82 to −17.44)	21.33 (12.01–31.68)	11.04 (5.80–16.46)	−48.22% (−67.22 to −15.21)
Non-Hodgkin lymphoma	0.58 (0.43–0.76)	0.50 (0.38–0.66)	−13.23% (−43.06 to 30.97)	42.48 (31.36–55.39)	37.14 (28.02–48.62)	−12.56% (−42.33 to 31.90)
Multiple myeloma	0.01 (0.01–0.01)	0.02 (0.01–0.02)	69.51% (2.15–184.72)	0.64 (0.42–0.91)	1.10 (0.77–1.47)	70.43% (2.42–185.92)
Leukemia	3.25 (2.51–4.18)	2.24 (1.81–2.74)	−31.05% (−49.94 to −9.52)	240.82 (185.50–310.12)	165.05 (132.39–201.42)	−31.46% (−50.15 to −10.00)
Eye cancer	0.00 (0.00–0.00)	0.00 (0.00–0.00)	−8.82% (−46.61 to 32.36)	0.18 (0.11–0.30)	0.17 (0.11–0.28)	−7.36% (−43.43 to 32.98)
Soft tissue and other extraosseous sarcomas	0.16 (0.10–0.27)	0.12 (0.08–0.18)	−24.88% (−58.79 to 18.03)	11.91 (7.38–19.82)	8.92 (5.75–13.59)	−25.04% (−58.64 to 17.94)
Malignant neoplasm of bone and articular cartilage	0.81 (0.53–1.33)	0.66 (0.48–0.94)	−19.23% (−48.48 to 28.49)	60.27 (39.21–98.52)	48.57 (35.82–69.30)	−19.41% (−48.24 to 27.76)
Neuroblastoma and other peripheral nervous cell tumors	0.02 (0.01–0.03)	0.02 (0.02–0.04)	21.71% (−20.66 to 98.96)	1.47 (0.99–2.23)	1.79 (1.22–2.66)	21.94% (−20.89 to 99.91)
Other malignant neoplasms	0.86 (0.65–1.14)	0.54 (0.41–0.69)	−37.32% (−58.57 to −6.07)	64.52 (48.87–85.17)	40.32 (30.41–51.43)	−37.51% (−58.74 to −6.62)
Other neoplasms	0.12 (0.07–0.19)	0.09 (0.06–0.14)	−22.85% (−53.22 to 16.83)	9.21 (5.29–14.10)	7.63 (4.84–11.49)	−17.19% (−49.52 to 22.58)
**Cardiovascular diseases**	**17.10 (12.88–21.48)**	**9.64 (7.64–12.14)**	**−43.60% (−60.11 to −20.56)**	**1357.22 (1048.08–1661.94)**	**802.37 (654.88–983.16)**	**−40.88% (−56.56 to −19.91)**
Rheumatic heart disease	2.48 (1.40–3.50)	0.74 (0.43–1.19)	−70.29% (−84.73 to −45.37)	205.29 (128.09–280.92)	77.55 (54.30–111.93)	−62.22% (−77.39 to −39.21)
Ischemic heart disease	4.82 (3.38–6.71)	3.52 (2.63–4.68)	−26.95% (−54.23 to 11.53)	347.29 (243.75–478.89)	253.28 (191.57–333.32)	−27.07% (−53.85 to 10.10)
Stroke	6.58 (4.55–8.99)	3.07 (2.13–4.25)	−53.39% (−70.27 to −21.56)	545.27 (401.38–719.98)	274.16 (209.27–359.13)	−49.72% (−66.12 to −22.61)
Hypertensive heart disease	0.41 (0.23–0.66)	0.33 (0.22–0.49)	−19.29% (−55.02 to 43.94)	30.10 (17.06–47.57)	24.47 (16.57–35.53)	−18.71% (−54.01 to 41.69)
Cardiomyopathy and myocarditis	0.69 (0.38–1.16)	0.46 (0.28–0.78)	−33.81% (−66.58 to 27.14)	54.16 (30.42–88.15)	37.02 (23.68–60.57)	−31.64% (−64.77 to 26.11)
Pulmonary Arterial Hypertension	0.19 (0.09–0.33)	0.08 (0.05–0.14)	−56.40% (−80.41 to −13.35)	13.87 (6.83–24.52)	6.00 (3.32–10.28)	−56.72% (−80.40 to −14.19)
Atrial fibrillation and flutter	–	–	–	0.00 (0.00–0.00)	0.00 (0.00–0.00)	–
Aortic aneurysm	0.02 (0.01–0.03)	0.03 (0.02–0.05)	75.61% (−12.04 to 267.97)	1.37 (0.81–2.15)	2.38 (1.58–3.46)	74.39% (−12.63 to 265.30)
Lower extremity peripheral arterial disease	–	–	–	0.00 (0.00–0.00)	0.00 (0.00–0.00)	–
Endocarditis	0.15 (0.10–0.25)	0.10 (0.07–0.15)	−36.35% (−63.58 to 13.54)	11.35 (7.25–18.17)	7.19 (4.91–11.00)	−36.61% (−63.32 to 12.30)
Non-rheumatic valvular heart disease	0.21 (0.12–0.33)	0.21 (0.13–0.32)	−2.28% (−42.66 to 71.30)	15.01 (8.16–23.57)	14.61 (8.88–22.21)	−2.68% (−42.69 to 70.30)
Other cardiovascular and circulatory diseases	1.53 (0.90–2.36)	1.10 (0.76–1.50)	−28.14% (−58.02 to 23.72)	133.53 (82.91–197.07)	105.70 (77.28–138.81)	−20.84% (−51.75 to 24.84)
**Chronic respiratory diseases**	**1.31 (0.77–1.90)**	**0.82 (0.61–1.10)**	**−37.06% (−63.06 to 14.76)**	**324.32 (230.91–439.20)**	**298.33 (212.89–414.89)**	**−8.01% (−21.41 to 6.92)**
Chronic obstructive pulmonary disease	0.41 (0.20–0.65)	0.30 (0.20–0.41)	−26.58% (−60.45 to 45.28)	43.60 (28.87–61.65)	35.71 (28.00–44.75)	−18.10% (−43.39 to 25.08)
Pneumoconiosis	0.01 (0.00–0.02)	0.01 (0.00–0.02)	2.29% (−67.73 to 189.13)	0.71 (0.33–1.34)	0.72 (0.42–1.29)	1.52% (−60.88 to 141.23)
Asthma	0.65 (0.36–1.02)	0.29 (0.19–0.43)	−55.84% (−75.12 to −15.28)	257.25 (172.84–374.00)	238.48 (153.84–355.09)	−7.30% (−19.41 to 4.36)
Interstitial lung disease and pulmonary sarcoidosis	0.04 (0.02–0.08)	0.05 (0.03–0.08)	23.58% (−57.44 to 159.80)	3.12 (1.51–6.23)	3.82 (2.34–6.16)	22.29% (−55.72 to 145.88)
Other chronic respiratory diseases	0.20 (0.11–0.36)	0.18 (0.11–0.27)	−12.24% (−56.79 to 91.88)	19.63 (12.23–31.74)	19.61 (14.60–26.02)	−0.14% (−40.04 to 76.31)
**Digestive diseases**	**2.89 (2.19–3.63)**	**1.13 (0.87–1.43)**	**−60.87% (−72.78 to −39.54)**	**308.40 (248.29–385.99)**	**172.44 (137.78–215.05)**	**−44.08% (−55.02 to −26.18)**
Cirrhosis and other chronic liver diseases	2.00 (1.47–2.57)	0.75 (0.57–0.96)	−62.47% (−75.70 to −43.69)	158.65 (119.48–200.21)	62.43 (48.41–77.16)	−60.65% (−73.37 to −43.46)
Appendicitis	0.13 (0.05–0.21)	0.04 (0.02–0.06)	−68.10% (−84.61 to −24.77)	13.15 (7.32–20.21)	6.68 (4.50–9.48)	−49.22% (−67.56 to −13.67)
Paralytic ileus and intestinal obstruction	0.15 (0.10–0.23)	0.08 (0.05–0.13)	−46.35% (−72.27 to 6.97)	11.57 (7.30–17.13)	6.35 (4.34–9.85)	−45.07% (−70.67 to 6.58)
Inguinal, femoral, and abdominal hernia	0.01 (0.00–0.02)	0.00 (0.00–0.01)	−62.03% (−83.97 to 17.76)	13.59 (7.77–23.78)	11.49 (6.38–18.86)	−15.47% (−30.82 to 4.64)
Inflammatory bowel disease	0.05 (0.03–0.09)	0.04 (0.02–0.06)	−26.81% (−63.90 to 61.14)	5.15 (3.37–7.85)	4.30 (2.98–6.00)	−16.46% (−48.61 to 46.84)
Vascular intestinal disorders	0.02 (0.01–0.02)	0.01 (0.01–0.02)	−27.60% (−66.29 to 48.65)	1.20 (0.71–1.88)	0.92 (0.59–1.42)	−23.67% (−59.52 to 43.54)
Gallbladder and biliary diseases	0.03 (0.02–0.05)	0.02 (0.01–0.04)	−14.35% (−60.56 to 60.21)	17.63 (11.00–26.10)	16.58 (10.16–24.90)	−5.93% (−16.93 to 5.59)
Pancreatitis	0.04 (0.02–0.08)	0.03 (0.02–0.04)	−37.83% (−67.31 to 23.24)	4.50 (2.75–7.01)	3.27 (2.23–4.67)	−27.42% (−52.26 to 12.44)
Upper digestive system diseases	0.38 (0.25–0.60)	0.09 (0.06–0.16)	−75.92% (−87.32 to −57.27)	75.79 (50.82–113.04)	54.62 (32.03–88.98)	−27.93% (−42.92 to −14.22)
Other digestive diseases	0.07 (0.04–0.11)	0.06 (0.03–0.09)	−18.67% (−58.12 to 52.83)	7.17 (4.69–10.16)	5.80 (4.15–8.31)	−19.05% (−47.82 to 26.42)
**Neurological disorders**	**1.54 (0.98–2.08)**	**1.43 (1.16–1.76)**	**−7.08% (−34.10 to 46.34)**	**897.85 (636.97–1223.37)**	**890.91 (627.55–1213.95)**	**−0.77% (−8.69 to 7.88)**
Alzheimer's disease and other dementias	–	–	–	0.00 (0.00–0.00)	0.00 (0.00–0.00)	–
Parkinson's disease	0.00 (0.00–0.00)	0.00 (0.00–0.00)	25.38% (−10.87 to 64.19)	0.05 (0.04–0.06)	0.06 (0.05–0.09)	35.48% (2.68–70.88)
Idiopathic epilepsy	1.43 (0.86–1.96)	0.97 (0.73–1.26)	−32.07% (−52.69 to 11.83)	226.64 (160.65–316.99)	166.49 (111.40–244.74)	−26.54% (−47.08 to 2.83)
Multiple sclerosis	0.02 (0.01–0.04)	0.02 (0.01–0.04)	33.76% (−47.74 to 285.26)	3.72 (2.47–5.70)	4.47 (3.16–6.47)	20.07% (−15.63 to 77.59)
Motor neuron disease	0.01 (0.01–0.02)	0.04 (0.02–0.08)	288.87% (56.38–793.34)	1.36 (0.88–2.35)	3.61 (2.30–6.39)	165.08% (36.69–363.25)
Headache disorders	–	–	–	650.59 (430.76–930.91)	669.12 (446.20–953.91)	2.85% (0.33–6.57)
Other neurological disorders	0.09 (0.05–0.15)	0.40 (0.30–0.53)	353.51% (134.57–797.66)	15.48 (10.37–22.97)	47.15 (35.48–63.49)	204.56% (107.76–350.32)
**Mental disorders**^a^	**0.00 (0.00–0.00)**	**0.00 (0.00–0.00)**	**128.41% (−46.22 to 1356.44)**	**2382.55 (1598.89–3295.83)**	**2916.30 (2013.43–4165.94)**	**22.40% (−7.04 to 52.48)**
Schizophrenia	–	–	–	82.48 (48.73–121.04)	87.15 (52.44–130.34)	5.65% (−1.60 to 13.91)
Depressive disorders	–	–	–	652.71 (427.63–1006.04)	944.68 (614.61–1356.17)	44.73% (4.97–95.12)
Bipolar disorder	–	–	–	118.35 (63.62–181.40)	121.49 (64.02–188.49)	2.66% (−1.70 to 6.98)
Anxiety disorders	–	–	–	965.79 (545.43–1488.83)	1171.07 (650.22–1951.61)	21.25% (−29.95 to 98.70)
Eating disorders	0.00 (0.00–0.00)	0.00 (0.00–0.00)	128.41% (−46.22 to 1356.44)	72.87 (41.19–113.90)	84.36 (47.14–134.11)	15.77% (10.54–21.83)
Autism spectrum disorders	–	–	–	120.36 (55.85–240.50)	152.67 (62.59–330.43)	26.84% (−58.66 to 234.39)
Attention-deficit/hyperactivity disorder	–	–	–	115.64 (68.44–175.14)	110.03 (65.19–166.64)	−4.85% (−9.62 to 1.67)
Conduct disorder	–	–	–	177.75 (100.44–298.53)	169.34 (96.10–281.70)	−4.73% (−7.37 to −1.50)
Idiopathic developmental intellectual disability	–	–	–	38.89 (16.04–79.06)	35.56 (12.48–72.57)	−8.59% (−42.51 to 29.20)
Other mental disorders	–	–	–	37.70 (20.54–58.94)	39.95 (22.31–63.07)	5.98% (0.16–12.81)
**Substance use disorders**	**0.64 (0.45–0.89)**	**0.72 (0.52–1.04)**	**12.89% (−34.01 to 86.73)**	**169.69 (120.71–221.29)**	**190.01 (142.53–239.66)**	**11.97% (−4.67 to 28.03)**
Alcohol use disorders	0.08 (0.05–0.12)	0.05 (0.03–0.07)	−44.65% (−69.06 to −0.37)	31.72 (18.55–50.90)	28.30 (16.30–46.97)	−10.77% (−20.76 to 0.43)
Drug use disorders	0.56 (0.37–0.81)	0.68 (0.47–0.99)	21.62% (−30.97 to 110.66)	137.97 (98.89–179.97)	161.71 (118.60–207.86)	17.20% (−0.91 to 36.86)
**Diabetes and kidney diseases**	**2.50 (1.91–3.25)**	**2.18 (1.70–2.68)**	**−12.67% (−41.03 to 17.62)**	**229.31 (181.59–285.62)**	**250.93 (202.38–301.37)**	**9.43% (−13.55 to 38.66)**
Diabetes mellitus	0.62 (0.44–0.88)	0.50 (0.39–0.65)	−18.20% (−41.57 to 23.88)	81.61 (60.82–108.32)	119.05 (89.53–162.46)	45.87% (8.78–78.13)
Acute glomerulonephritis	0.02 (0.01–0.03)	0.01 (0.01–0.02)	−37.34% (−75.10 to 75.86)	1.16 (0.46–2.22)	0.73 (0.42–1.15)	−36.91% (−74.33 to 71.12)
Chronic kidney disease	1.87 (1.32–2.60)	1.67 (1.24–2.12)	−10.64% (−44.70 to 29.27)	146.55 (106.50–199.60)	131.15 (100.91–162.09)	−10.51% (−42.49 to 25.57)
**Skin and subcutaneous diseases**	**0.04 (0.02–0.07)**	**0.04 (0.02–0.06)**	**−9.45% (−55.64 to 77.91)**	**620.42 (374.57–941.29)**	**632.62 (389.80–965.49)**	**1.97% (−1.53 to 6.41)**
Dermatitis	–	–	–	408.08 (218.27–701.50)	403.23 (217.81–684.44)	−1.19% (−2.73 to 0.07)
Psoriasis	–	–	–	14.13 (9.69–18.74)	18.67 (13.18–25.04)	32.10% (19.73–47.78)
Bacterial skin diseases	0.01 (0.00–0.02)	0.01 (0.00–0.02)	63.04% (−53.74 to 2165.77)	1.87 (1.00–3.23)	2.13 (1.30–3.62)	14.03% (−25.35 to 76.74)
Scabies	–	–	–	10.04 (5.65–16.77)	9.84 (5.64–16.55)	−2.00% (−6.63 to 2.74)
Fungal skin diseases	–	–	–	44.11 (16.67–101.70)	24.93 (9.41–56.14)	−43.49% (−49.46 to −36.79)
Viral skin diseases	–	–	–	38.11 (25.06–58.00)	37.84 (24.85–57.11)	−0.71% (−3.71 to 2.28)
Acne vulgaris	–	–	–	32.28 (19.47–54.87)	64.80 (39.02–106.94)	100.73% (85.82–118.26)
Alopecia areata	–	–	–	0.93 (0.59–1.30)	0.96 (0.60–1.39)	2.91% (−10.87 to 18.70)
Pruritus	–	–	–	0.82 (0.36–1.63)	0.89 (0.39–1.79)	8.24% (−0.59 to 17.76)
Urticaria	–	–	–	58.08 (36.08–86.04)	57.48 (36.21–85.41)	−1.03% (−4.83 to 2.84)
Decubitus ulcer	0.03 (0.01–0.05)	0.02 (0.01–0.04)	−22.13% (−62.79 to 61.03)	2.12 (1.10–3.40)	1.66 (0.95–2.69)	−21.65% (−59.63 to 54.48)
Other skin and subcutaneous diseases	0.01 (0.00–0.02)	0.01 (0.00–0.02)	−14.94% (−63.55 to 82.18)	9.86 (5.13–17.38)	10.21 (5.37–18.57)	3.53% (−4.29 to 9.62)
**Sense organ diseases**	**–**	**–**	**–**	**261.12 (173.72–359.17)**	**237.57 (157.04–329.29)**	**−9.02% (−12.19 to −6.16)**
Blindness and vision loss	–	–	–	68.88 (47.87–99.32)	54.16 (36.95–79.77)	−21.36% (−27.38 to −15.46)
Age-related and other hearing loss	–	–	–	183.35 (112.21–265.25)	174.31 (104.81–252.13)	−4.93% (−8.17 to −1.57)
Other sense organ diseases	–	–	–	8.89 (4.90–14.40)	9.10 (4.96–14.52)	2.38% (−3.94 to 9.72)
**Musculoskeletal disorders**	**0.24 (0.15–0.36)**	**0.21 (0.14–0.28)**	**−15.68% (−45.77 to 38.59)**	**777.98 (527.68–1066.52)**	**798.07 (541.39–1088.26)**	**2.58% (−0.82 to 5.74)**
Rheumatoid arthritis	0.01 (0.00–0.02)	0.01 (0.00–0.01)	−42.56% (−72.74 to 33.89)	3.92 (2.37–5.63)	5.04 (3.08–7.41)	28.56% (2.77–56.87)
Osteoarthritis	–	–	–	0.00 (0.00–0.00)	0.00 (0.00–0.00)	–
Low back pain	–	–	–	588.15 (401.00–823.28)	578.98 (393.97–824.97)	−1.56% (−4.91 to 1.80)
Neck pain	–	–	–	124.97 (67.13–207.54)	123.81 (66.21–205.36)	−0.93% (−4.40 to 2.30)
Gout	–	–	–	0.29 (0.13–0.52)	0.34 (0.16–0.61)	18.60% (12.98–27.00)
Other musculoskeletal disorders	0.23 (0.14–0.35)	0.20 (0.13–0.27)	−14.45% (−45.20 to 41.80)	60.66 (38.46–88.15)	89.90 (56.11–127.35)	48.21% (29.06–72.73)
**Other non-communicable diseases**	**6.86 (5.58–8.46)**	**4.70 (3.87–5.70)**	**−31.48% (−47.84 to −9.57)**	**1155.28 (922.03–1436.71)**	**907.53 (722.29–1128.77)**	**−21.45% (−31.60 to −9.64)**
Urinary diseases and male infertility	0.15 (0.12–0.19)	0.10 (0.08–0.12)	−32.46% (−50.22 to −8.50)	18.52 (14.17–25.47)	15.72 (11.66–23.14)	−15.11% (−31.76 to 4.57)
Gynecological diseases	0.02 (0.01–0.03)	0.01 (0.01–0.02)	−30.80% (−75.49 to 77.68)	199.12 (131.21–301.84)	202.26 (134.15–308.47)	1.58% (−2.25 to 5.44)
Hemoglobinopathies and hemolytic anemias	1.46 (1.14–1.89)	0.73 (0.53–0.94)	−50.28% (−65.93 to −20.52)	298.41 (216.36–422.39)	167.03 (120.85–219.07)	−44.03% (−56.36 to −24.23)
Endocrine, metabolic, blood, and immune disorders	0.49 (0.32–0.69)	0.62 (0.43–0.85)	28.66% (−18.88 to 108.47)	82.05 (56.98–125.39)	87.46 (63.46–123.96)	6.60% (−15.19 to 38.12)
Congenital birth defects	4.75 (3.50–6.28)	3.24 (2.44–4.17)	−31.81% (−53.49 to −1.11)	464.72 (353.64–585.63)	344.01 (277.99–415.83)	−25.98% (−44.24 to −3.01)
Oral disorders	–	–	–	92.45 (52.38–151.07)	91.04 (51.55–151.58)	−1.53% (−7.62 to 6.13)

a Note that deaths were only attributed to eating disorders.

### Years of life lost (YLLs)

In 2023, the all-cause YLL rate among people aged 10–24 years in the MENA region was 6778.5 (95% UI 6490.2–7017.8) per 100,000 population (Appendix Fig. S5). Within NCDs, the leading Level 2 contributors to YLLs were cardiovascular diseases (687.2 [544.4–864.5]) and neoplasms (500.9 [436.5–562.2]) per 100,000 population. At Level 3, ischemic heart disease (245.8 [183.4–326.5]), congenital birth defects (241.2 [182.0–310.2]), and stroke (218.9 [152.0–303.8]) per 100,000 population were the top specific causes of premature mortality (Appendix Table S4).

**Fig. 3:**
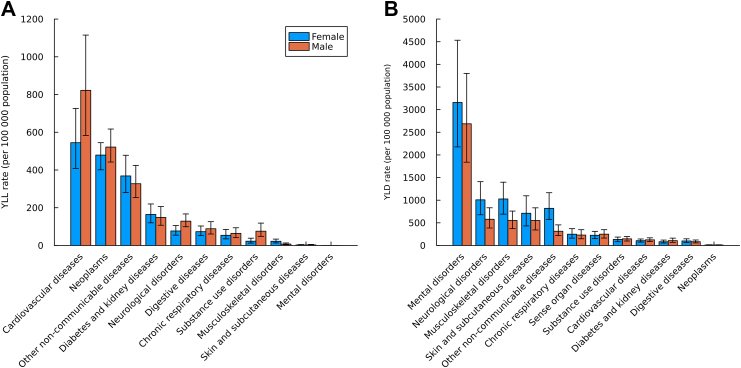
**YLL (A) and YLD (B) rates per 100,000 population due to level 2 non-communicable diseases in adolescents and young adults aged 10–24 years in MENA, by sex, 2023**. Note: YLDs = years lived with disability. YLLs = years of life lost. This aggregate cause contains the following level 3 causes: congenital birth defects; urinary diseases; gynaecological diseases; haemoglobinopathies and haemolytic anaemias; endocrine, metabolic, blood, and immune disorders; and oral disorders. Other non-communicable diseases include Congenital birth defects; Urinary diseases and male infertility; Gynecological diseases; Hemoglobinopathies and hemolytic anemias; Endocrine, metabolic, blood, and immune disorders; Oral disorders.

YLL rates were substantially higher in males (8593.6 [95% UI 8201.8–8978.2]) than in females (4862.1 [4586.6–5153.1]), with the greatest sex gap observed in the 20–24-year age group, corresponding to a male-to-female ratio of 2.1:1 (Fig. 3; Appendix Fig. S5). Following injuries, NCDs represented the second largest contributor to YLLs among young people, accounting for 37.0% (34.1–40.5) of total YLLs in females and 25.0% (22.1–29.6) in males. Age-sex patterns were consistent across all age subgroups 10–14, 15–19, and 20–24-year subgroups (Appendix Table S3). Age–sex differences in NCD Level 2 YLLs are detailed in Appendix Fig. S6. Notably, significant sex disparities were observed for several Level 3 causes, particularly drug use disorders (71.18 [44.96–113.50] in males vs 20.91 [11.24–36.89] in females) and other neurological disorders (42.11 [29.71–60.32] in males vs 14.50 [8.41–23.59] in females; Appendix Fig. S7).

Country-level variation in NCD-related YLLs were marked. In 2023, the highest rates were recorded in Sudan (2837.9 [95% UI 2221.9–3428.8]), Egypt (2559.4 [2118.0–3038.7]), Morocco (2257.4 [1852.4–2732.9]), Iraq (2244.0 [1941.3–2590.5]), and Afghanistan (2193.5 [1737.0–2749.6]) (Appendix Fig. S8). YLL rates in Sudan and Egypt exceeded more than threefold the YLL rates observed in Qatar and Kuwait, which recorded the lowest values in the region. Cardiovascular diseases were the leading cause of YLLs in 13 countries (Sudan, Egypt, Morocco, Afghanistan, Libya, Yemen, Algeria, Saudi Arabia, Syria, Oman, UAE, Qatar, and Kuwait), while neoplasms predominated in seven (Iraq, Iran, Lebanon, Tunisia, Türkiye, Jordan, and Palestine). Bahrain was the only country in which other NCDs ranked first. When compared with the regional mean (2003.3 [1824.2–2211.1]), five countries—Sudan, Egypt, Morocco, Iraq, and Afghanistan—had significantly higher YLL rates due to NCDs, whereas nine had significantly lower rates (Tunisia, Türkiye, Oman, Bahrain, Jordan, Palestine, UAE, Qatar, and Kuwait).

Between 1990 and 2023, YLL rates due to NCDs declined by 34.2% (95% UI –44.3 to −23.2), although this downward trend slowed after 2020, with a slight increase during and after the COVID-19 period (Appendix Fig. S9; Appendix Table S4). Among Level 2 causes, only mental disorders and substance use disorders increased during this period, with mental disorders rising from 0.02 (0.01–0.06) per 100,000 in 1990 to 0.05 (0.02–0.12) in 2023, a relative change of 128.0% (−46.5 to 1354.2), entirely attributable to eating disorders. Substance use disorders increased from 44.62 (31.17–62.31) to 49.96 (35.71–71.97) per 100,000, a 12.0% (−34.5 to 84.9) change (Appendix Table S4).

### Years lived with disability (YLDs)

In 2023, the all-cause YLD rate among adolescents and young adults aged 10–24 years in the MENA region was 8693.4 (95% UI 6485.8–11,210.0) per 100,000 population, with rates increasing progressively with age (Appendix Fig. S10). NCDs were the dominant Level 1 cause of disability, accounting for 6604.5 (4806.0–8636.2) per 100,000 population, corresponding to 76.0% (71.5–79.6) of all YLDs (Appendix Table S2). Among Level 2 causes, mental disorders were the leading contributors to YLDs at (2916.2 [2013.4–4165.8] per 100,000 population). At Level 3, anxiety disorders (1171.1 [650.2–1951.6]), depressive disorders (944.7 [614.6–1356.2]), and headache disorders (669.1 [446.2–953.9]) per 100,000 population were the top causes (Appendix Table S5).

YLD rates were consistently higher among females (9904.3 [95% UI 7321.6–12,781.7]) than males (7546.6 [5694.1–9661.6]) (Appendix Fig. S10). Across all age subgroups, NCDs were the dominant Level 1 cause of YLDs, with the highest burden recorded in individuals aged 20–24 years (7563.1 [5600.9–9910.3] per 100,000 population; Appendix Table S2). Mental disorders were the leading Level 2 cause of YLDs across all three age groups (Appendix Fig. S11), with clear variation by sex and age (Fig. 3; Appendix Fig. S11). Significant sex differences were noted for Level 3 causes such as attention-deficit/hyperactivity disorder with higher values in males (166.4 [97.9–250.8]) than in females (50.5 [30.1–78.7]; Appendix Fig. S12).

National YLD rates for NCDs ranged from 6046.4 (95% UI 4519.0–7968.2) in Qatar to 7490.9 (5406.7–10,014.6) in Iran (Appendix Fig. S13) although no statistically significant differences were observed relative to the regional mean. In every MENA country, mental disorders were the leading cause of YLDs among young people.

Between 1990 and 2023, all-cause YLD rates showed an initial decline from 8745.0 (6672.6–11,532.6) per 100,000 population in 1990–8081.3 (6155.3–10,609.8) in 2019, followed by an increase to 8672.3 (6614.9–11,265.8) in 2020 and 8693.4 (6485.8–11,210.0) in 2023 (Appendix Fig. S10). NCD-specific YLDs remained nearly unchanged between 1990 and 2019 (6079.1 [4450.2–7917.9] vs 6074.0 [4530.5–7977.4] per 100,000), but increased notably after 2020, reaching 6425.7 (4750.1–8406.0) in 2020 and 6604.5 (4806.0–8836.2) in 2023 (Appendix Fig. S14; Appendix Table S5). The largest rise among Level 2 NCD causes, was observed for diabetes and kidney diseases (98.4% [79.5–116.6]), driven primarily by an increase in diabetes at Level 3 (127.9% [107.9–149.4]; Appendix Table S5).

### Disability-adjusted life-years (DALYs)

In 2023, the all-cause DALY rate among adolescents and young adults aged 10–24 years in the MENA region was 15,471.9 (95% UI 13,083.2–17,953.9) per 100,000 population (Appendix Fig. S15A). Across Level 1 causes, injuries were the leading contributor to DALYs among males aged 20–24 years, whereas NCDs were the dominant cause of DALYs in both sexes and all other age categories. CMNN causes continued a gradual decline over the study period.

From 1990 to 2023, DALY rates from injuries increased sharply between 2010 and 2015, coinciding with periods of political unrest across the region. By contrast, NCD-related DALYs declined steadily across the same period, with no disruption observed during the Arab Spring years. Among females, NCDs consistently accounted for the highest DALY burden throughout the three-decade period. Decade-specific estimates and percentage changes are presented in Appendix Table S8B, and temporal trends are shown in Appendix Fig. S15B.

In 2023, NCDs accounted for 55.0% (51.0–59.9) of the total DALY burden in people aged 10–24 years (Appendix Table S2). At Level 2, mental disorders were the leading cause of NCD-related DALYs at 2916.3 (2013.4–4165.9) per 100,000 population (Table 1), representing 33.8% of the total NCD DALY burden. At Level 3 anxiety disorders (1171.1 [650.2–1951.6]), depressive disorders (944.7 [614.6–1356.2]), and headache disorders (669.1 [446.2–953.9]) per 100,000 population were the top contributors.

Total DALY rates increased with age in both sexes and were generally higher in males than females, except for adolescents in the 10–14-year age group where rates were comparable (Appendix Fig. S15). Sex disparities were greatest in the 15–19 and 20–24-year age groups. Significant sex differences at Level 2 were observed for other NCDs (Appendix Fig. S16), while no statistically significant sex differences were detected at Level 3 (Appendix Fig. S17).

**Fig. 4:**
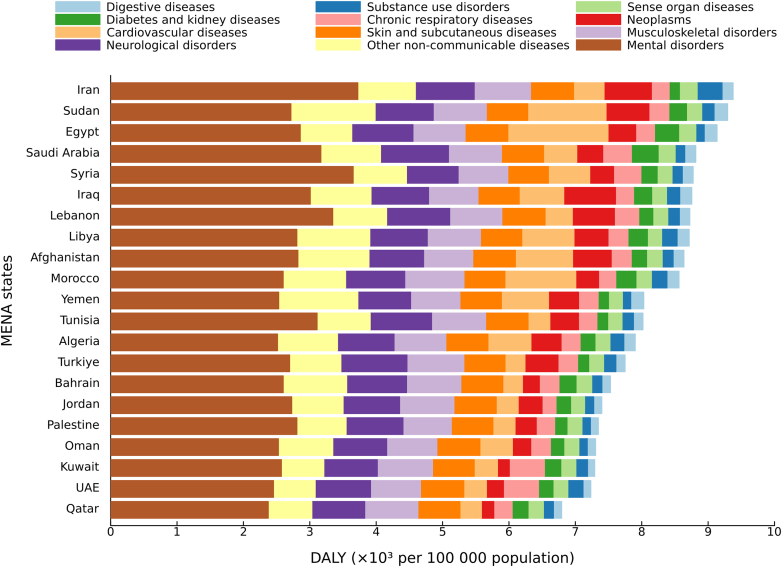
**DALY rate per 100,000 population due to level 2 non-communicable diseases in adolescents and young adults aged 10–24 years in both sexes, MENA region by country, 2023**. Note: DALY = disability-adjusted life-year. ∗This aggregate cause contains the following level 3 causes: congenital birth defects; urinary diseases; gynaecological diseases; haemoglobinopathies and haemolytic anaemias; endocrine, metabolic, blood, and immune disorders; oral disorders. Other non-communicable diseases include Congenital birth defects; Urinary diseases and male infertility; Gynecological diseases; Hemoglobinopathies and hemolytic anemias; Endocrine, metabolic, blood, and immune disorders; Oral disorders.

In 2023, NCD-related DALY rates varied across countries, ranging from 6794.1 (95% UI 5311.8–8744.6) in Qatar to 9377.5 (7251.7–11,881.6) in Iran (Fig. 4). Mental disorders were the leading Level 2 cause of NCD DALYs in every MENA country, accounting for 18.7% (14.9–23.6) of the total regional NCD DALY burden. Notable country-level differences compared with the regional mean were observed for specific Level 2 causes: cardiovascular diseases were higher in Egypt, diabetes and kidney diseases were higher in Saudi Arabia, neoplasms were higher in Iraq and Iran, and substance use disorders were higher in Iran (Appendix Fig. S18).

Between 1990 and 2023, NCD-related DALY rates decreased modestly from 9128.9 (7381.0–10,894.4) to 8607.8 (6747.9–10,602.0) per 100,000 population, representing an overall reduction of 5.7% (95% UI –14.3 to 4.2). A long-term decline through 2019 (8034.8 [6491.0–9843.0]) reversed slightly during and after the COVID-19 period, reaching 8377.7 (6716.5–10,254.9) in 2020 (Table 1; Appendix Fig. S19). Among Level 2 causes, the largest reductions were observed in digestive diseases (−44.1% [–55.0 to −26.2]) and cardiovascular diseases (−40.9% [–56.6 to −19.9]) (Table 1). Country-level time series of mental-disorder YLDs and YLLs by sex for selected conflict-affected countries are shown in Appendix Fig. S20.

### Correlation between SDI and NCD DALY rates

In 2021, the SDI among MENA countries ranged from 0.34 in Afghanistan to 0.85 in the United Arab Emirates, Qatar, and Kuwait (Appendix Table S6). A clear inverse relationship was observed between SDI and NCD burden among adolescents and young adults. Significant negative correlations were identified between SDI and DALY rates for digestive diseases (rs = −0.65, p = 0.002), cardiovascular diseases (rs = −0.63, p = 0.003), neoplasms (rs = −0.50, p = 0.021), and other non-communicable diseases (rs = −0.54, p = 0.013), indicating lower DALY burdens in countries with higher socio-demographic development. No significant positive correlations were found between SDI and any Level 2 NCD causes (Appendix Table S7; Appendix Fig. S21).

## Discussion

Health among adolescents and young adults in the MENA region is defined by a burden of injury and chronic disease. Injuries remain the leading cause of death, reflecting vulnerability to sociopolitical instability and recurrent conflict, while NCDs have emerged as the largest contributor to overall health loss measured by DALYs. Communicable, maternal, neonatal and nutritional conditions have continued to decline, signaling sustained gains in infectious disease control. Together these shifts illustrate a regional epidemiological transition: acute crises precipitate short-term surges in injury mortality, yet the enduring challenge is the steady accumulation of chronic, disabling conditions among young people.^15^^,^^16^

To the best of our knowledge, this analysis represents the first comprehensive assessment of NCD burden among adolescents and young adults across 21 MENA countries using GBD 2023 estimates. Over the past three decades, NCD mortality among young people in the MENA region has declined substantially, suggesting improvements in clinical care, essential health services and overall system capacity. However, the disability burden from NCDs has remained largely unchanged, driven predominantly by mental disorders, alongside musculoskeletal and metabolic conditions, creating a mortality-disability paradox in which survival has improved but gains in health life-years have not kept pace. Similar patterns have been reported in analyses of adolescents and young adults in regions including Asia and Europe.^5^^,^^6^^,^^17^ This imbalance underscores persistent gaps in early detection, rehabilitation, and long-term management of chronic illness during adolescence.

Between 1990 and 2019, declines in premature mortality and years of life lost (YLLs) especially from cardiovascular and digestive diseases, reflected expanded access to diagnostics, treatment and essential services.^6^ However, after 2020, these gains stalled or reversed. Mortality and YLLs plateaued, while years lived with disability and overall DALYs rose, driven primarily by mental and metabolic disorders.^17^ These post-2020 increases are consistent with regional evidence of pandemic-related service disruptions, reduced health-seeking behavior and social stressors affecting young people. Previous research from the region confirms that COVID-19 widened adolescent mental health gaps and interrupted continuity of NCD care.

Mental disorders accounted for roughly one-third of total NCD DALYs among adolescents and young adults in the region, consistent with evidence from conflict-affected MENA settings where mental ill health is widespread. Systematic reviews and national studies in Syria, Iraq, Palestine, Libya, Lebanon, Yemen, and Sudan have reported adolescent post-traumatic stress disorder, anxiety and depression prevalence exceeding 30% in populations exposed to war displacement and prolonged insecurity.^18^, ^19^, ^20^ Evidence also suggests that indirect exposure to regional conflict can adversely affect youth mental health in neighboring countries, with studies from Egypt and Jordan showing higher levels of anxiety, depression, and psychological distress among young adults associated with perceived disruption from regional wars, media exposure, and personal connections to affected populations.^21^^,^^22^ Young people living through chronic instability experience compounding stressors, including family loss, educational disruption, and forced migration, which elevate mental health risk well into adulthood. These findings suggest that mental health among young people in MENA serves as both a health indicator and a measure of societal resilience.

The particularly high mental-health burden in countries such as Iran, Iraq, and Syria illustrates how geopolitical stressors and historical trauma influence well-being among young people. In Iran, robust mental health surveillance may increase detection,^23^ but chronic socioeconomic strain and the compounded effects of international sanctions exacerbate household well-being.^24^, ^25^, ^26^ In Iraq, persistently high rates of mental disorders reflect the cumulative effects of war, displacement, and instability among adolescents and young adults.^25^^,^^27^^,^^28^ These patterns emphasize that adolescent mental-health is both a medical and social indicator of societal resilience. Consistent with this interpretation, country-level time-series analyses did not show consistent short-term spikes in mental-disorder burden at the onset of individual conflicts, suggesting cumulative rather than episodic impacts of war on mental health in the region.^29^

Similar patterns have been reported in other GBD analyses of adolescents and young adults in South Asia, Western Pacific, and European Union, where mental disorders contribute minimally to mortality but account for a substantial share of disability and overall disease burden.^5^^,^^6^ Despite differences in absolute burden across regions, the consistency of these trends highlights that the predominance of mental disorders as a source of adolescent health loss is not unique to MENA and underscores the global need to prioritize prevention, early detection, and youth-responsive mental health services.

Sex and age disparities are equally pronounced.^6^ Males exhibit higher mortality and YLLs driven by cardiovascular disease and substance use related causes, while females bear greater disability, primarily from anxiety and depressive disorders.^30^ These differences arise from a complex interplay of biological, behavioral and sociocultural determinants.^31^ Adolescent boys are more likely to engage in high-risk behaviors such as smoking or substance use, consistent with regional evidence showing tobacco use as a major contributor to NCD burden,^32^ whereas girls face stigma, psychosocial stress and restricted access to mental health care. Addressing these disparities requires responsive policies that integrate mental health services into primary and school-based care, expand youth-friendly services and tackle stigma.^4^^,^^16^ The World Health Organization's, “Accelerated Action for the Health of Adolescents” (AA-HA!) framework, endorsed by WHO's Eastern Mediterranean Regional Office (EMRO) provides operational guide for this integration.^33^

Schools represent a critical yet underutilized platform for health promotion for young people in MENA. Evidence from Qatar shows that implementation of the WHO School Mental Health Package significantly improved teachers’ mental-health literacy, demonstrating feasibility at scale.^34^ Similar interventions in the United Arab Emirates and Saudi Arabia have strengthened the capacity of school nurses and counsellors to identify and support at risk adolescents and young adults.^35^ The evidence from school-based interventions in the GCC countries further indicates potential for reducing obesity and improving physical activity, but coverage across the MENA region remains uneven.^36^ Expanding school-based health education, nutrition, and mental health programs would directly target the leading causes of adolescent disability identified in this study.^17^^,^^37^

Country level variation in NCD burden among young people reflects persistent inequities across the region. Mortality and YLLs are highest in Sudan, Egypt, Morocco, Iraq and Afghanistan, where political instability and economic fragility constrain health system performance, while the lowest rates are observed in high-income Gulf states. The inverse relationship between the SDI and NCD DALYs, particularly for cardiovascular, digestive and neoplastic diseases, highlights the protective effects of education, income and demographic transition.^37^ Yet SDI alone cannot fully capture the effects of conflict, displacement and gender inequality, all of which uniquely shape adolescent health in the MENA region.

Rising metabolic risk among adolescents and young adults compounds these challenges. Regional estimates, document sharp increases in overweight and obesity prevalence across the GCC and North Africa, with early-onset type 2 diabetes emerging among youth.^38^ These findings are consistent with the observed rise in YLDs from diabetes and kidney disease and reflect rapid urbanization, dietary transitions and sedentary lifestyles.^39^ Without preventive action, these patterns are likely to translate into high adult NCD morbidity and mortality, placing further strain on health systems.

Reducing these disparities requires coordinated, multisectoral response extending beyond the health sector. Global and regional frameworks, such as Every Woman, Every Child, the Arab strategy for Maternal, Child and Adolescent Health, and UNICEFs Adolescent Development and Participation initiative, emphasize integrated approaches across education, social protection and youth engagement. Several countries form the MENA region have begun operationalizing these priorities such as, the UAE's Masar project which has reached more than 130,000 students with lifestyle education^40^; Saudi Arabia's Vision 2030 which includes integrated adolescent health and NCD surveillance; and Qatar's National Health Strategy which aligns with WHO's AA-HA! framework to expand school and community based mental health services.^33^ Sustained implementation, equitable coverage and robust monitoring are essential to ensure these efforts translate into improved health outcomes.

Persistent sex differences in mortality and disability likely reflect sex-specific barriers to care and risk exposures in the region. Higher premature mortality among males may be influenced by greater exposure to conflict and injuries, while the higher disability burden among females, particularly from mental disorders, may be shaped by sociocultural norms and barriers to accessing timely and appropriate care. While conflict and instability may partly explain NCD burden among young people in MENA, the similarity of patterns across regions indicates that broader structural, behavioral, and health-system determinants also warrant investigation.^5^^,^^6^^,^^17^

This study has limitations inherent to GBD 2023 estimates. Data completeness and quality vary across countries and age groups, with underreporting particularly for younger adolescents and non-fatal outcomes. Mental disorders may be underestimated due to classification constraints, as self-harm and violence are captured under injuries, while stigma and low service coverage contribute to underdiagnosis. Conflict related displacement and migration further complicate case ascertainment and indices such as SDI may not fully reflect the sociopolitical determinants shaping health among adolescents and young adults in MENA. Although GBD provides the most comprehensive comparable estimates available, estimates of mental disorder burden rely on model-based approaches rather than derived from direct national surveillance.

Despite these limitations, the findings reveal that NCD prevention and care for young people in MENA remain fragmented, reactive and under-resourced. Primary-care systems often lack adolescent-focused screening and counselling, while school health services are consistently developed and insufficiently integrated with health systems. The consequences extend beyond health to the region's economic and social stability. Chronic conditions emerging in adolescence and young adulthood, such as obesity, diabetes, anxiety and depression, persist into adulthood, increasing premature cardiovascular risk and reducing workforce productivity. Without decisive action, the MENA region risks transforming its demographic “youth dividend” into a chronic disease dividend” with escalating healthcare costs and constrained economic growth.^41^

Investing in health among young people offers substantial long-term returns. Strengthening primary care, scaling school and community-based interventions and prioritizing mental health can transform their well-being and advance regional development.^33^^,^^42^^,^^43^ Embedding NCD prevention with universal health coverage and education policy is critical to ensure the region's largest generation of young people transition into adulthood healthy, productive and resilient.

Health among young people in the MENA region is at a critical crossroads. While mortality from non-communicable diseases has fallen markedly over the past three decades, disability linked to chronic and mental disorders continues to rise. Conflict, displacement, and social instability have compounded these vulnerabilities, leaving young people exposed to persistent psychological distress and widening inequalities in access to care. At the same time, rapid urbanization, unhealthy diets, and sedentary lifestyles are fueling a growing epidemic of metabolic risk in youth. These intersecting challenges demand a coordinated regional response; one that embeds adolescent NCD prevention and mental-health promotion within schools, primary care, and universal health coverage. Investing in these systems during adolescence and young adulthood will not only improve health and wellbeing but also secure the region's long-term social and economic resilience.

## Contributors

DK and BS conceived and designed the study. DK collected the data. DK, BS, AHM, and ANH directly accessed and verified the underlying data. DK and ANH analyzed the data. DK, AS, BS drafted the manuscript. AHM, and IE provided critical input and revisions. All authors reviewed and approved the final version of the manuscript.

## Data sharing statement

This study follows the Guidelines for Accurate and Transparent Health Estimates Reporting (GATHER). To download citations and metadata for the input data sources used in the GBD 2023 analyses presented in this study, please visit the GBD 2023 Sources Tool (https://ghdx.healthdata.org/gbd-2023/sources).

## Editor note

The Lancet Group takes a neutral position with respect to territorial claims in published maps and institutional affiliations.

## AI declaration

ChatGPT was used to assist with improving the grammar and clarity of the manuscript. All AI-assisted content was reviewed and edited by the authors, who take full responsibility for the originality, accuracy, and integrity of the work.

## Declaration of interests

None.
