# Supplementary material for: Burden of non-communicable diseases among adolescents and young adults aged 10–24 years in Middle East and North Africa, 1990–2023: a systematic analysis of the Global Burden of Diseases Study 2023
Source: eClinicalMedicine. 2026 Mar 13;93:103827. doi: 10.1016/j.eclinm.2026.103827 (PMC12995710; doi:10.1016/j.eclinm.2026.103827)
Supplement: Supplementary materials [file mmc1.docx]

**Supplementary materials**

**Burden of non-communicable diseases among adolescents aged 10–24 years in Middle East and North Africa, 1990–2023: a systematic analysis of the Global Burden of Diseases Study 2023**

**Appendix Figure 1A. All-cause mortality rates per 100,000 population, in people aged 10–24 years in MENA region from 1990 to 2023 by sex**

**
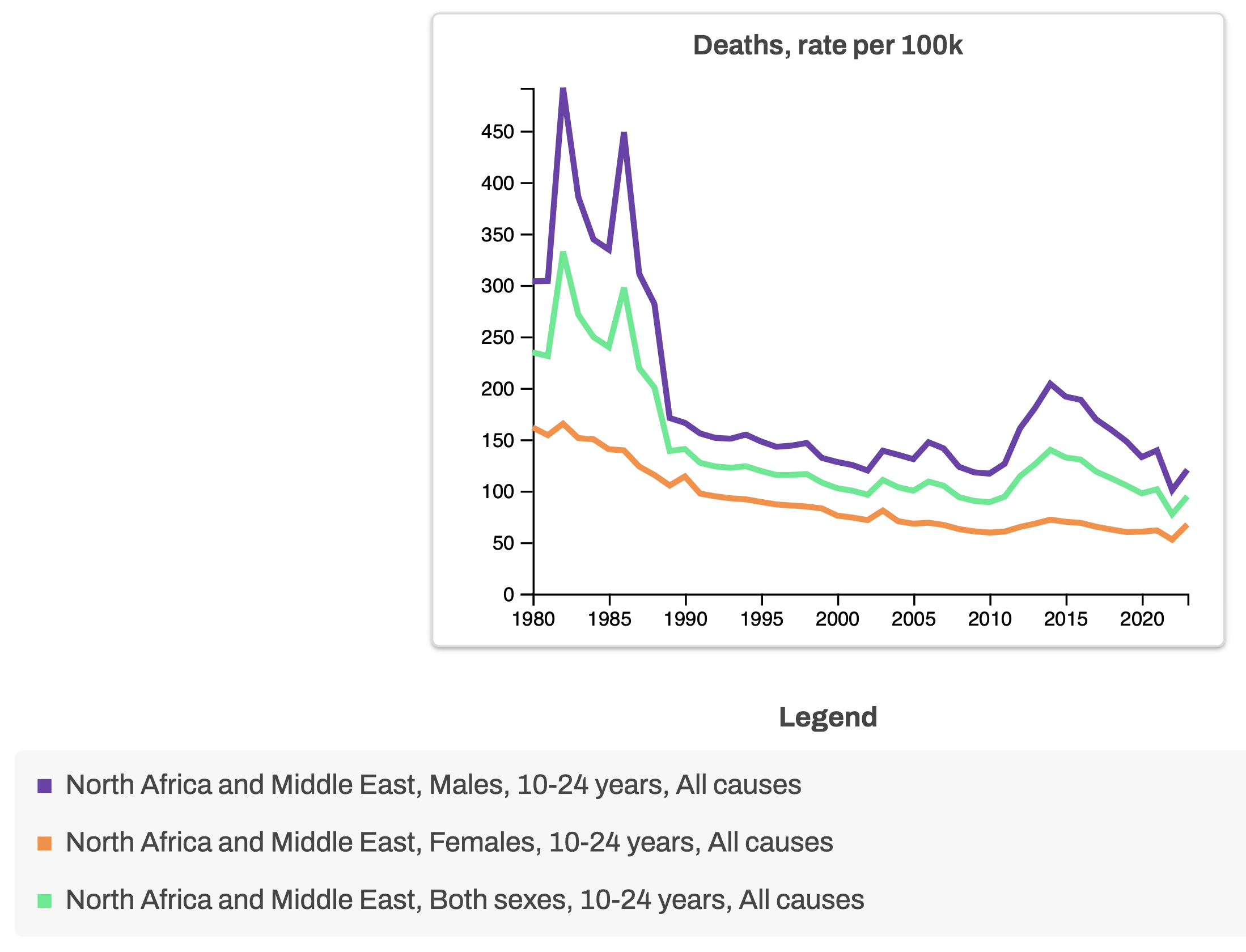
**

**
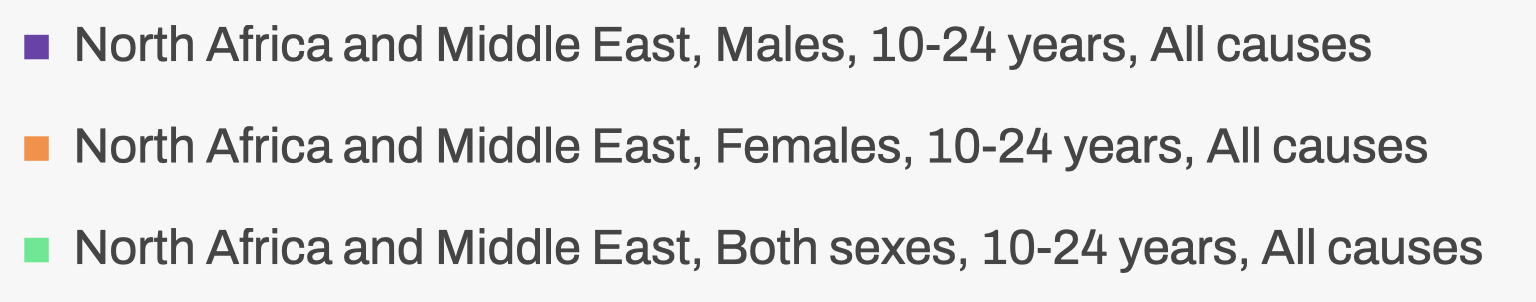
**

Note:

- Values for 2023: 94.6 (95%UI: 90.5-97.9) for both sexes, 120.4 for males and 67.3 per 100,000 population
- In 2023, among 10–24 years in MENA, injuries accounted for 96 645.80 (61.2%) of all deaths, followed by NCDs with 46 466.59 (29.4%), and communicable, maternal, neonatal, and nutritional (CMNN) causes with 14 873.04 (9.4%). The total all-cause mortality rate was 157 985.43 (100%).

**Appendix Figure 1B. Mortality rates per 100,000 population due to Level 1 cause, in people aged 10–24 years, in MENA region from 1990 to 2023**

**A. Both sexes**

**
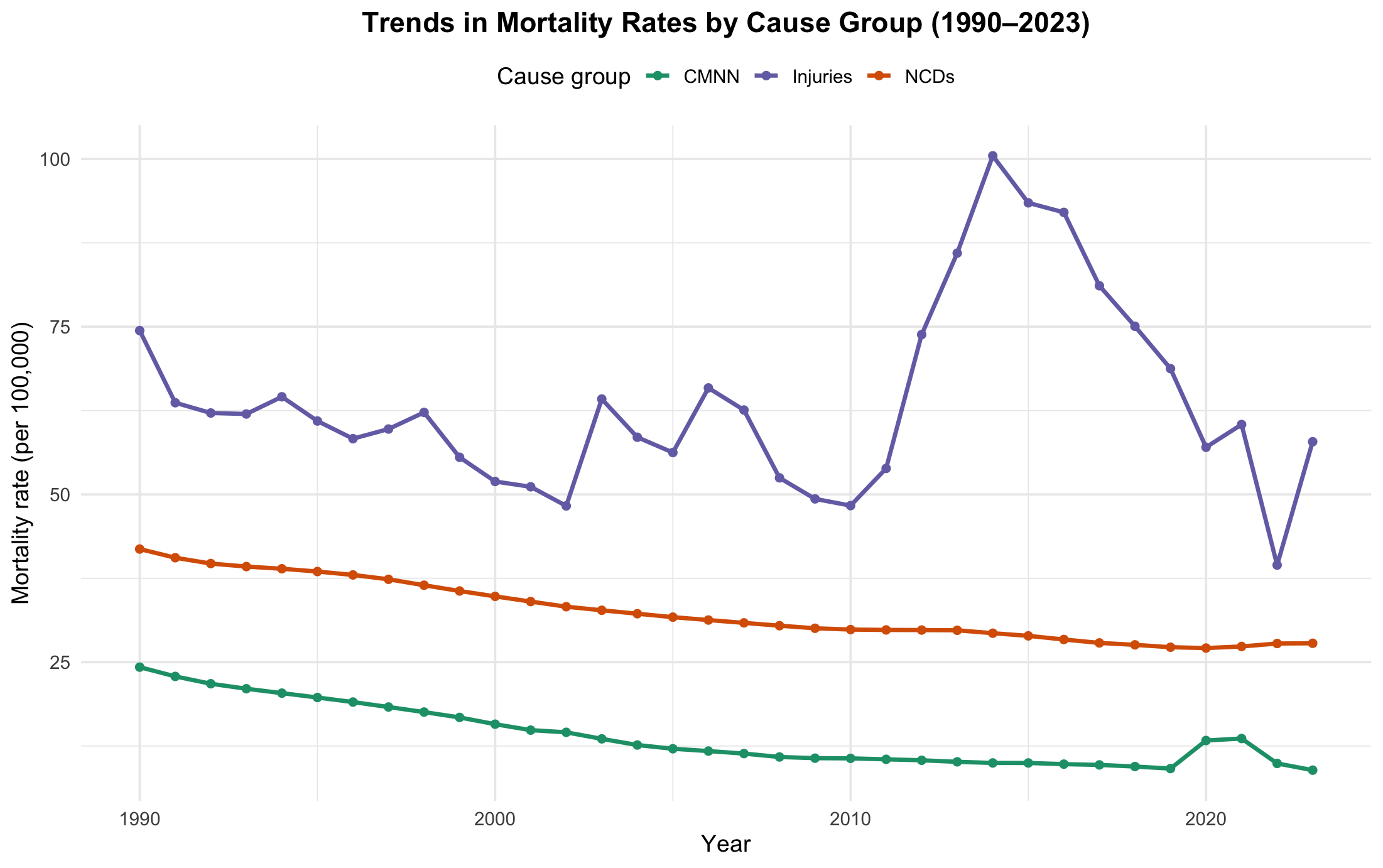
**

**B. Males and females**

**
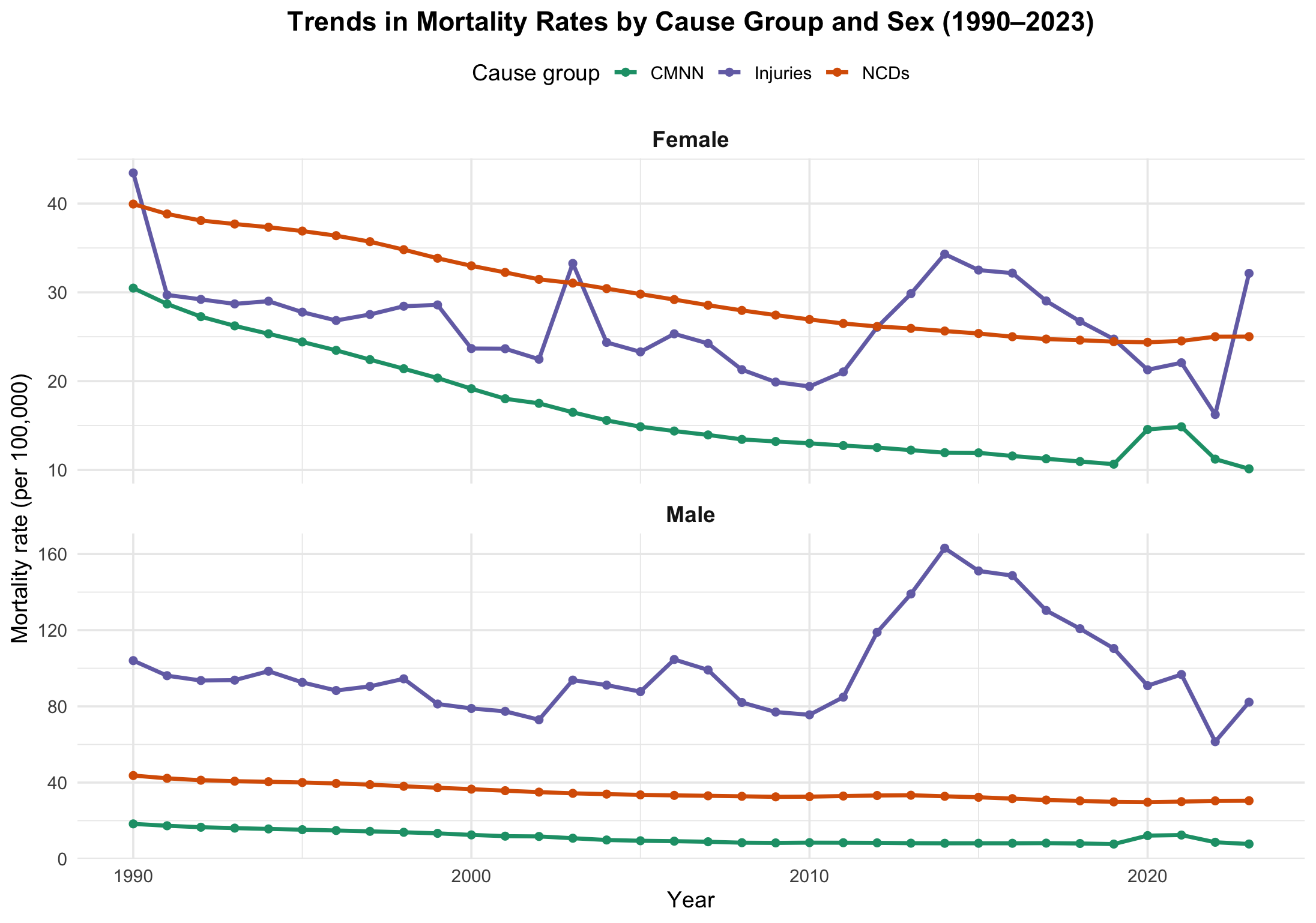
**

**Appendix Figure 2. All-cause mortality rates per 100,000 population, in people aged 10–24 years from 1990 to 2023 by sex and age group in MENA**

**(A)**

**
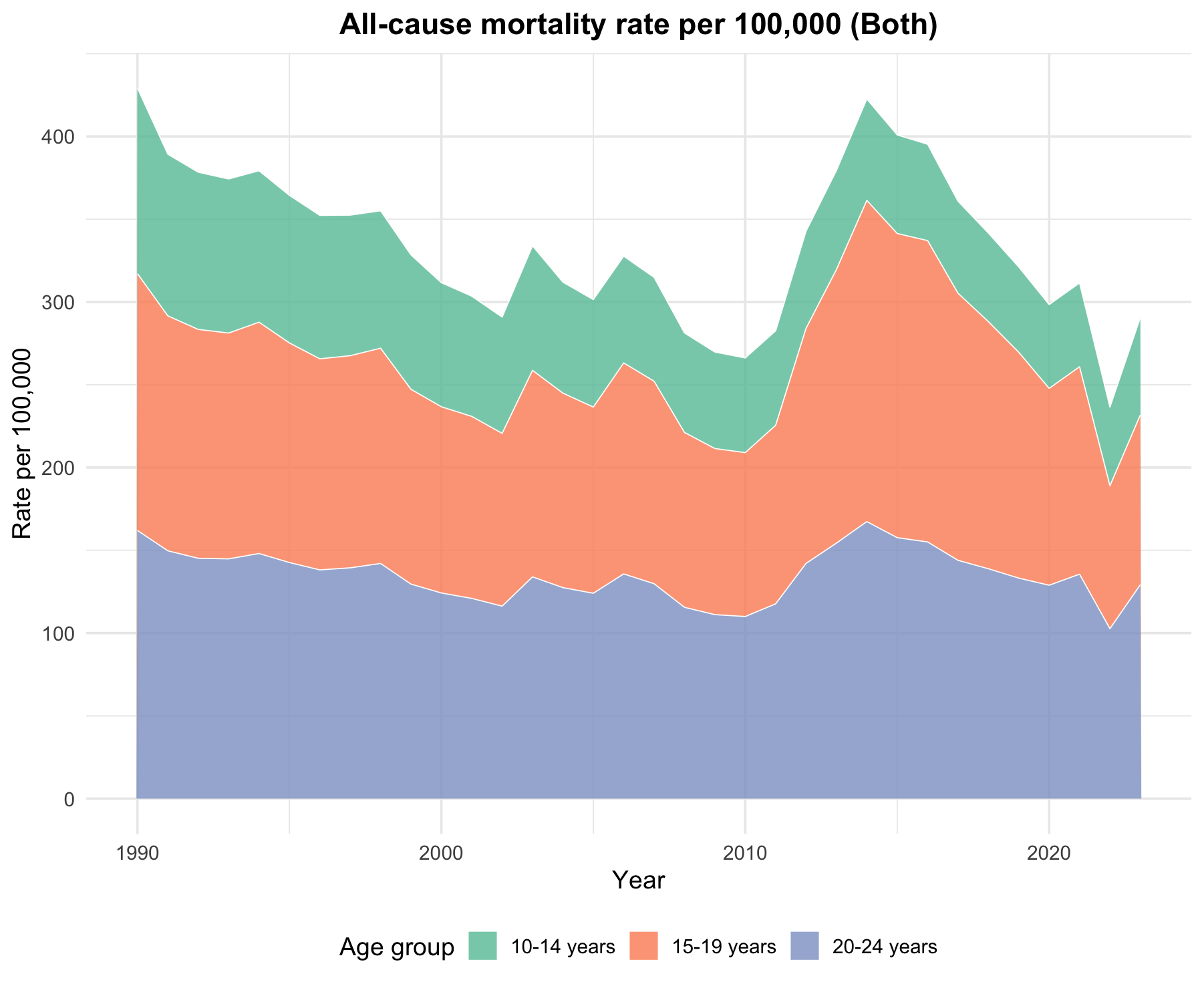
**

**(B)**

**
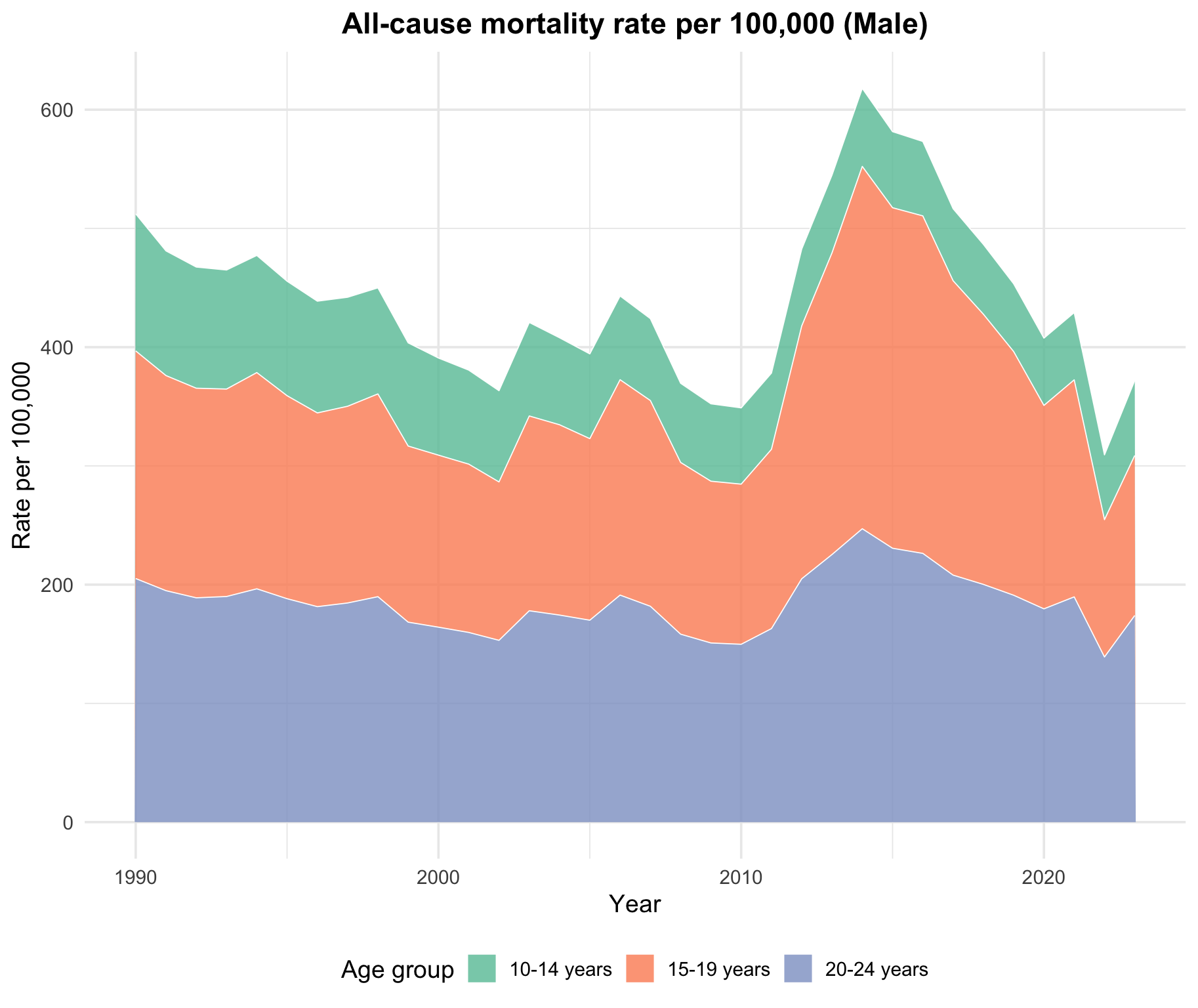
**

**(C)**

**
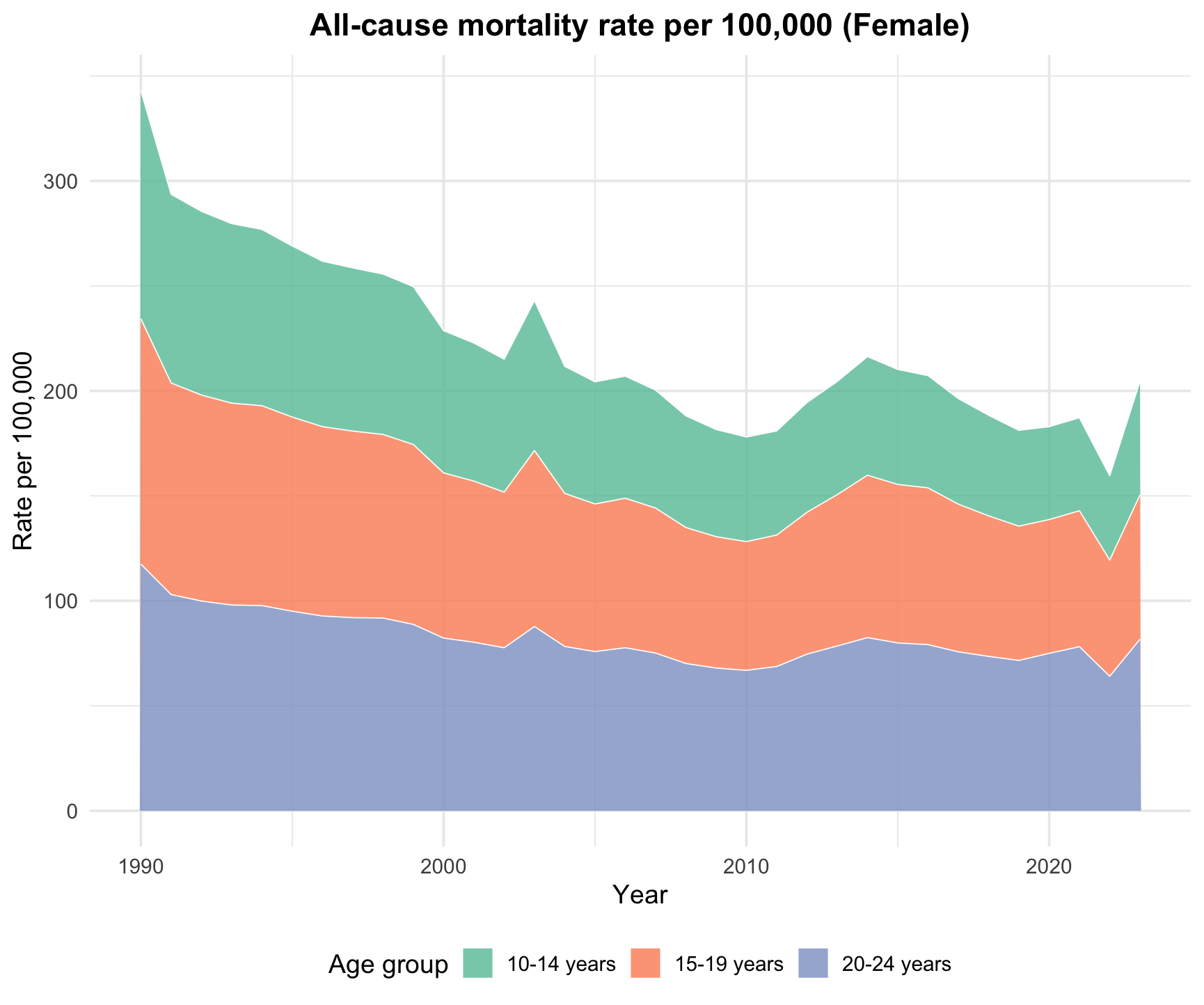
**

**Appendix Figure 3. Mortality rates per 100,000 population, level 2 NCDs causes, by age group and sex, in MENA, in 2023**

**
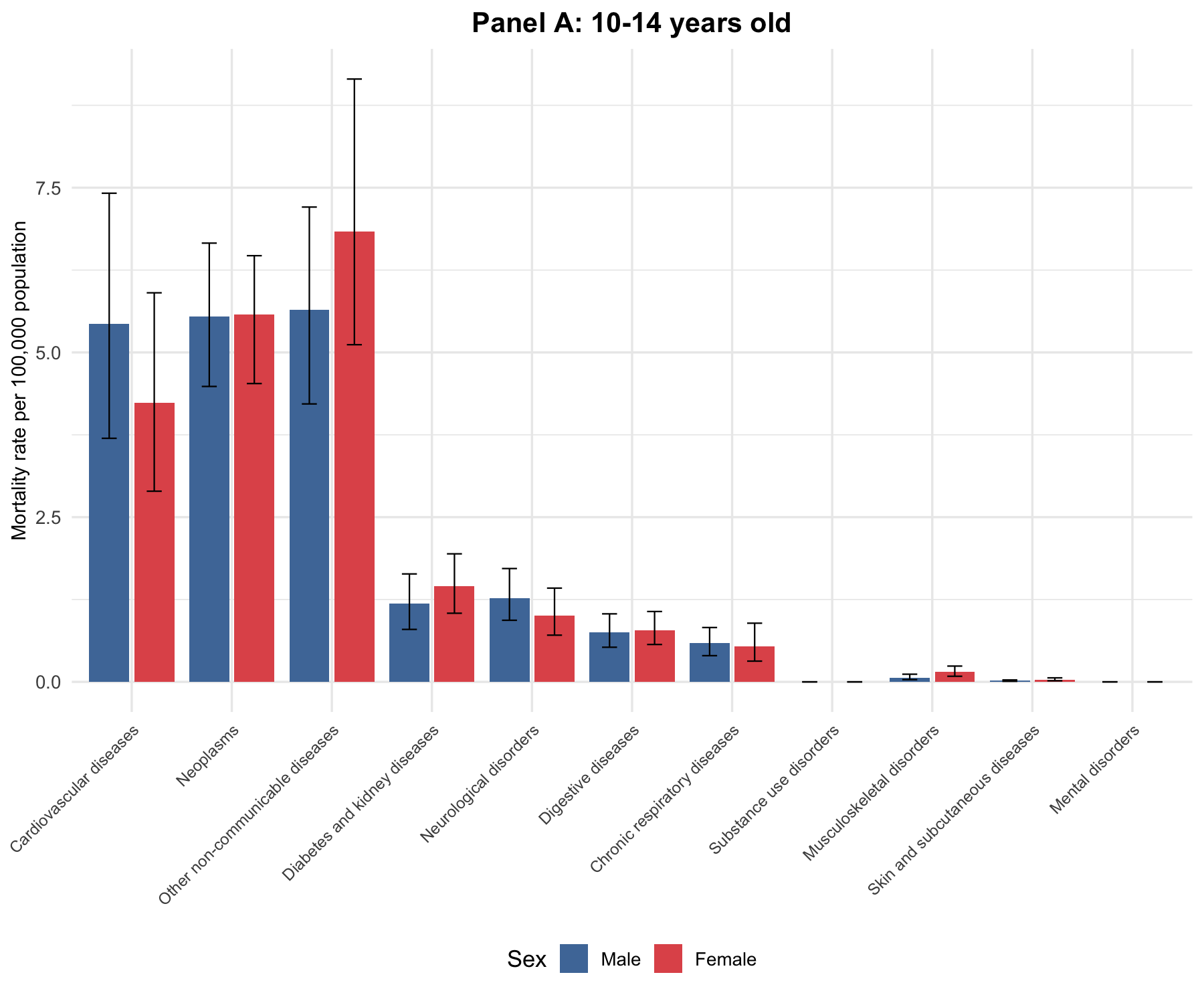
**

**
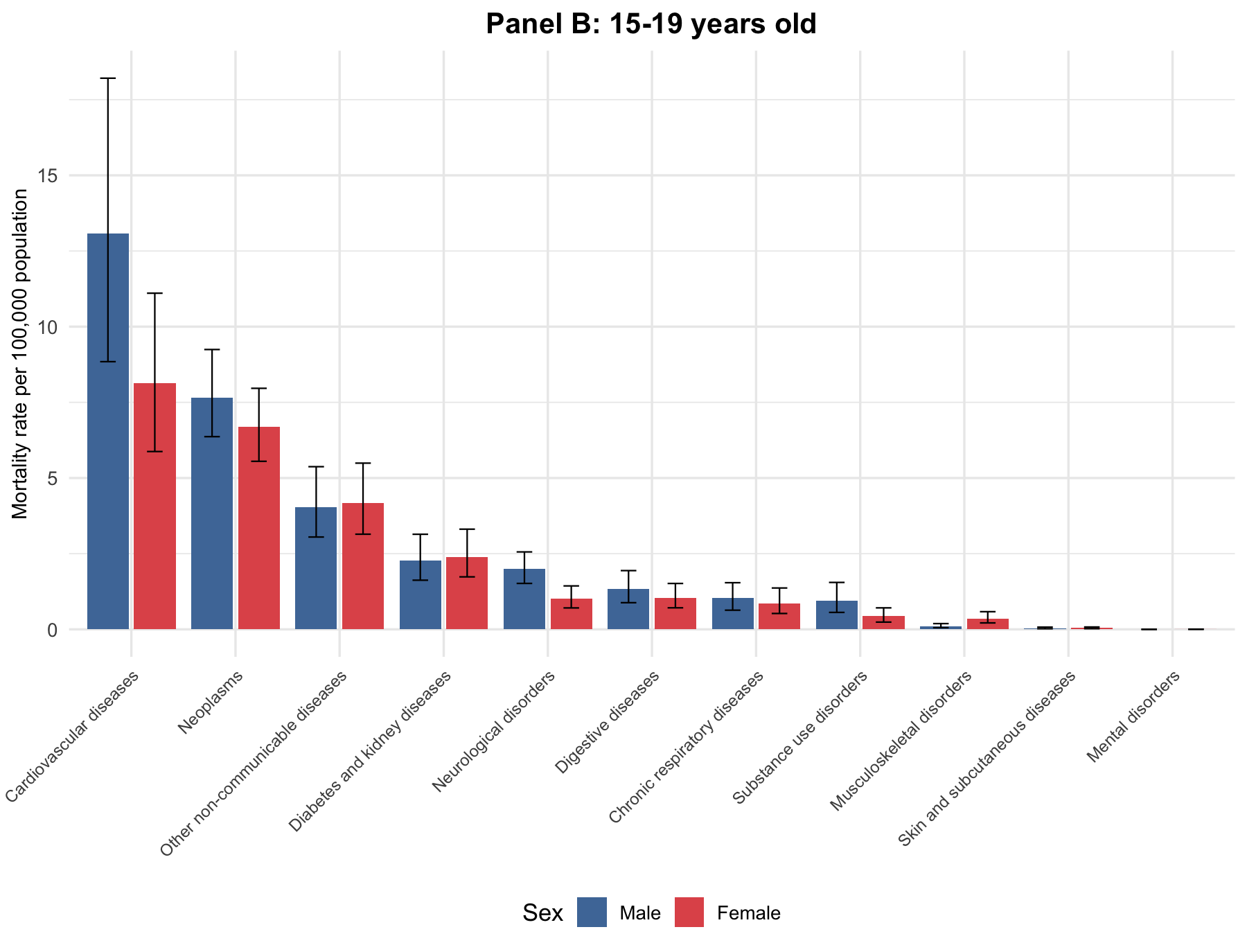
**

**
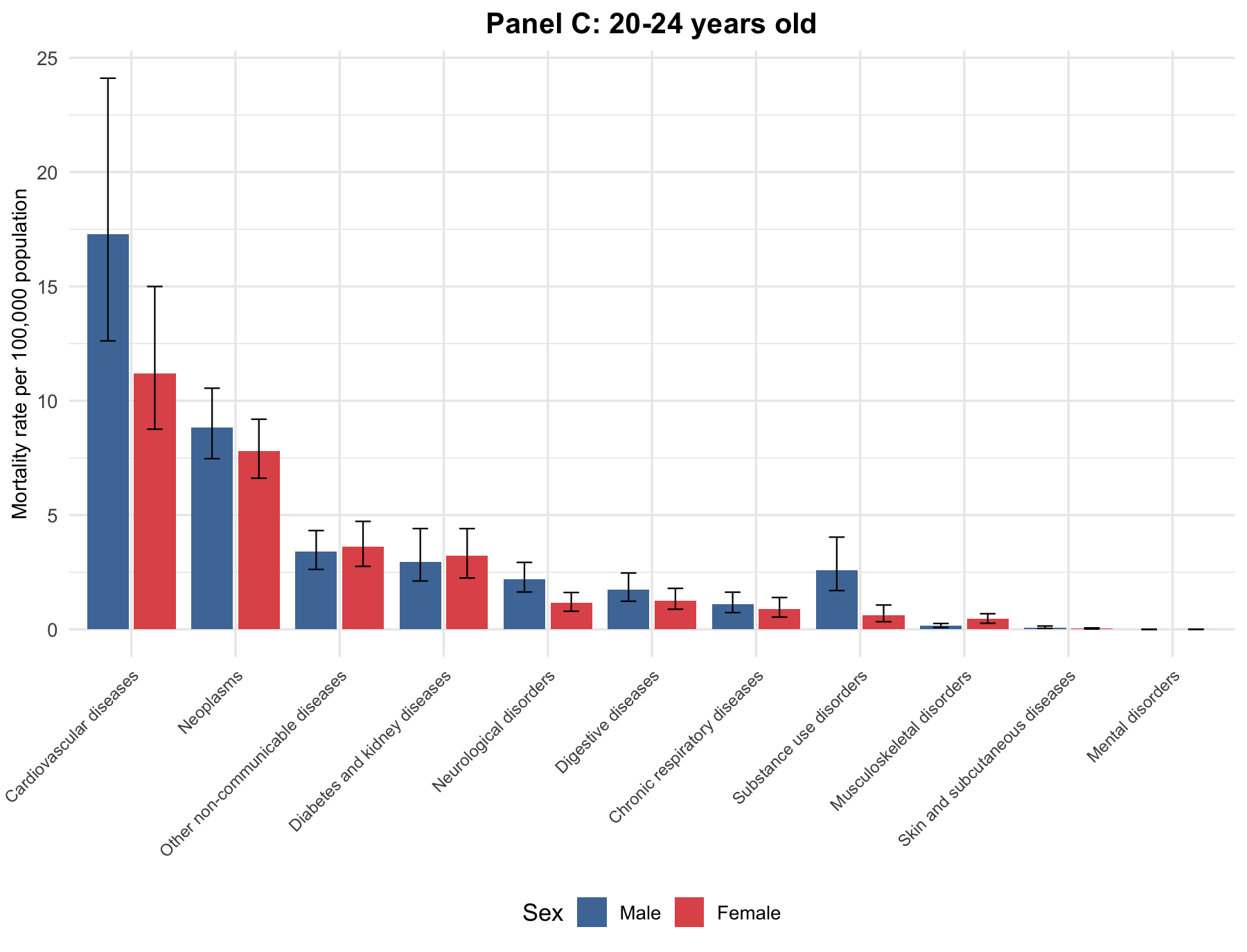
**

**Appendix Figure 4. Mortality rates per 100,000 population, NCDs level 2 causes, 10-24 years old, both sexes, in MENA, trend from 1990-2023**


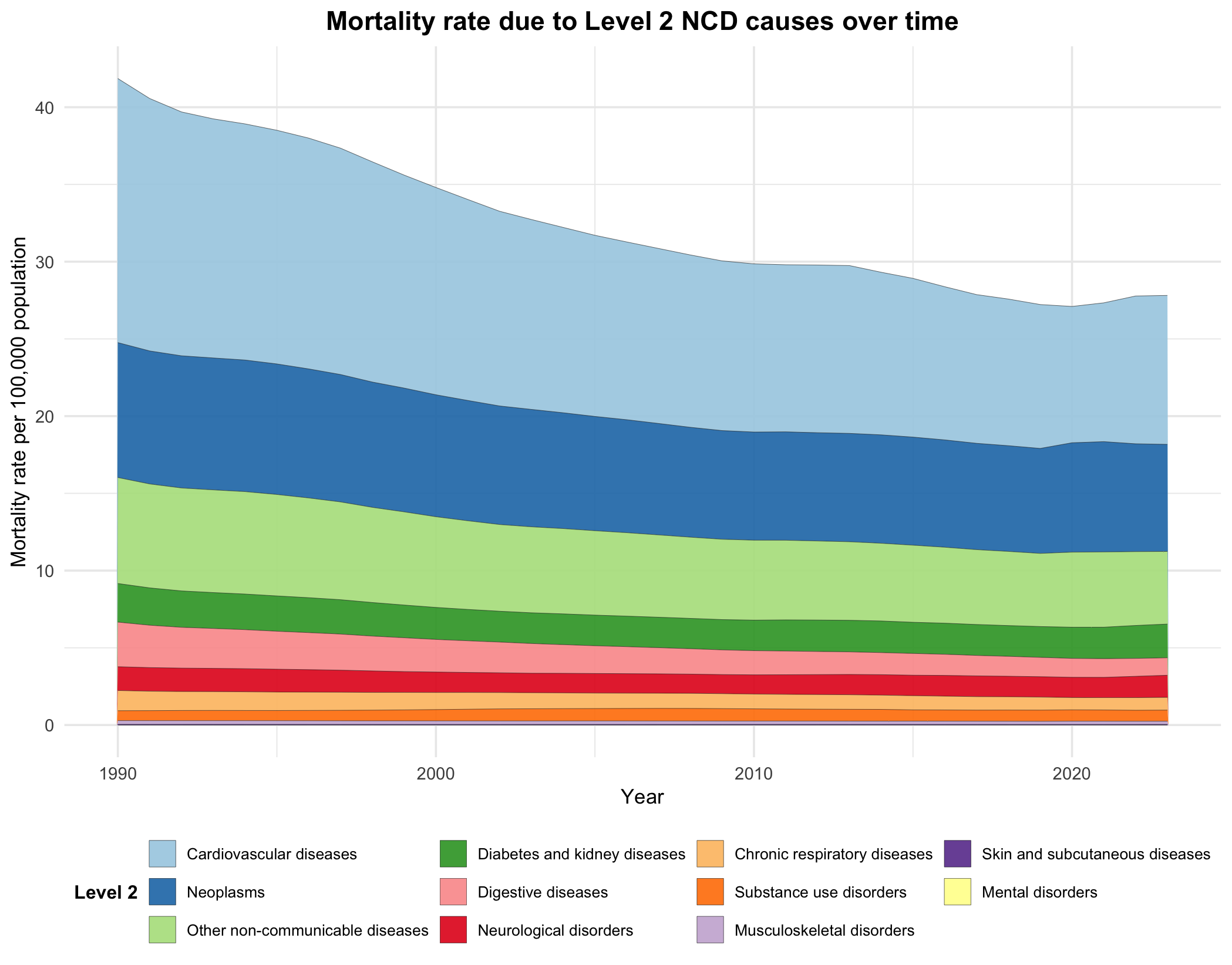


*Note: This aggregate cause contains the following Level 3 causes: congenital birth defects; urinary diseases; gynaecological diseases; haemoglobinopathies and haemolytic anaemias; endocrine, metabolic, blood, and immune disorders; oral disorders.

**Appendix Figure 5. All-cause YLL rates per 100,000 population, in people aged 10–24 years in MENA from 1990 to 2023 by sex and age group**

**
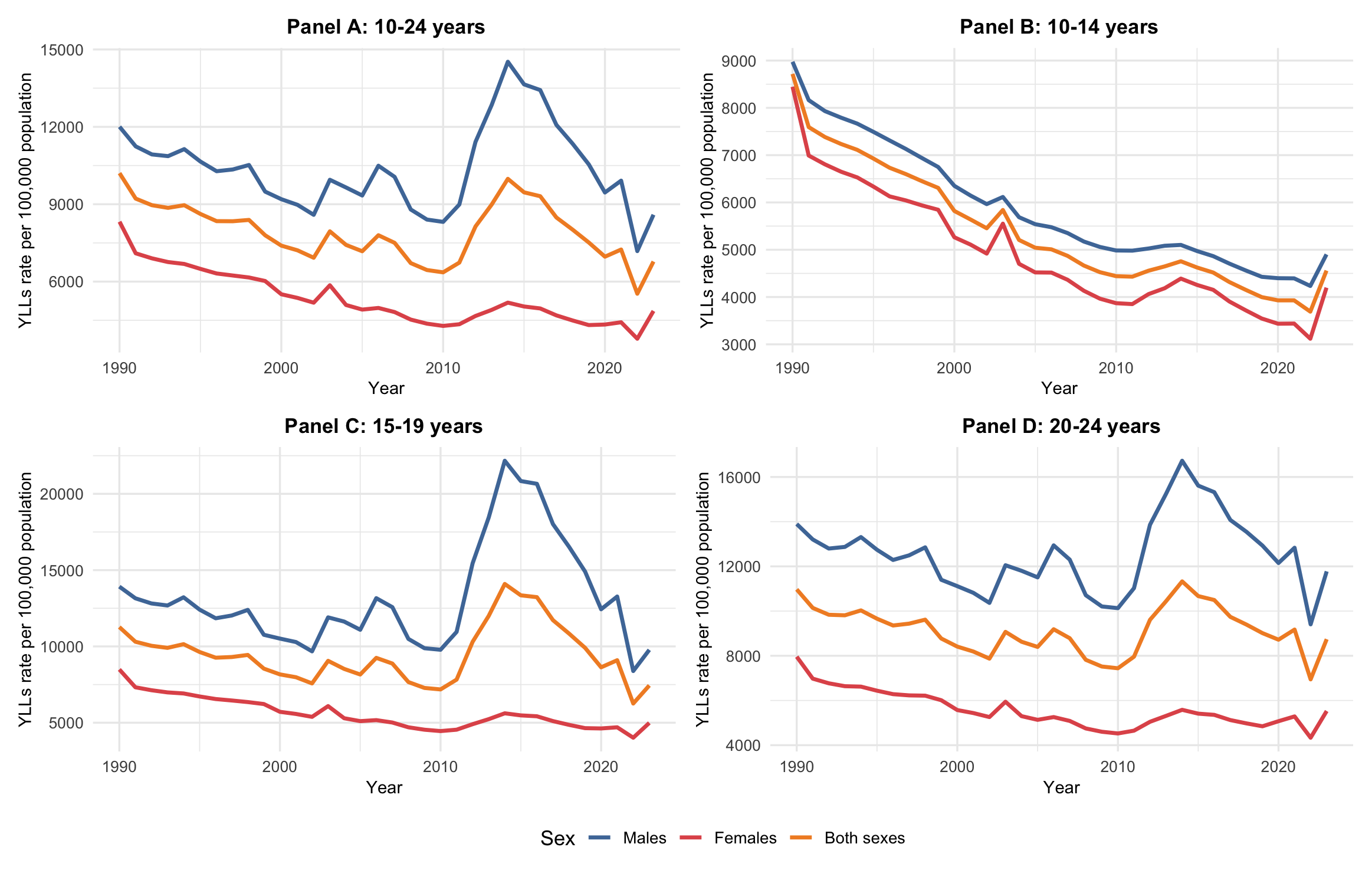
**

Note: For panel D, the rates for 20-24 years in 2023 were 11,776.17 (11,114.36 to 12,531.50) for males and 5,528.19 (5,158.38 to 5,968.28).

**Appendix Figure 6. YLL rates per 100,000 population, level 2 NCDs causes, in MENA, in 2023, by age group and sex**


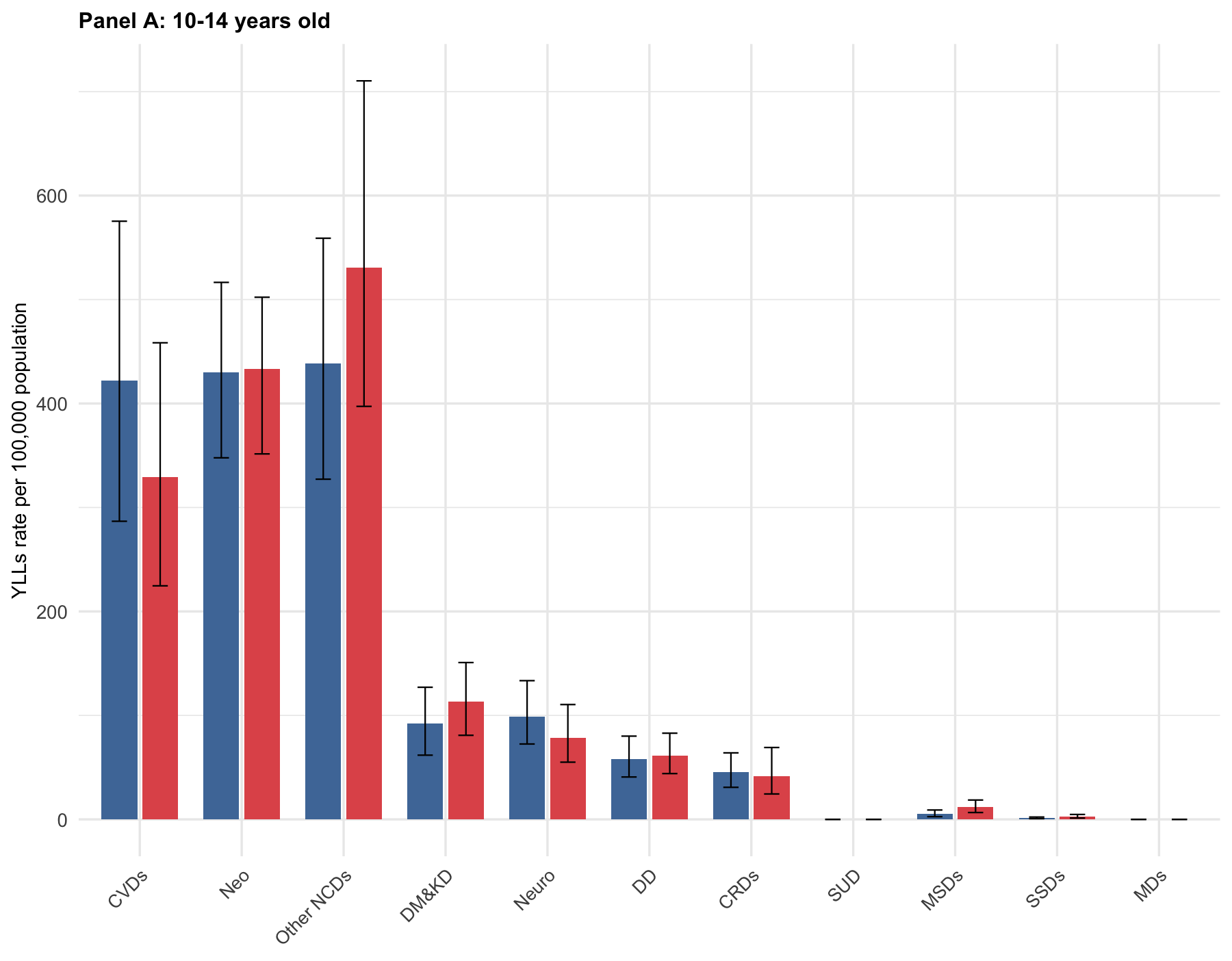


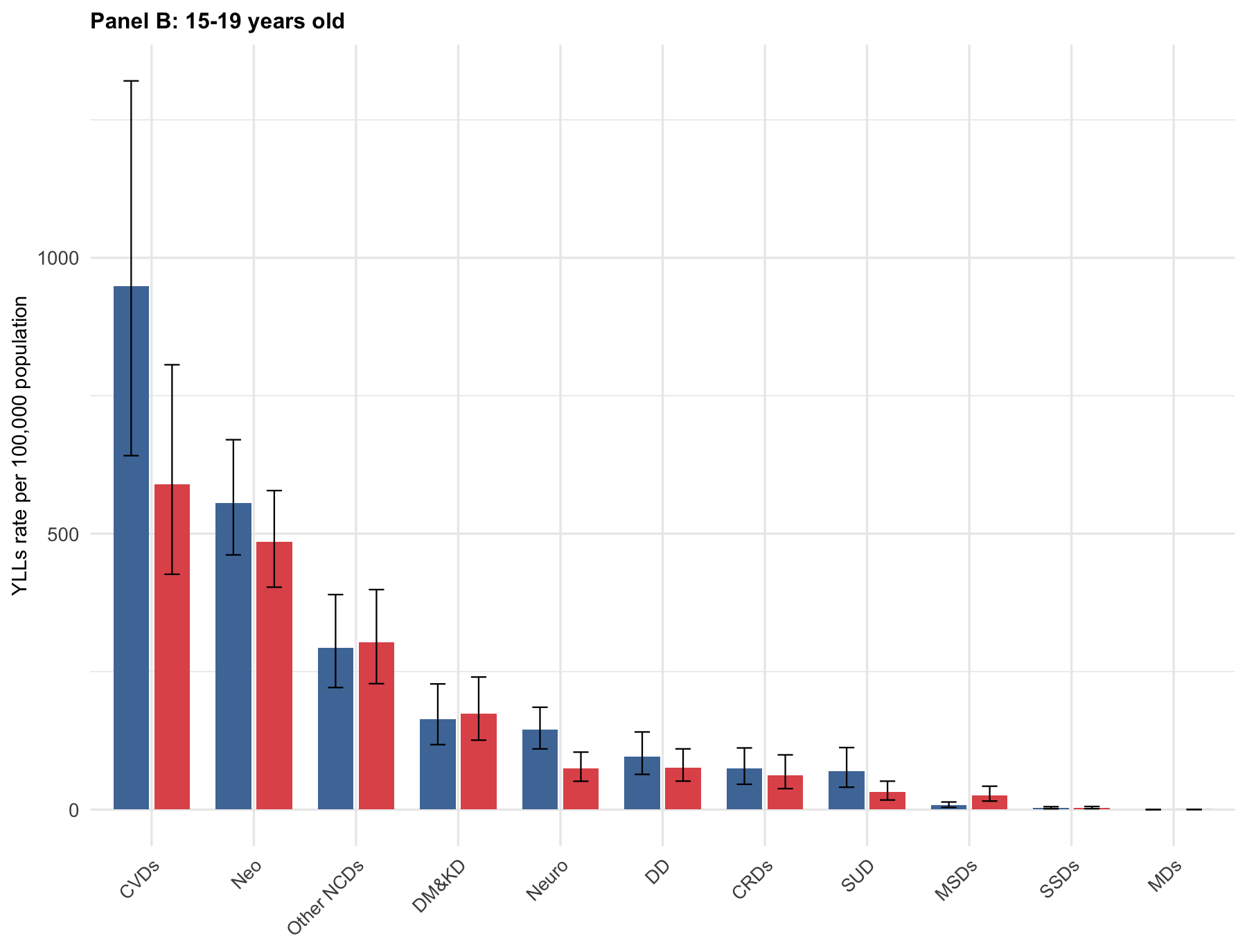


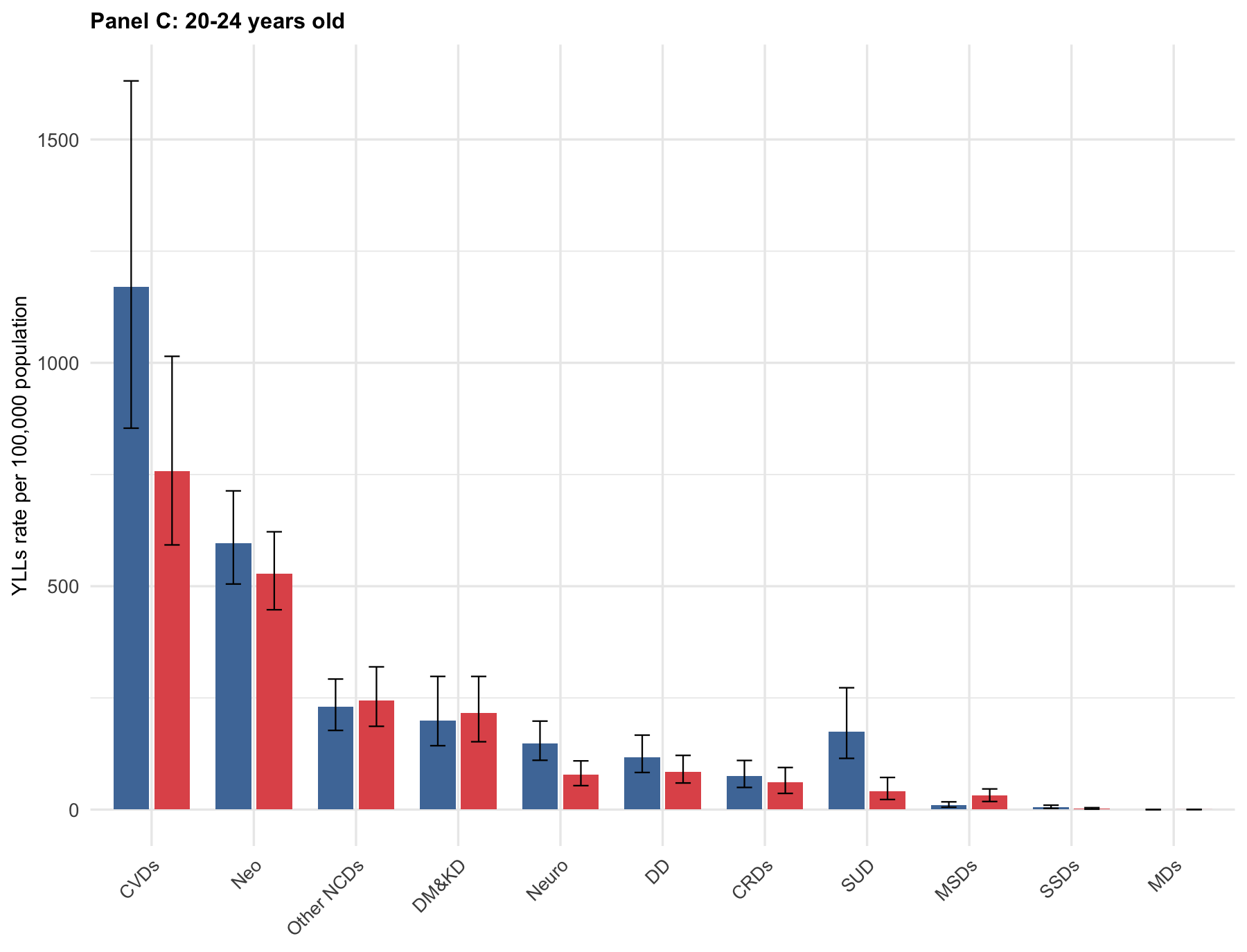


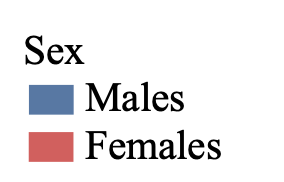


Abbreviations: CRDs=Chronic respiratory diseases; CVDs=cardiovascular diseases; DD=Digestive diseases; DM&KD=Diabetes and kidney diseases; MDs=Mental health disorders; MSDs=Musculoskeletal disorders; Neo=Neoplasms; Neuro= Neurological disorders; Other NCDs=Other non-communicable diseases; SSDs=Skin and subcutaneous diseases; SUD=Substance use disorders.

**Appendix Figure 7: YLL rates per 100,000 population, first 20 level 3 NCDs causes, in people 10-24 years old, in MENA, 2023, by sex**


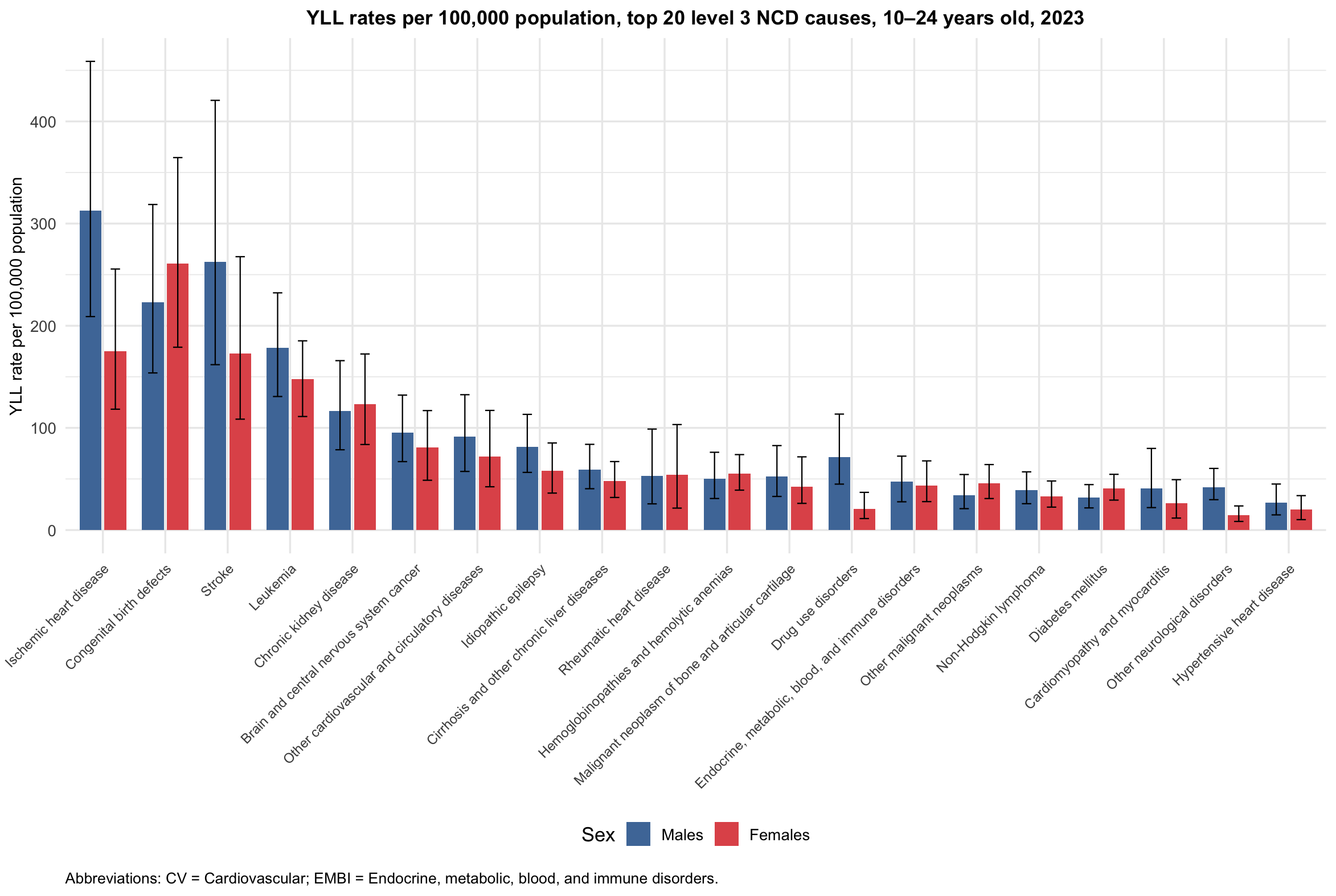


**Appendix Figure 8: YLL rates per 100,000 population (Panel A), and in percentage (Panel B), NCDs level 2, both sexes, 10-24 years old, 2023, by location**

**Panel A**


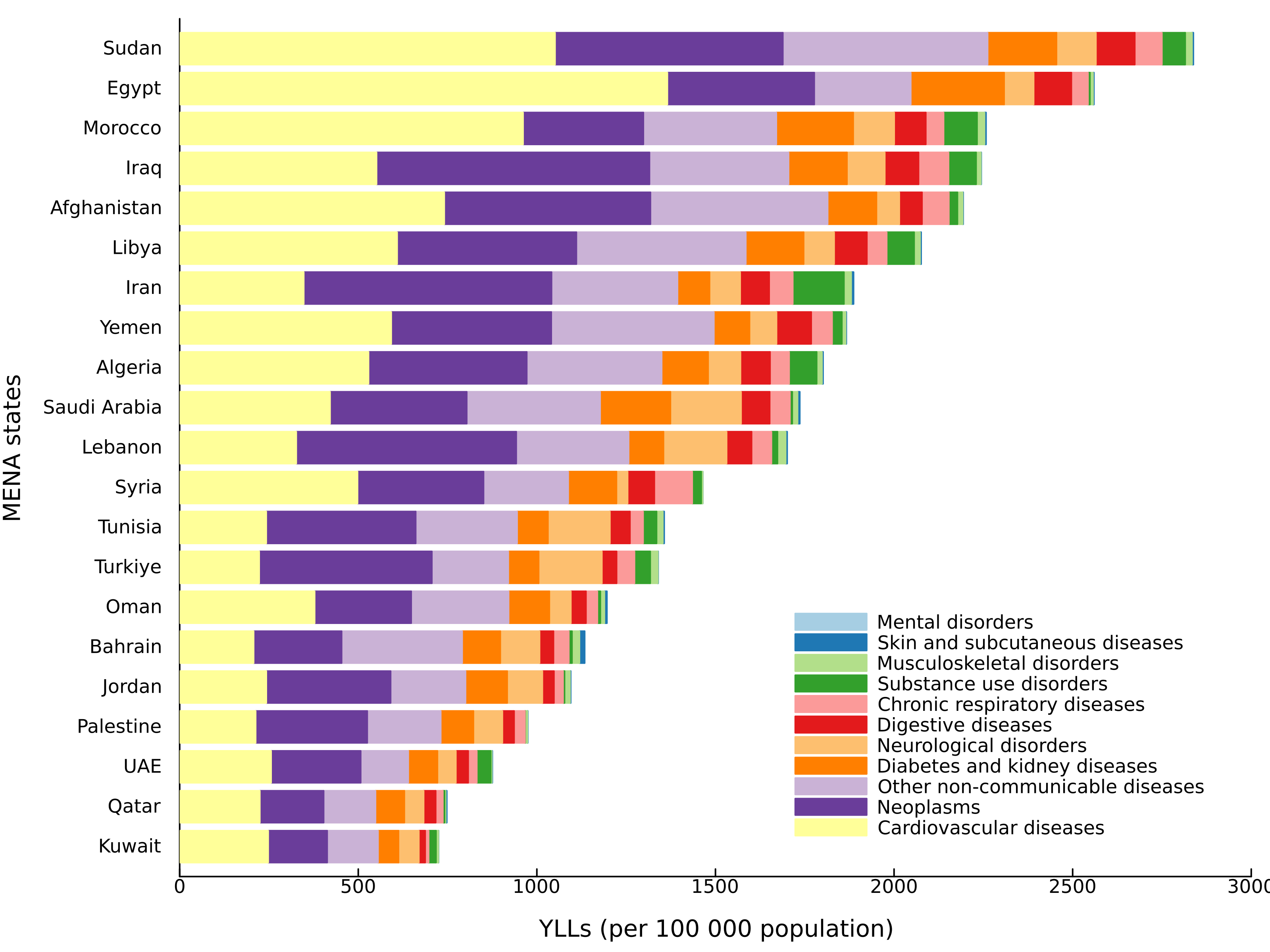


**Panel B**


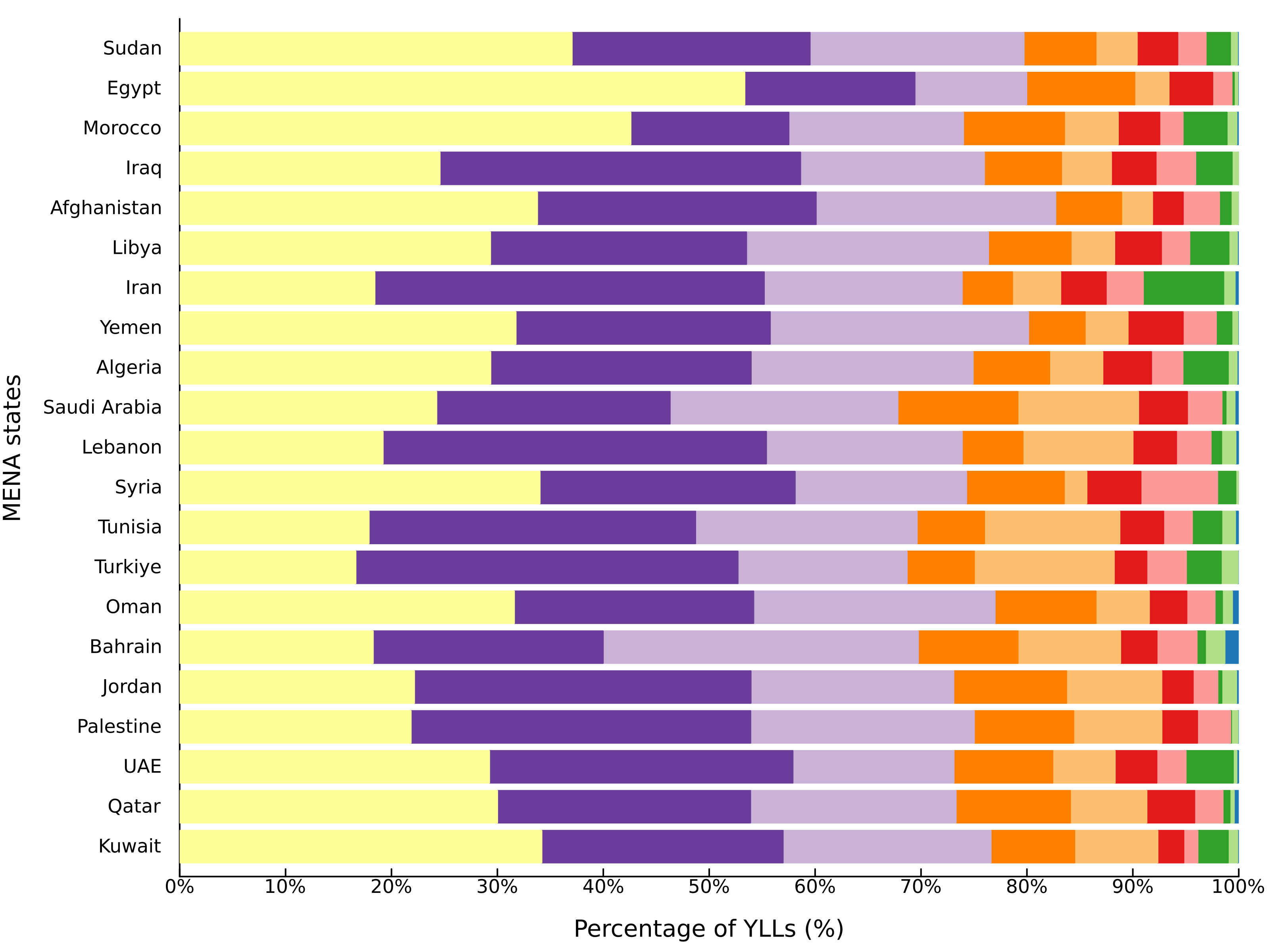


**Appendix Figure 9. Level 2 NCD causes, in MENA, in people aged 10-24 years, both sexes, from 1990 to 2023: YLLs rate per 100,000 population**

**
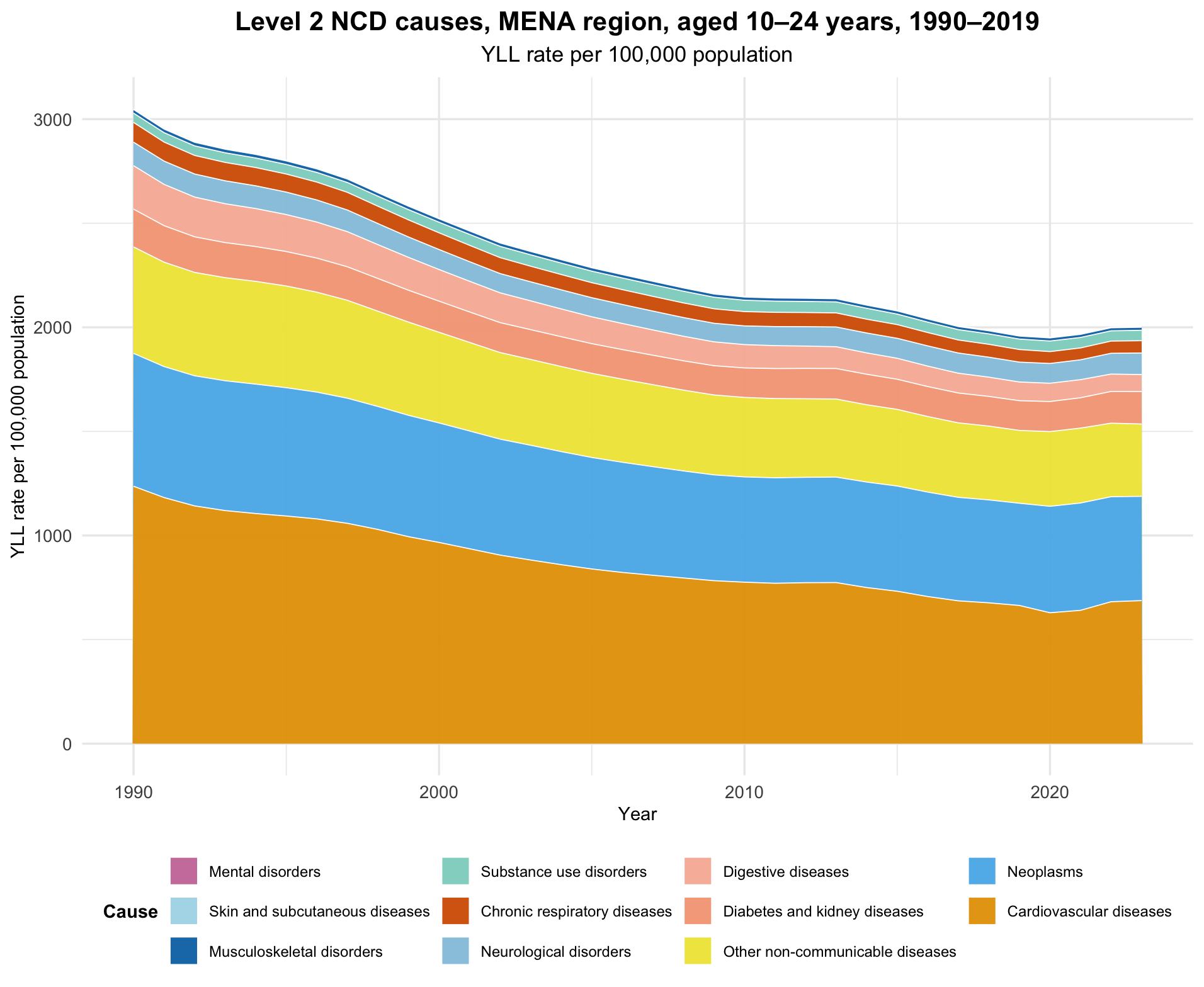
**

*Note: This aggregate cause contains the following Level 3 causes: congenital birth defects; urinary diseases; gynecological diseases; hemoglobinopathies and hemolytic anemias; endocrine, metabolic, blood, and immune disorders; oral disorders.

**Appendix Figure 10. All-cause YLD rates per 100,000 population, in people aged 10–24 years from 1990 to 2023**

**by sex and age group**


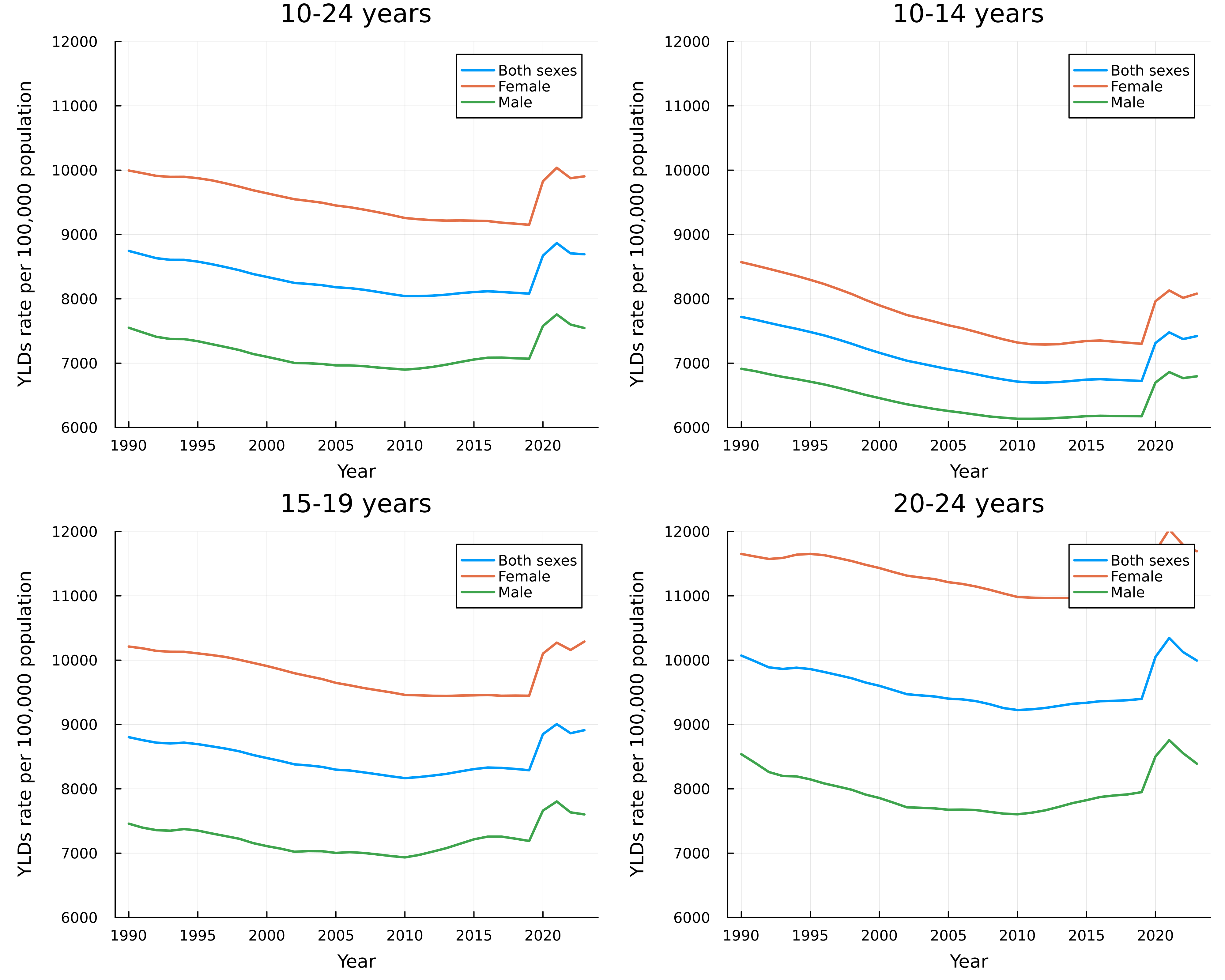


**Appendix Figure 11. YLD rates per 100,000 population, level 2 NCDs causes, MENA Region, 2023, by age**

**group and sex**

**
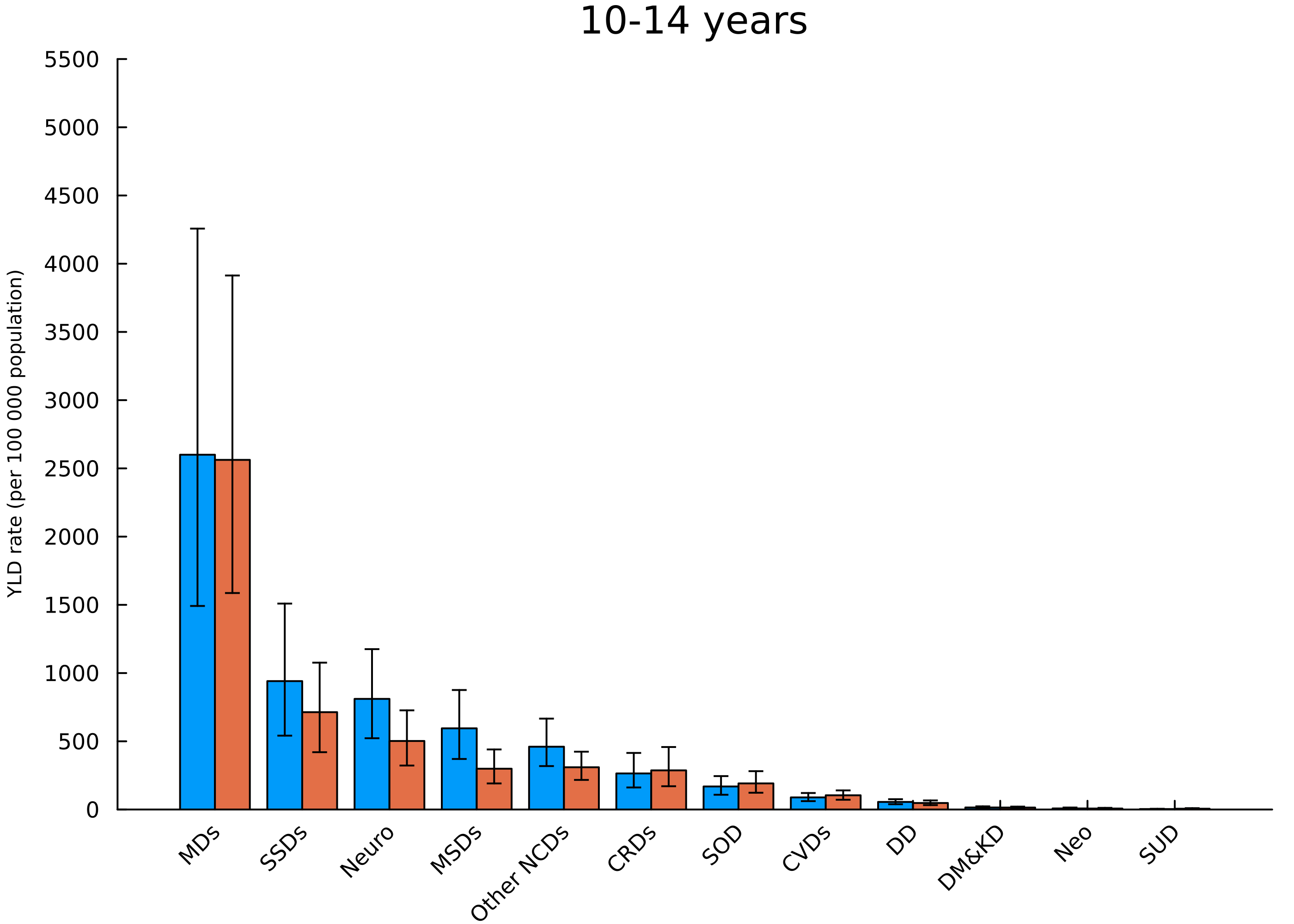
**

**
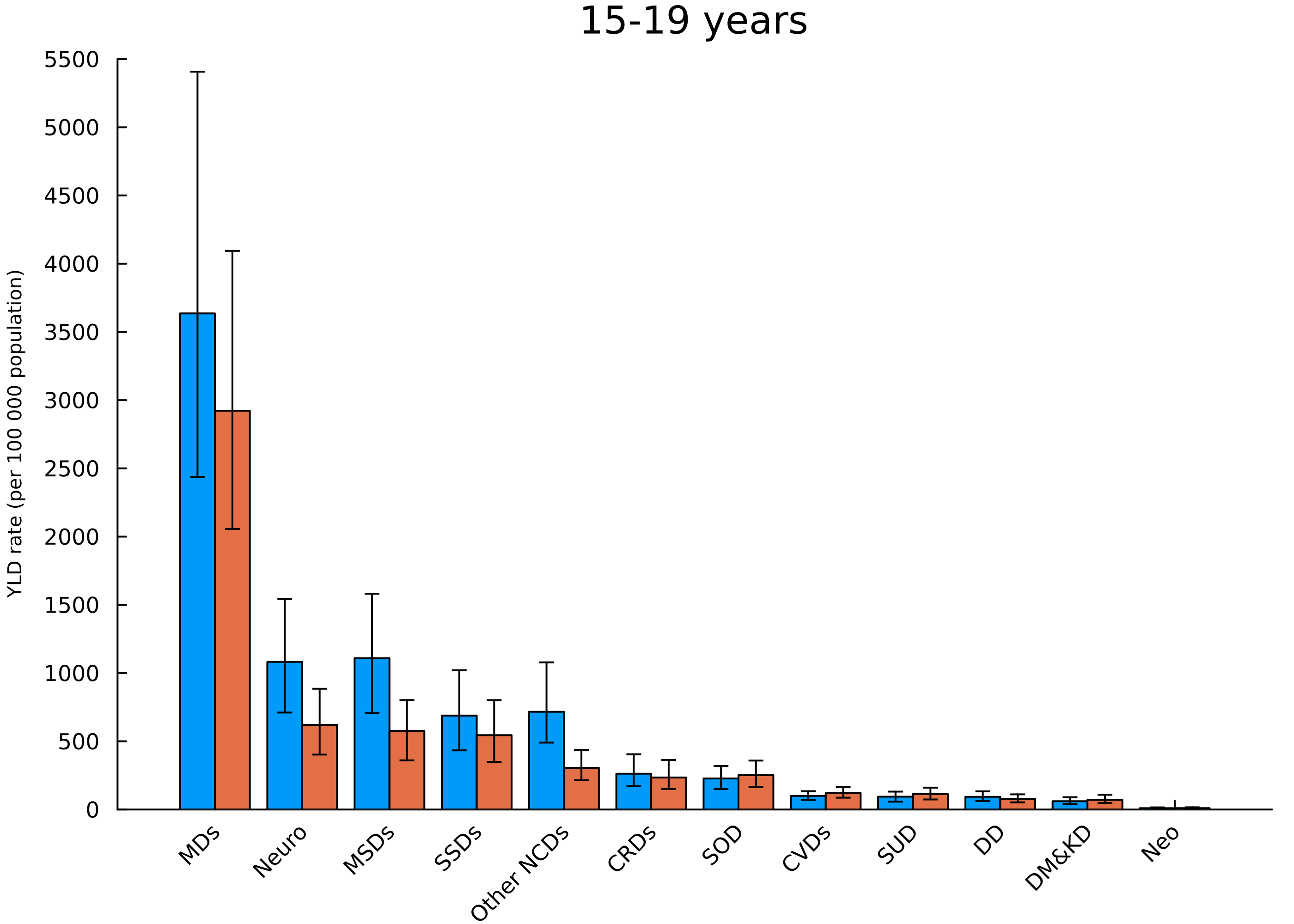
**


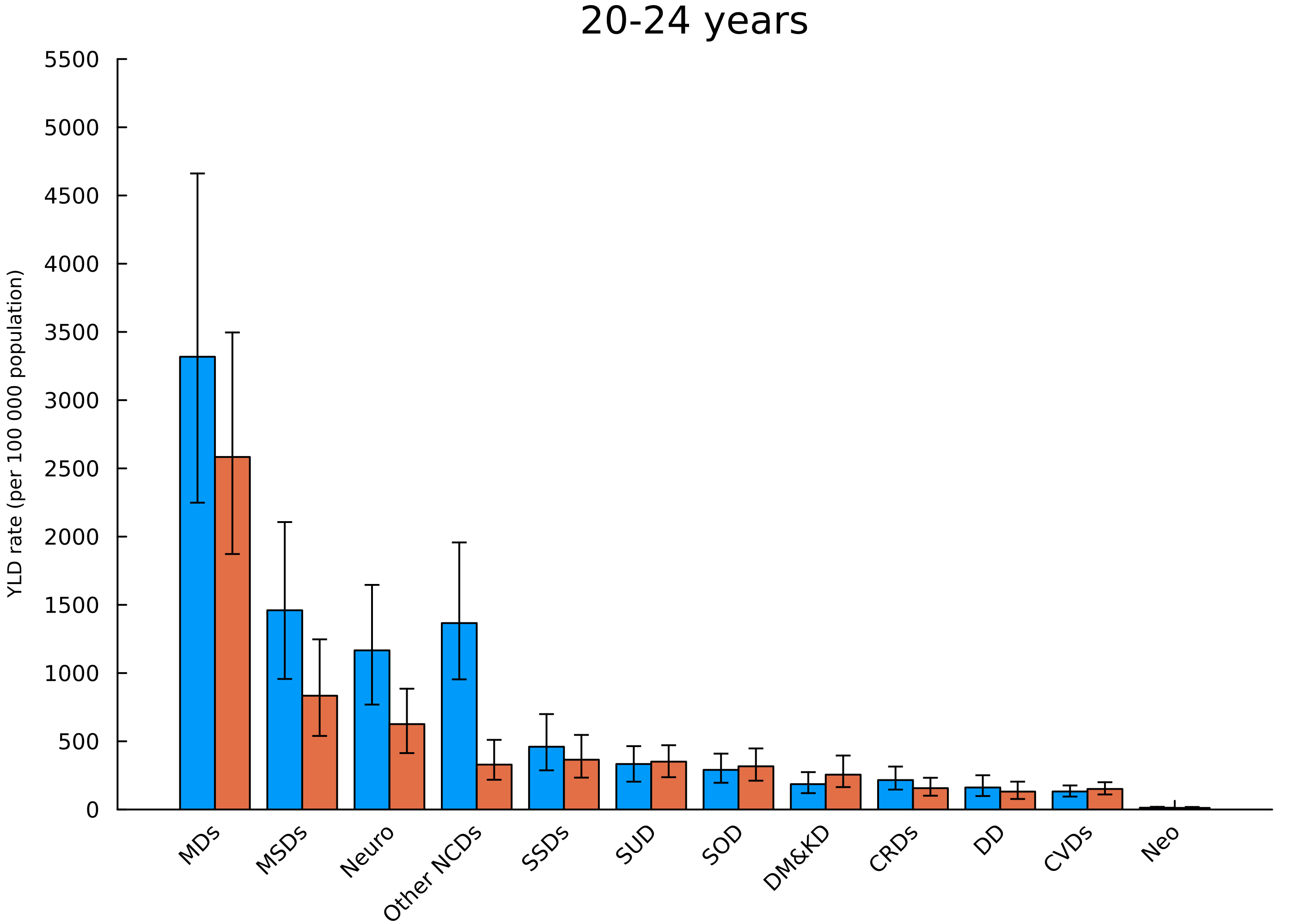


Abbreviations: CRDs=Chronic respiratory diseases; CVDs=cardiovascular diseases; DD=Digestive diseases; DM&KD=Diabetes and kidney diseases; MDs=Mental health disorders; MSDs=Musculoskeletal disorders; Neo=Neoplasms; Neuro= Neurological disorders; Other NCDs=Other non-communicable diseases; SSDs=Skin and subcutaneous diseases; SUD=Substance use disorders; SOD= Sense organ diseases.

**Appendix Figure 12. YLD rates per 100,000 population, first 20 level 3 NCDs causes, in people 10-24 years old,**

**in the MENA Region, 2023, by sex**

**
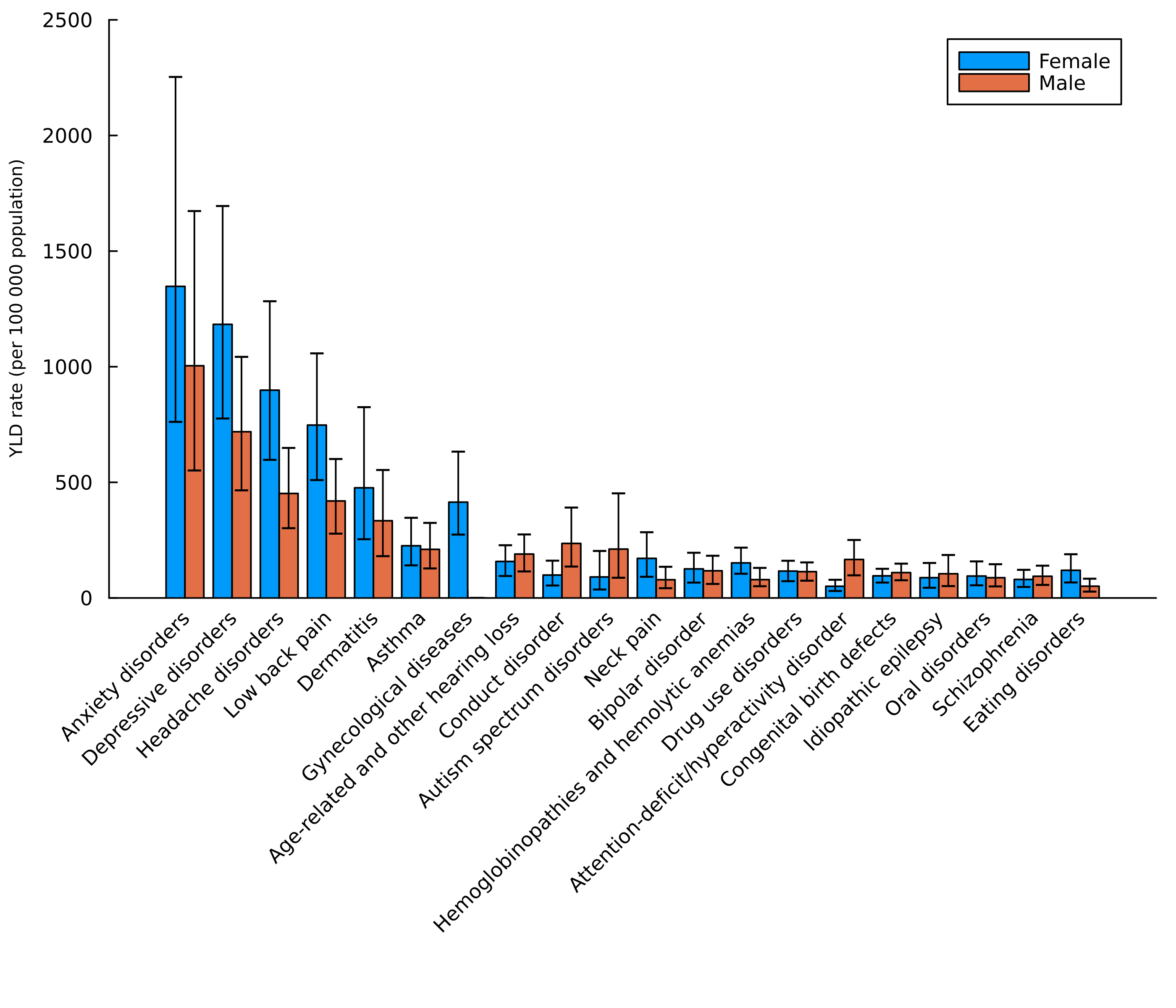
**

**Appendix Figure 13. YLD rates per 100,000 population (Panel A), and in percentage (Panel B), NCDs level 2,**

**both sexes, 10-24 years old, 2023, by location**

**Panel A**

**
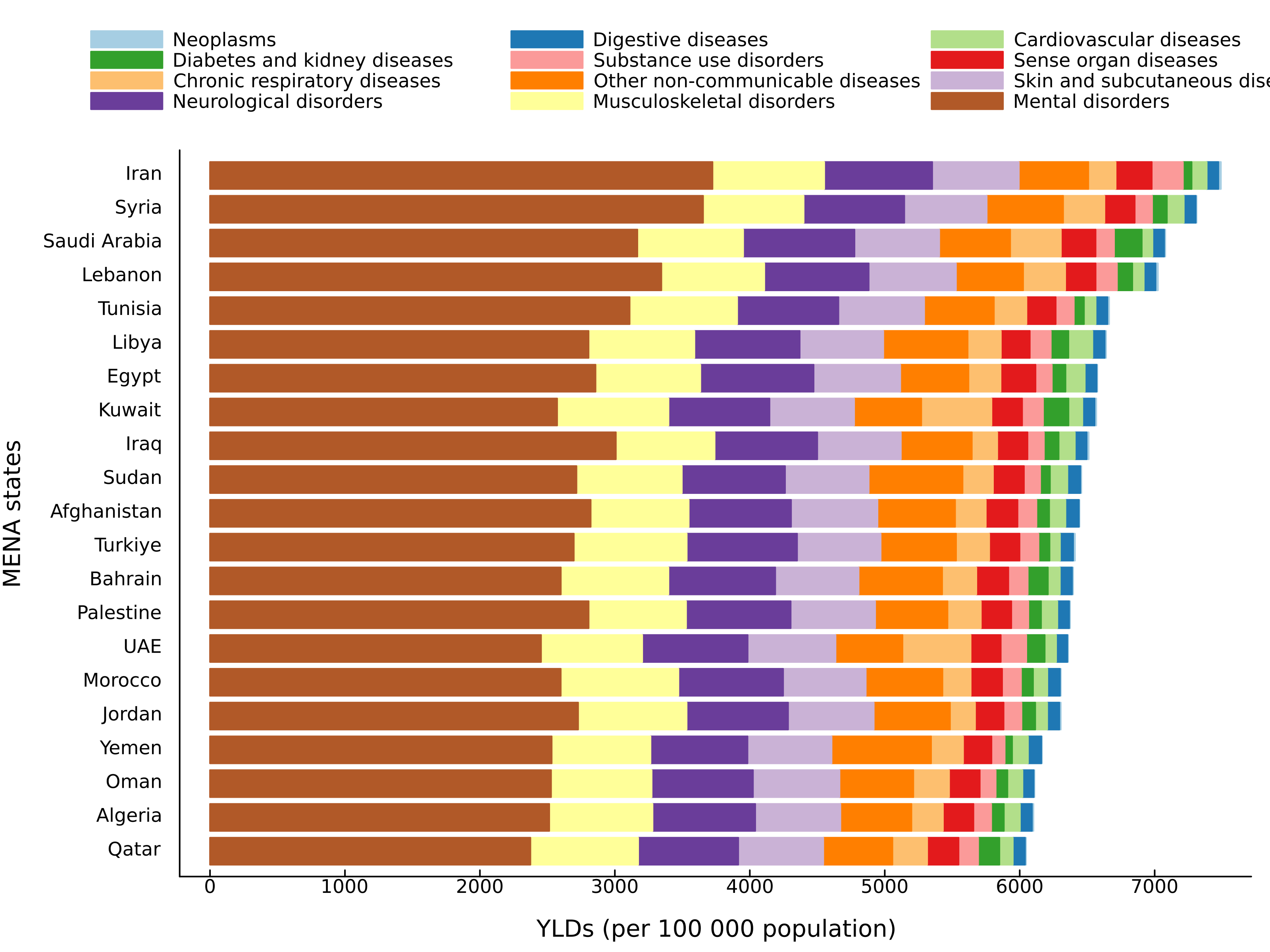
**

**Panel B**

**
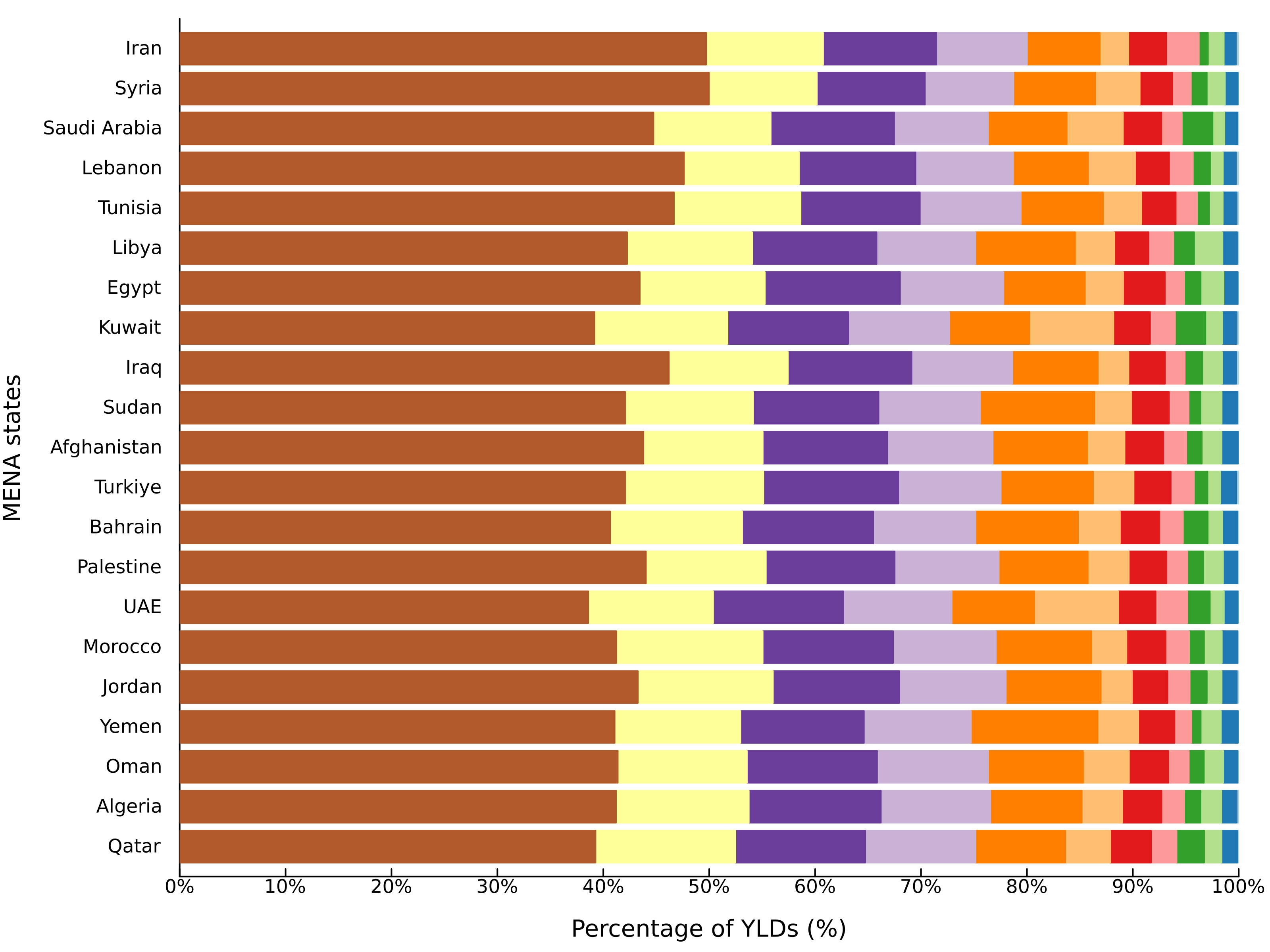
**

**Appendix Figure 14. Level 2 NCD causes, in MENA, in people aged 10-24 years, both sexes, from 1990 to 2023: YLDs rate per 100,000 population**

**
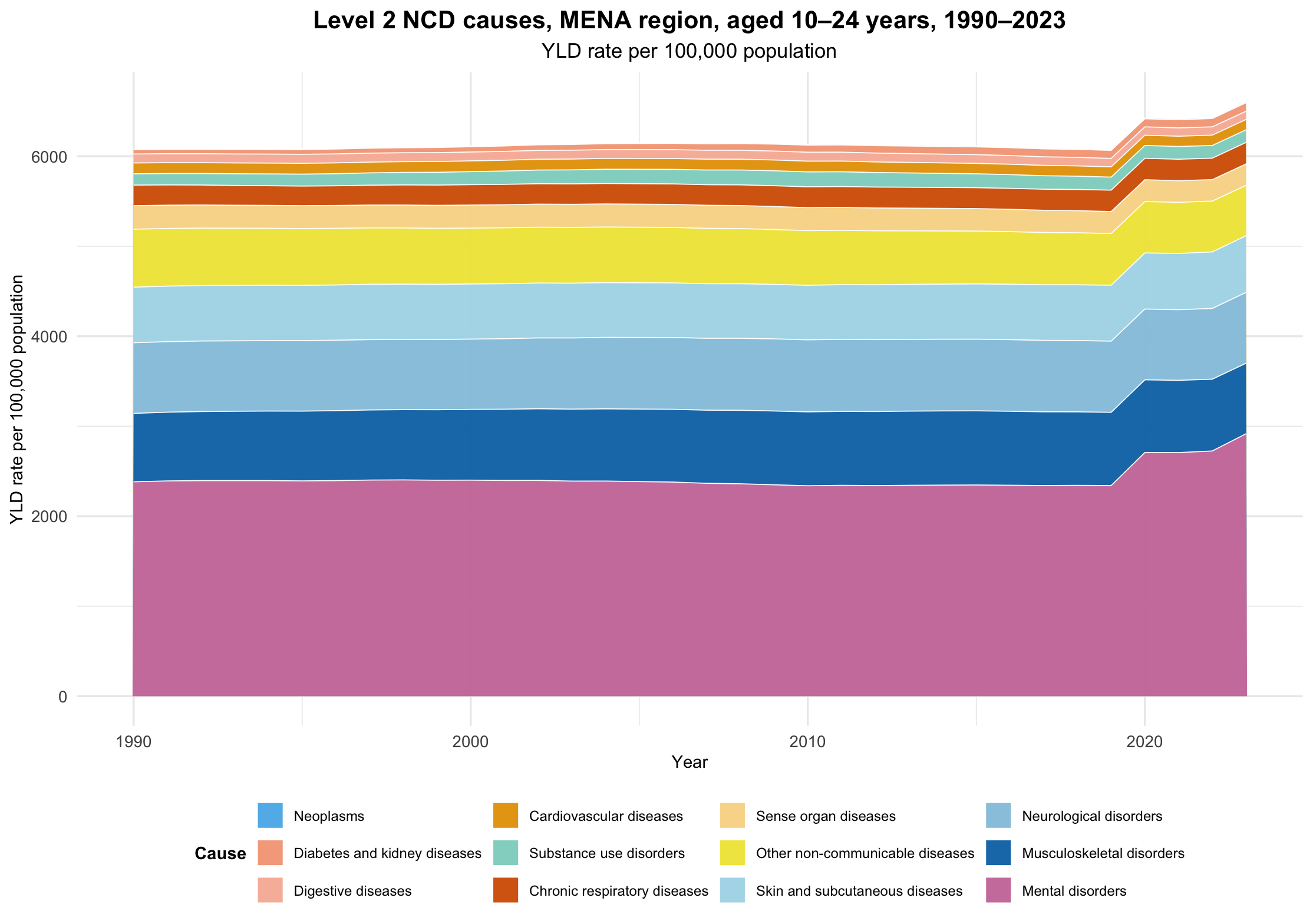
**

*Note: This aggregate cause contains the following Level 3 causes: congenital birth defects; urinary diseases; gynecological diseases; hemoglobinopathies and hemolytic anemias; endocrine, metabolic, blood, and immune disorders; oral disorders.

**Appendix figure 15A. All-cause DALY rates per 100,000 population, in people aged 10–24 years in MENA from 1990 to**

**2023 by sex and age group**

**
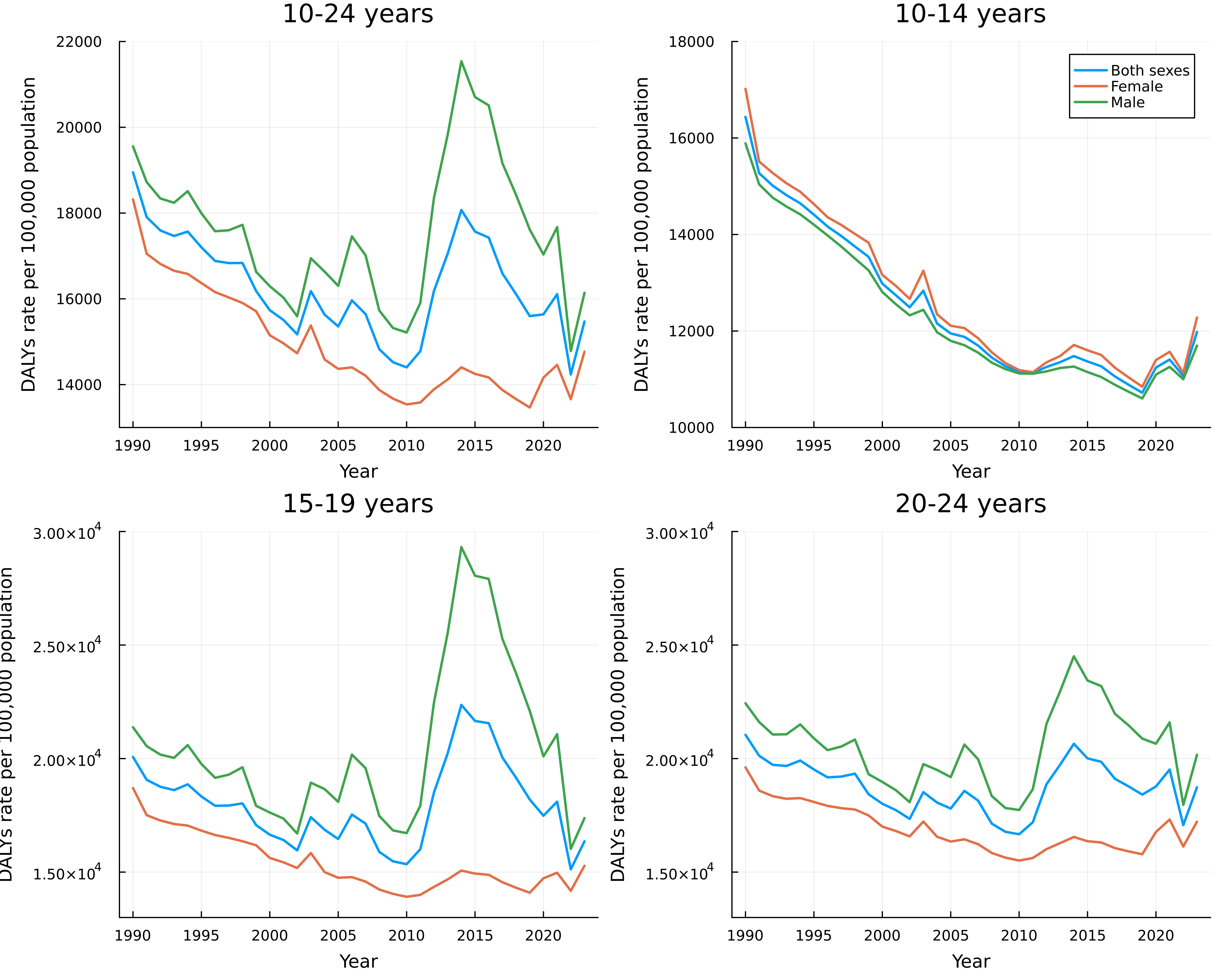
**

**Appendix Figure 15B. DALY rates per 100,000 population due to Level 1 Cause, in people aged 10–24 years, in MENA region from 1990 to 2023**

**
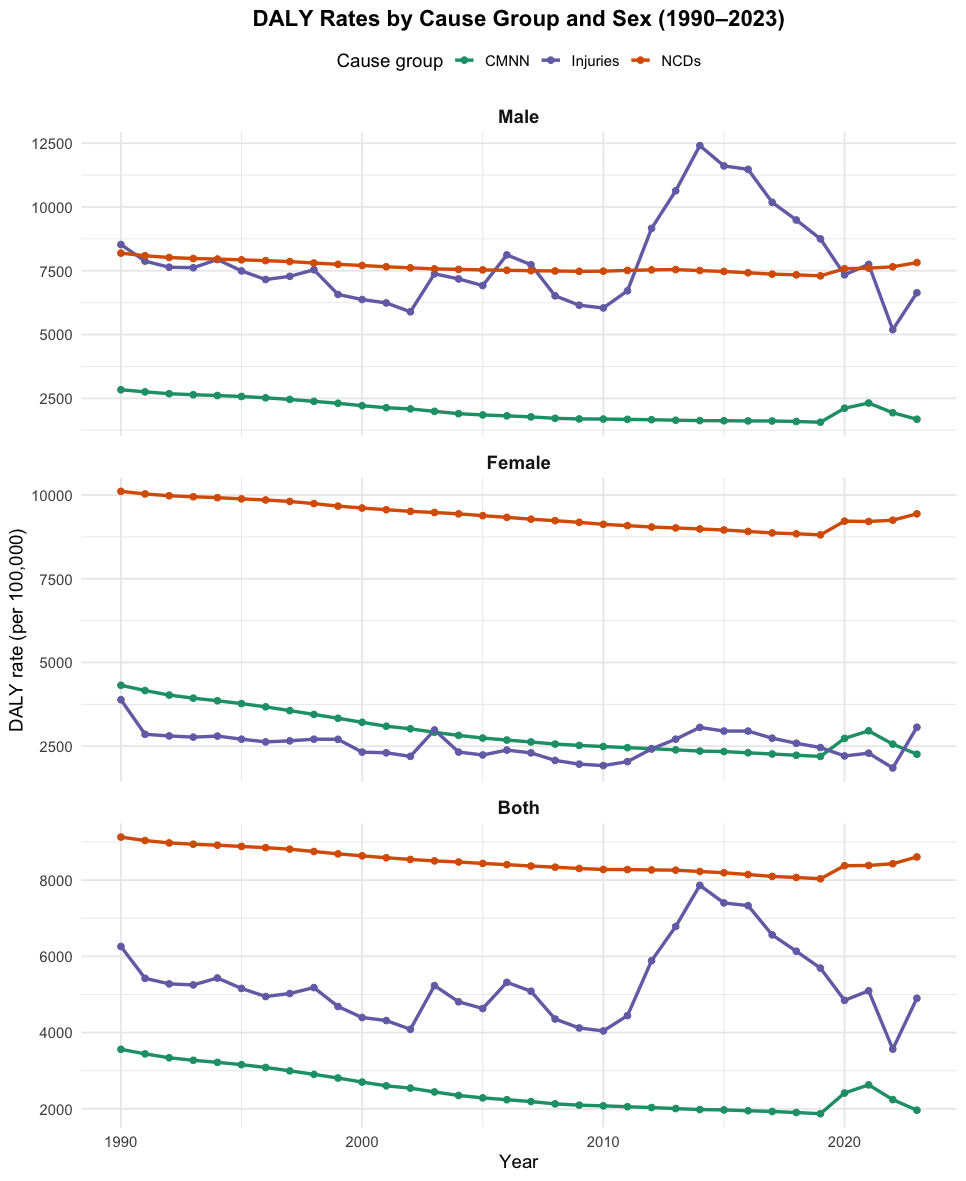
**

**Appendix Figure 16. DALY rates per 100,000 population, level 2 NCDs causes, MENA, 2023, by age**

**group and sex**


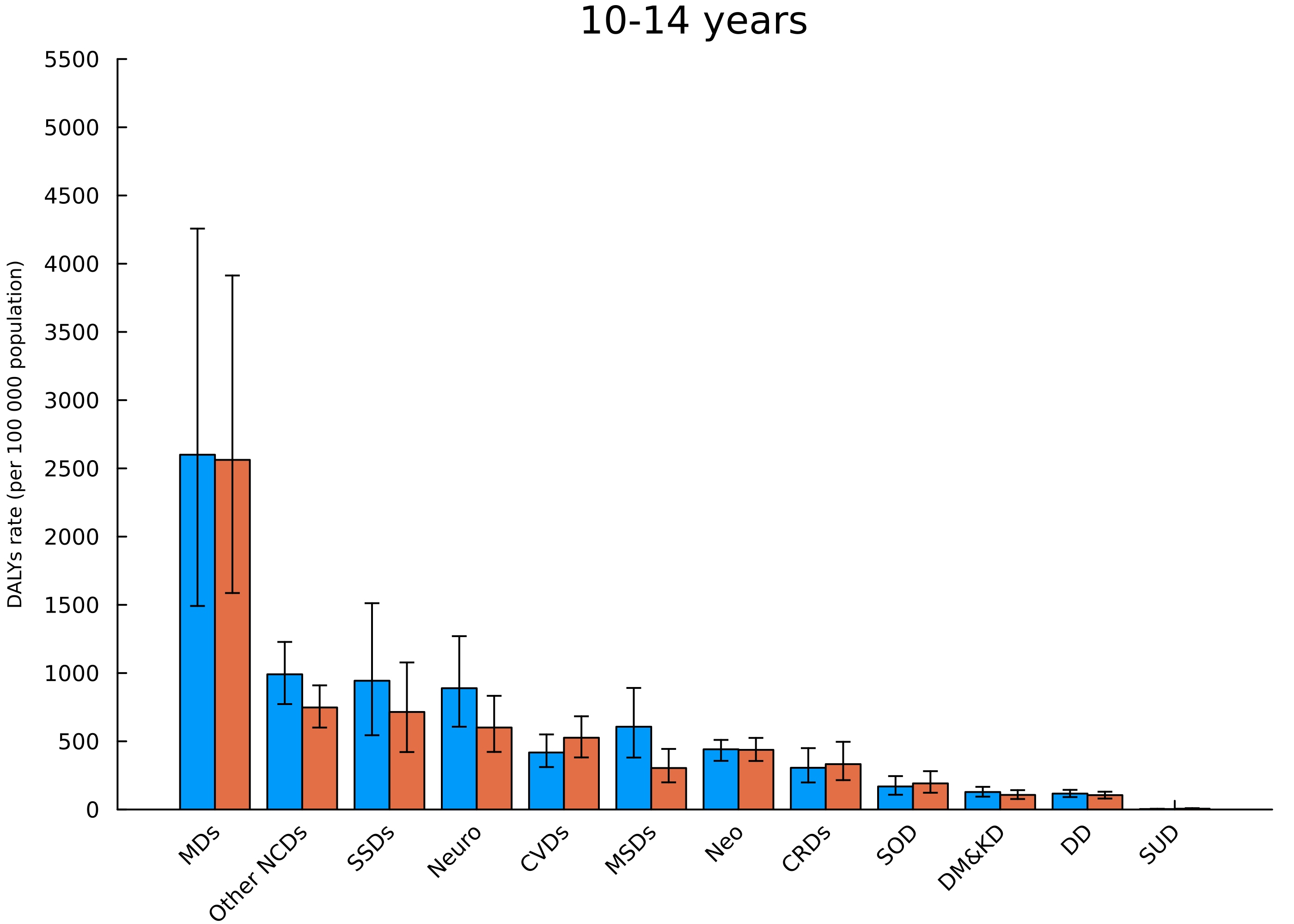


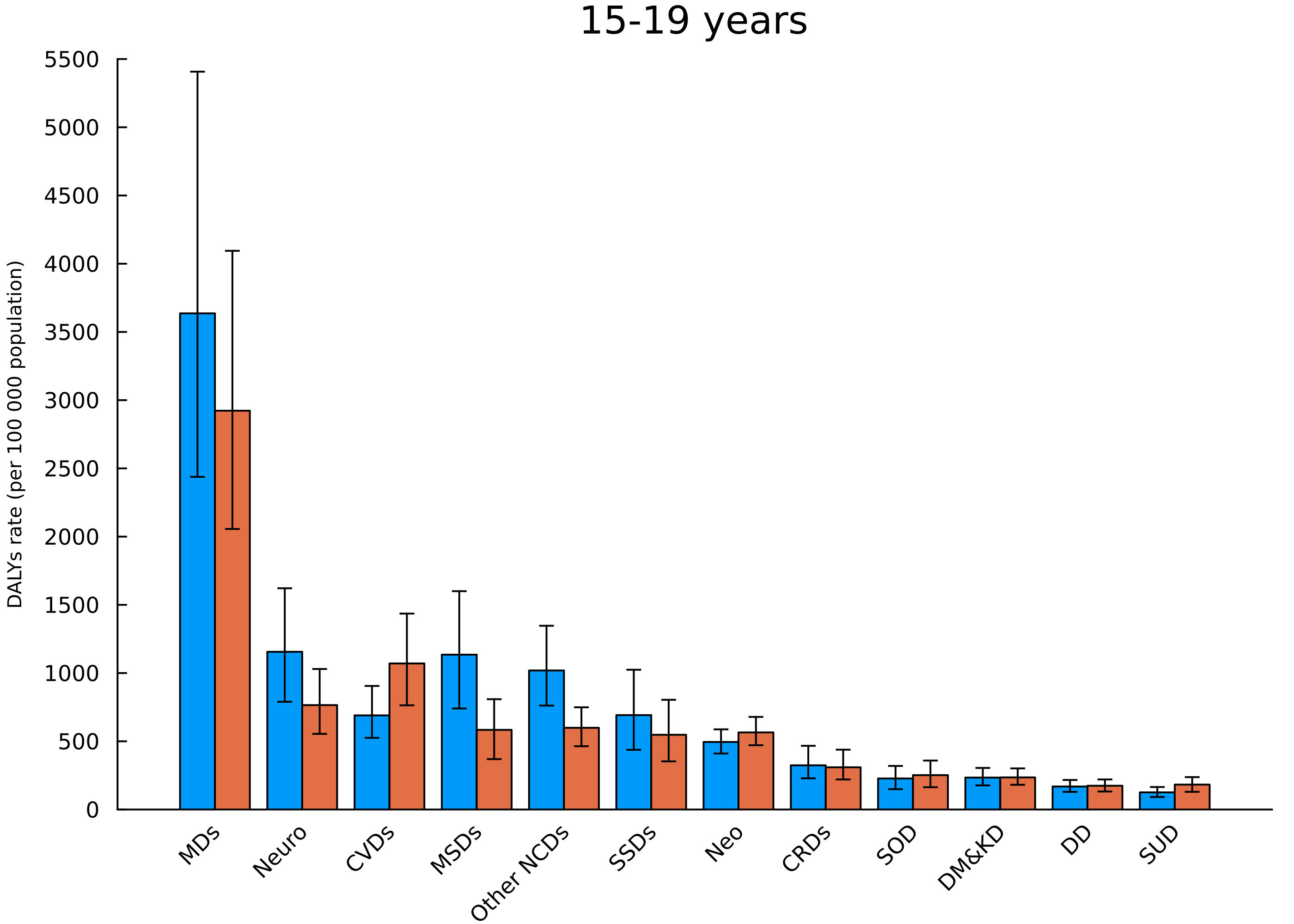


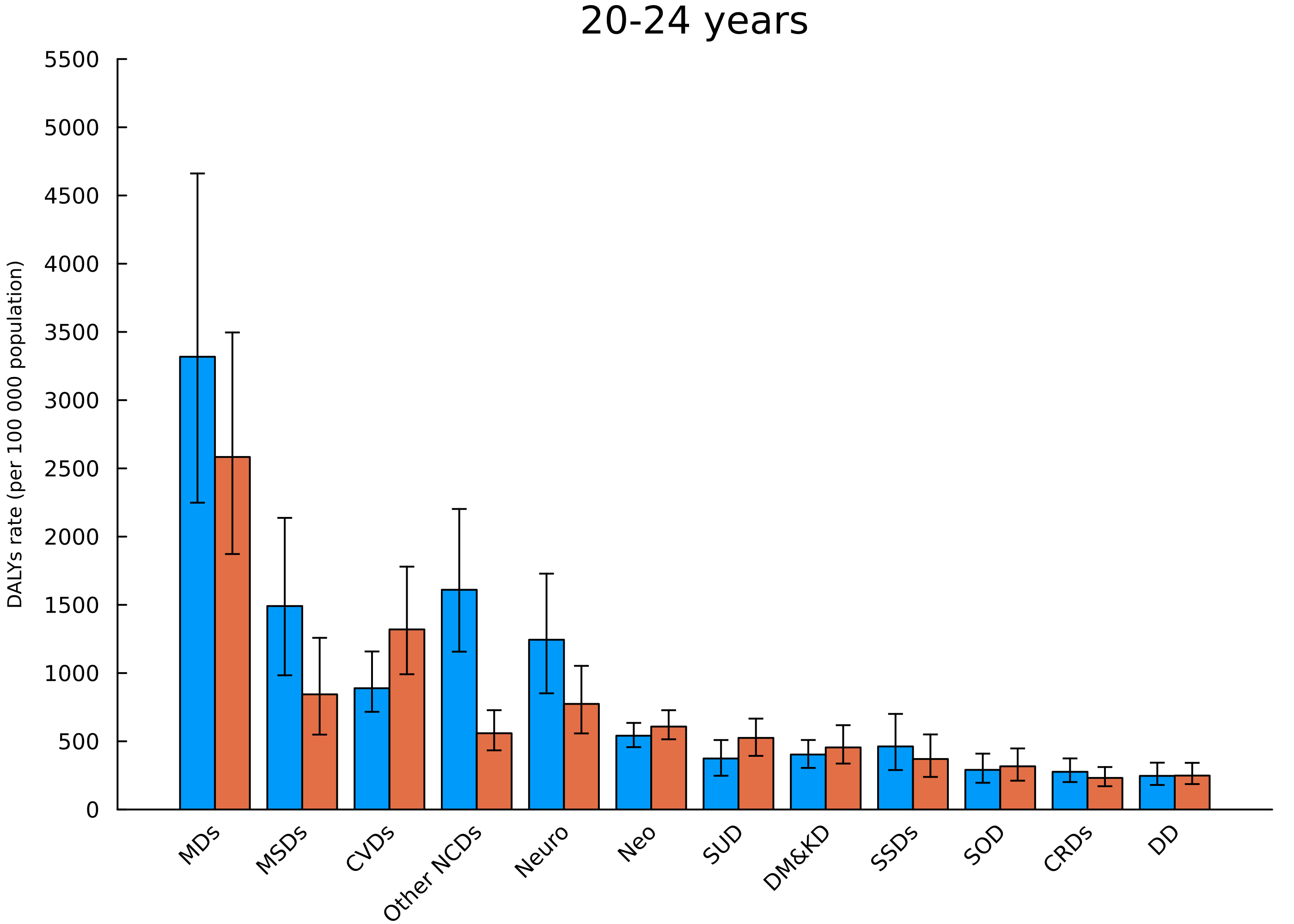


Abbreviations: CRDs=Chronic respiratory diseases; CVDs=cardiovascular diseases; DD=Digestive diseases; DM&KD=Diabetes and kidney diseases; MDs=Mental health disorders; MSDs=Musculoskeletal disorders; Neo=Neoplasms; Neuro= Neurological disorders; Other NCDs=Other non-communicable diseases; SSDs=Skin and subcutaneous diseases; SUD=Substance use disorders; SOD= Sense organ diseases.

**Appendix Figure 17. DALY rates per 100,000 population, first 20 level 3 NCDs causes, in people 10-24 years old,**

**in MENA, 2023, by sex**

**
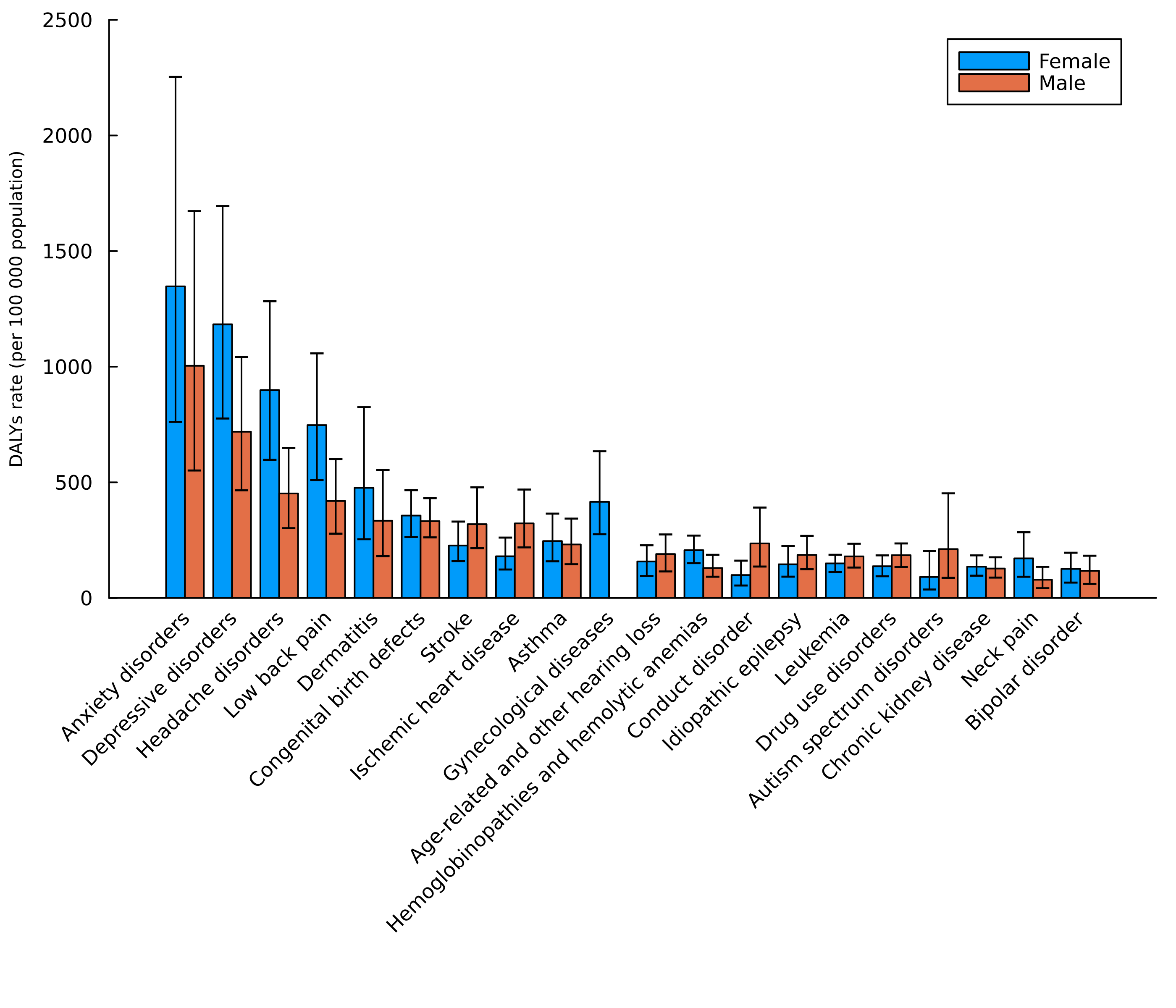
**

**Appendix Figure 18. DALY rates per 100,000 population, among 10-24 years old adolescents, MENA, both sexes, 2023,**

**level 2 NCDs**

**Cardiovascular diseases**
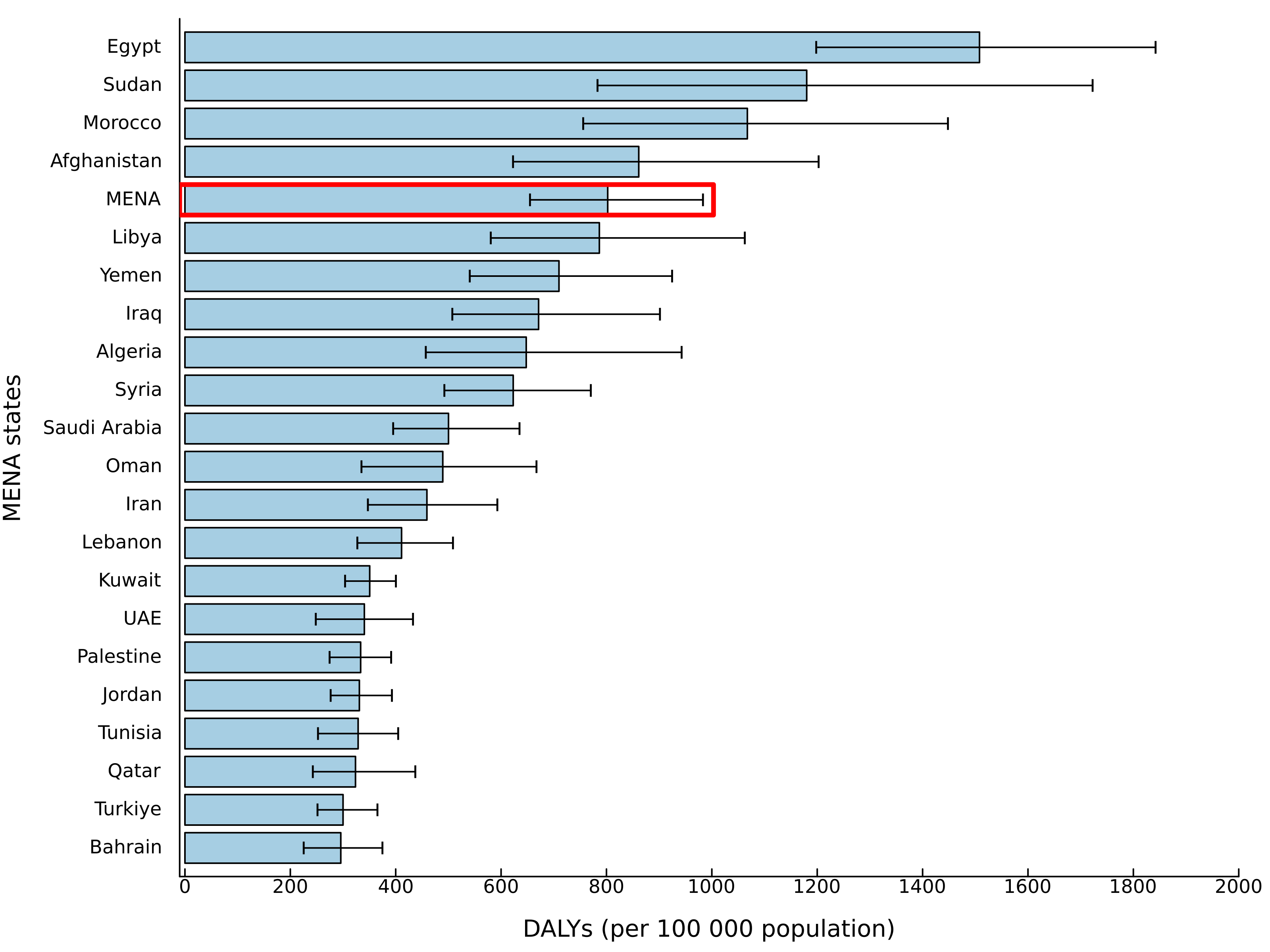


**Digestive diseases**


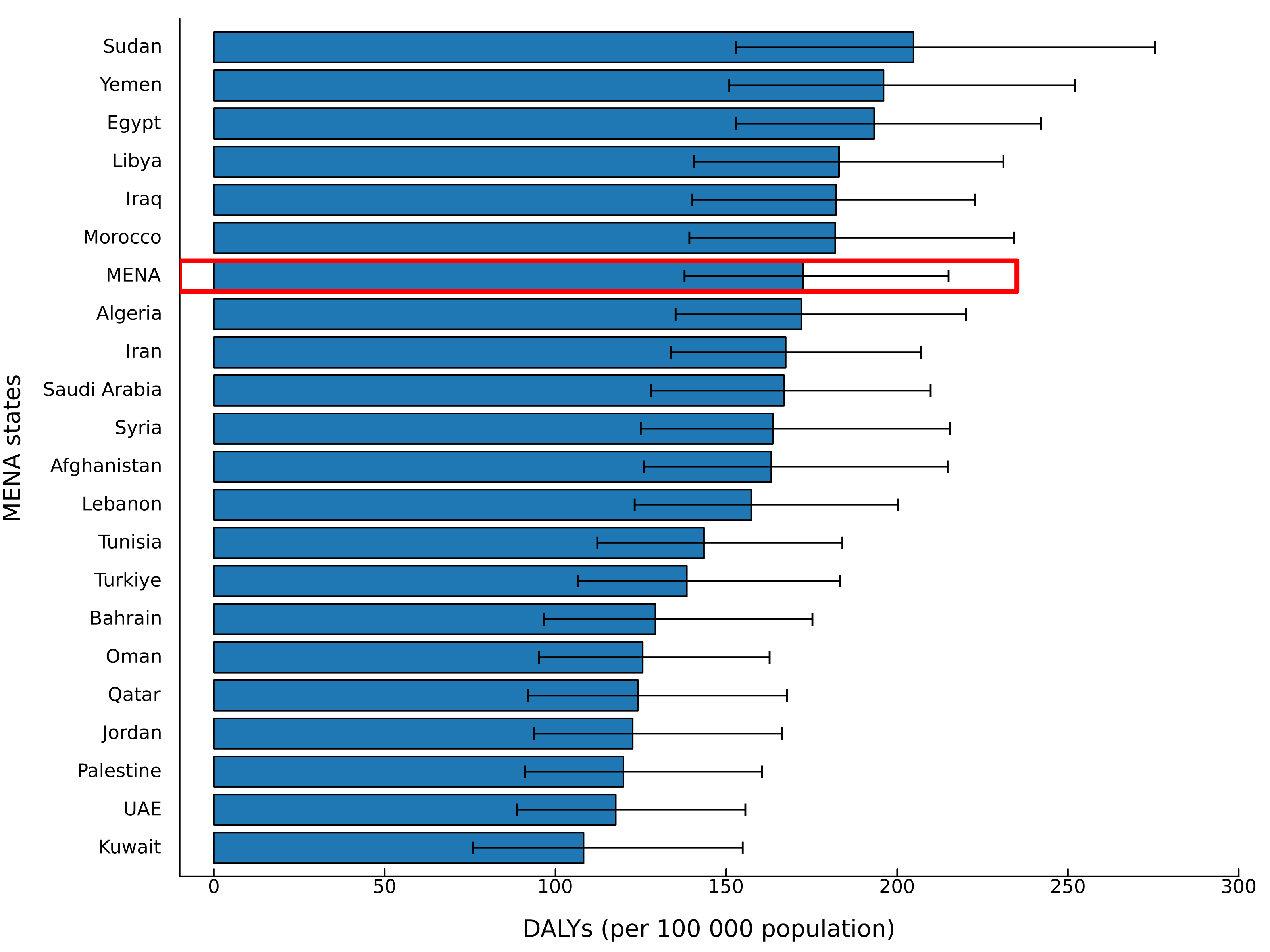


**Neoplasms**

**
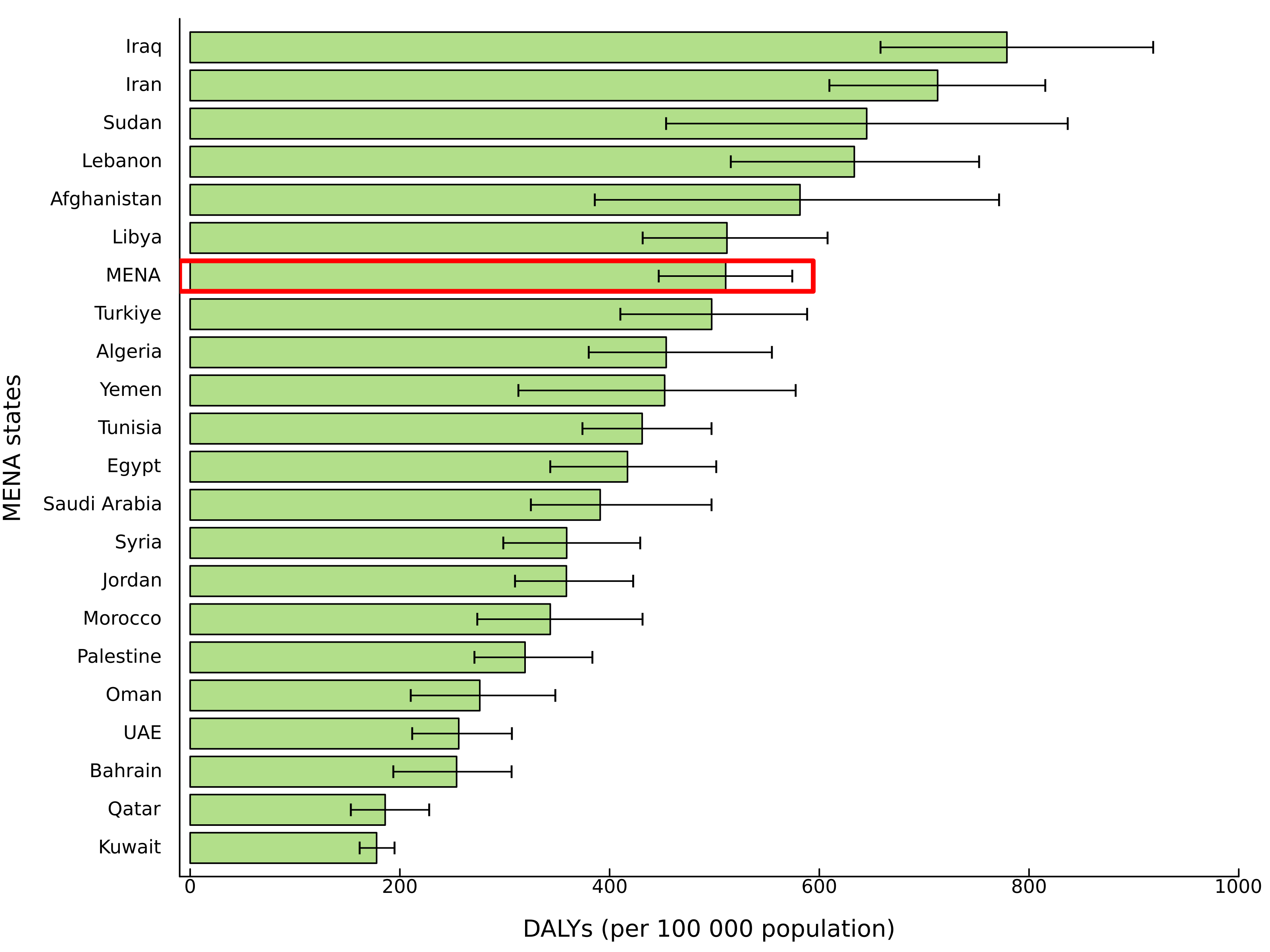
**

**Substance use disorders**

**
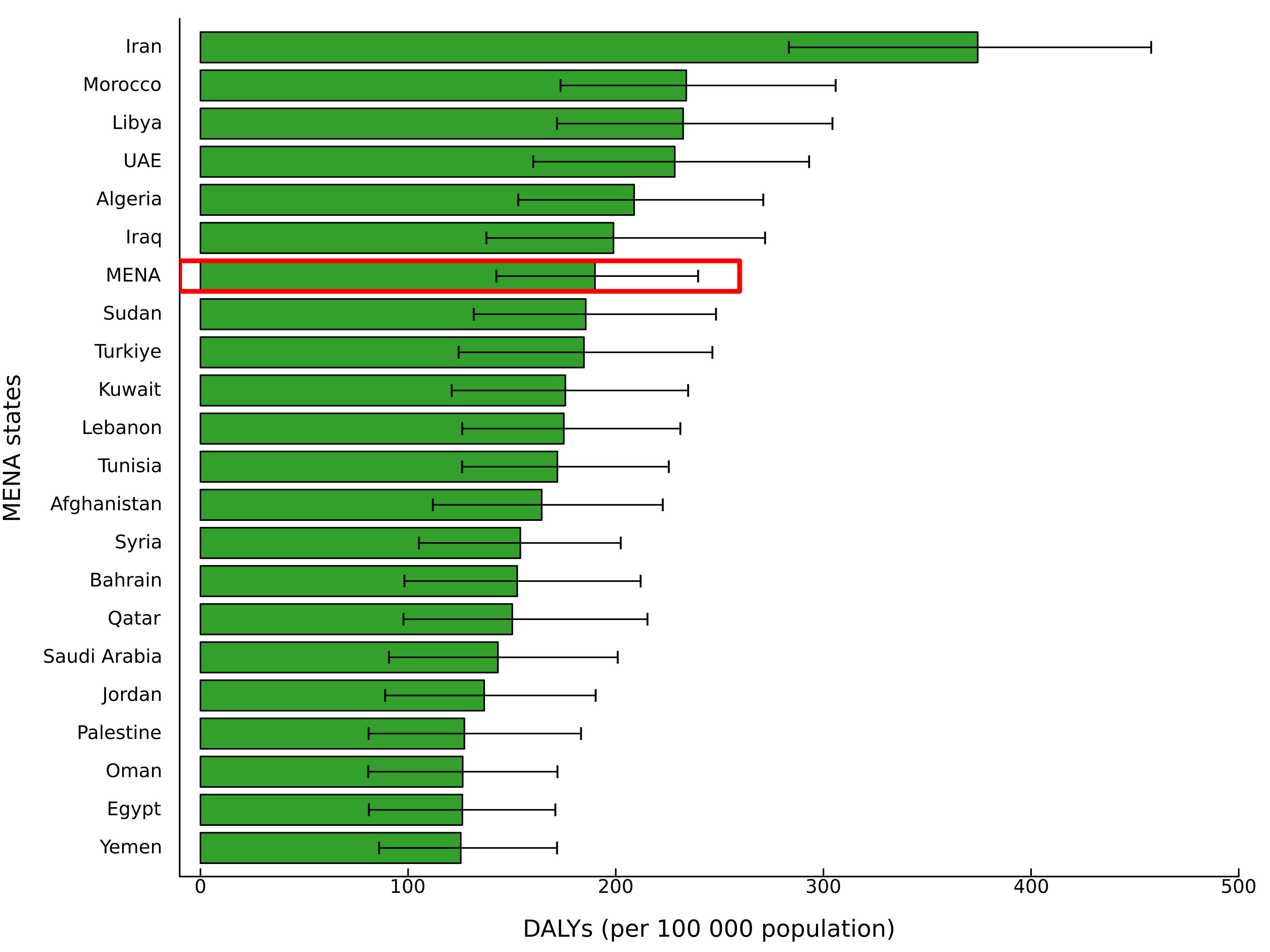
**

**Diabetes and kidney diseases

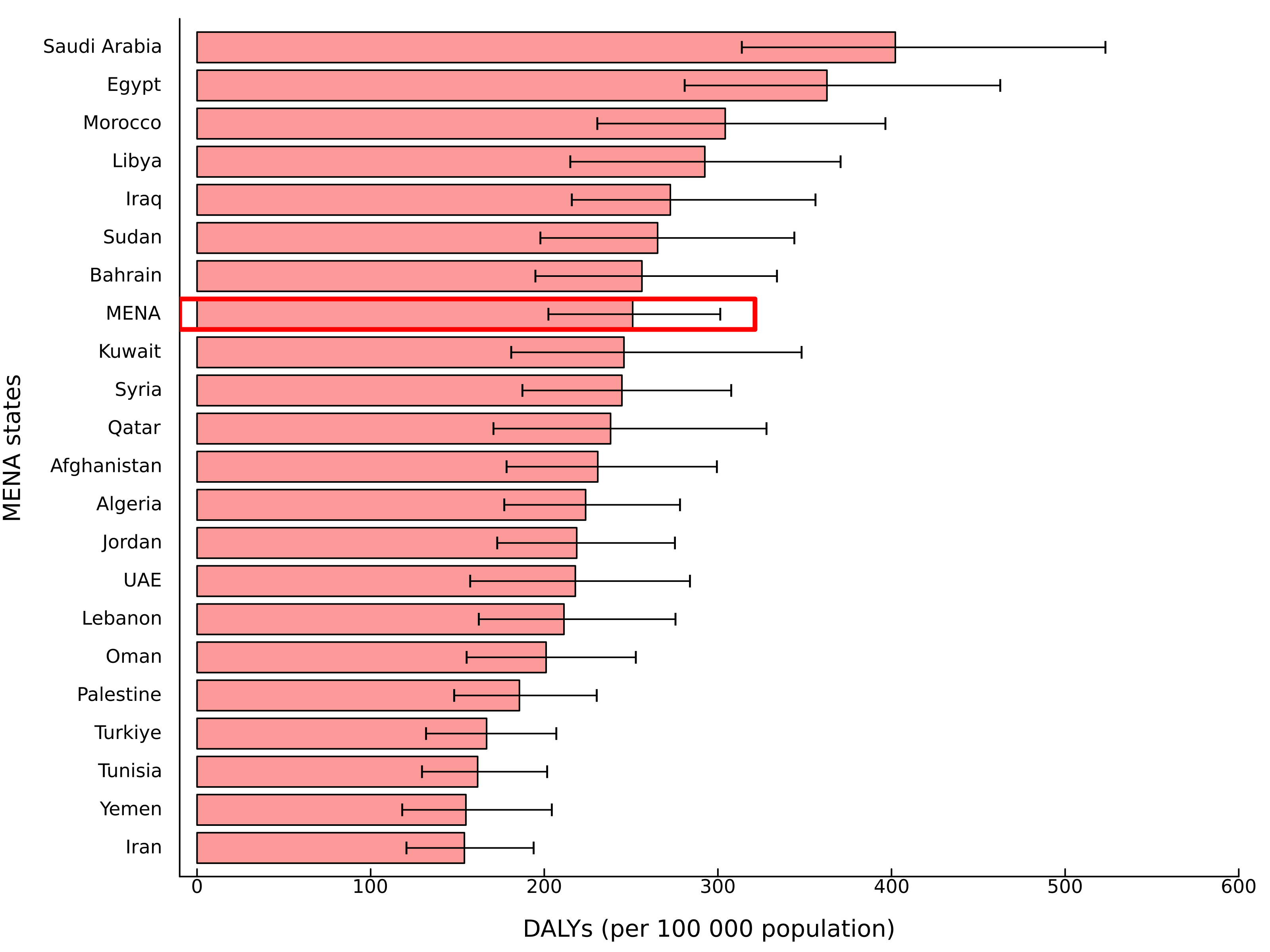
**

**Appendix Figure 19. DALY rate per 100,000 population, NCD Level 2 causes, in MENA, 10-24 years, both sexes, from 1990 to 2023:**

**
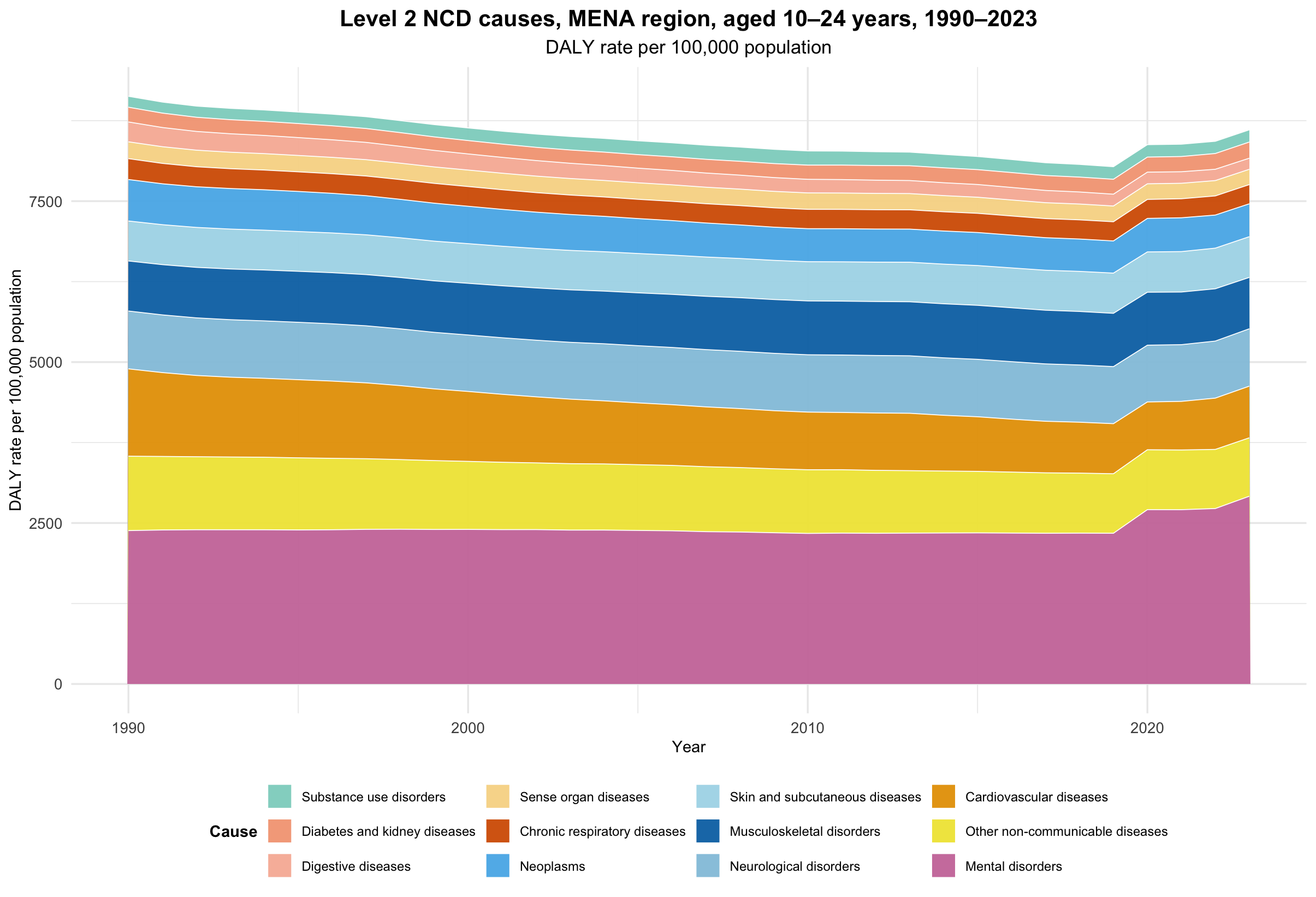
**

*Note: This aggregate cause contains the following Level 3 causes: congenital birth defects; urinary diseases; gynaecological diseases; haemoglobinopathies and haemolytic anaemias; endocrine, metabolic, blood, and immune disorders; oral disorders.

**Appendix Figure 20. YLL and YLD rates per 100,000 among 10-24 years old for selected countries 1990-2023**

**A.**

**
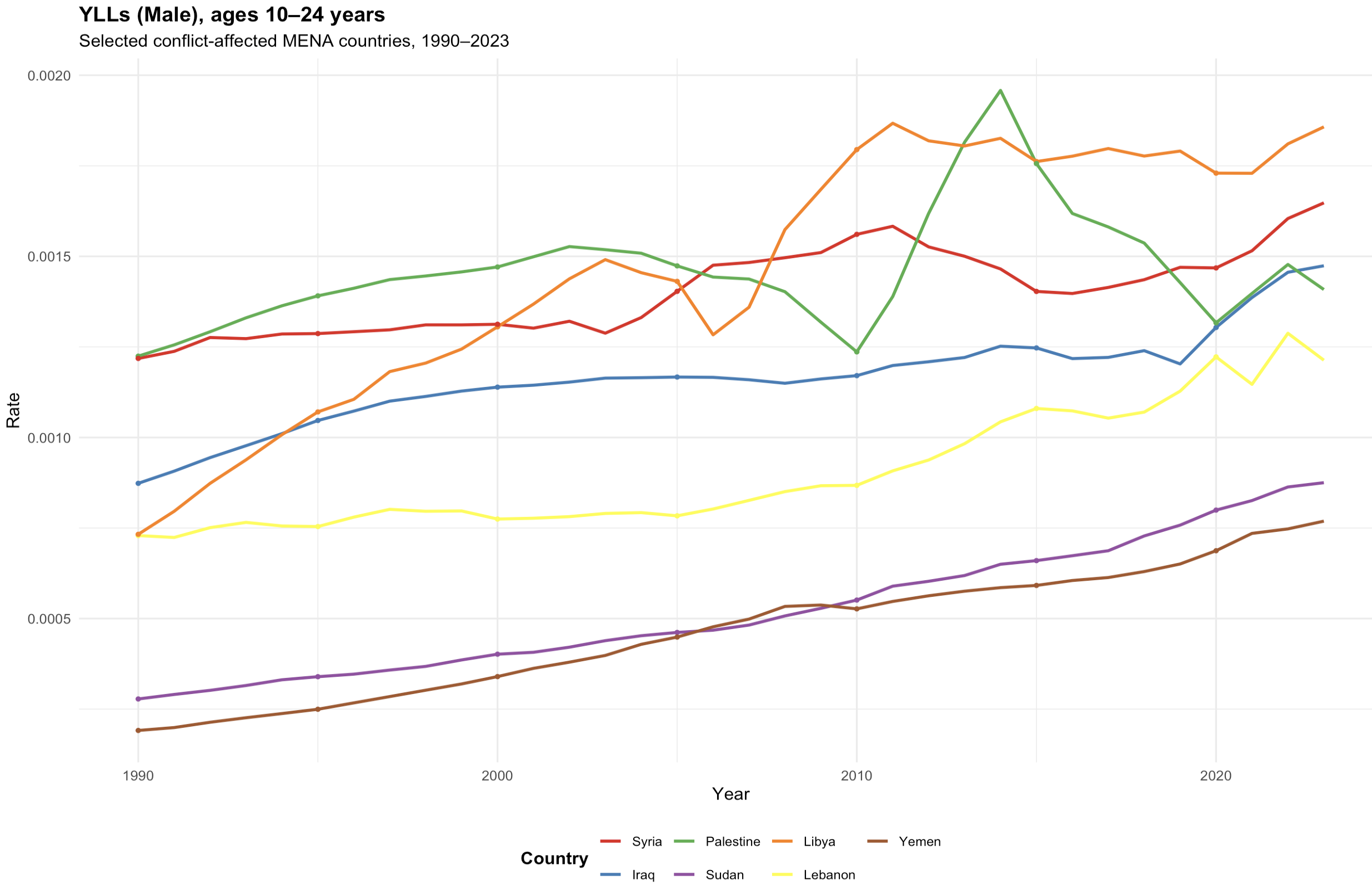
**

**B.**

**
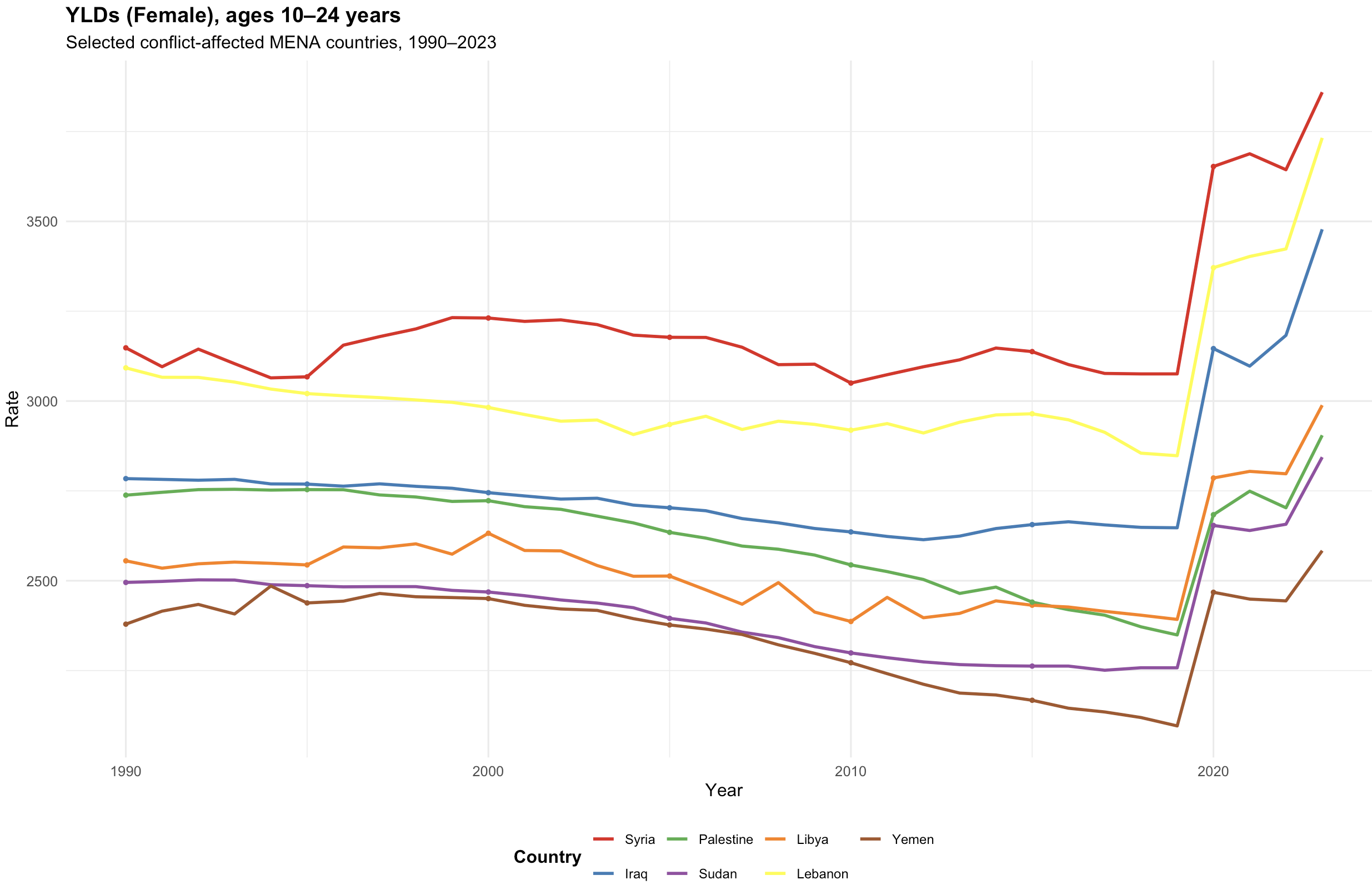
**

**Appendix Figure 21. Relation between DALY rates per 100,000 population of five NCDs and Socio-Demographic Index (SDI), in MENA, year 2023, both sexes, age 10-24**

**
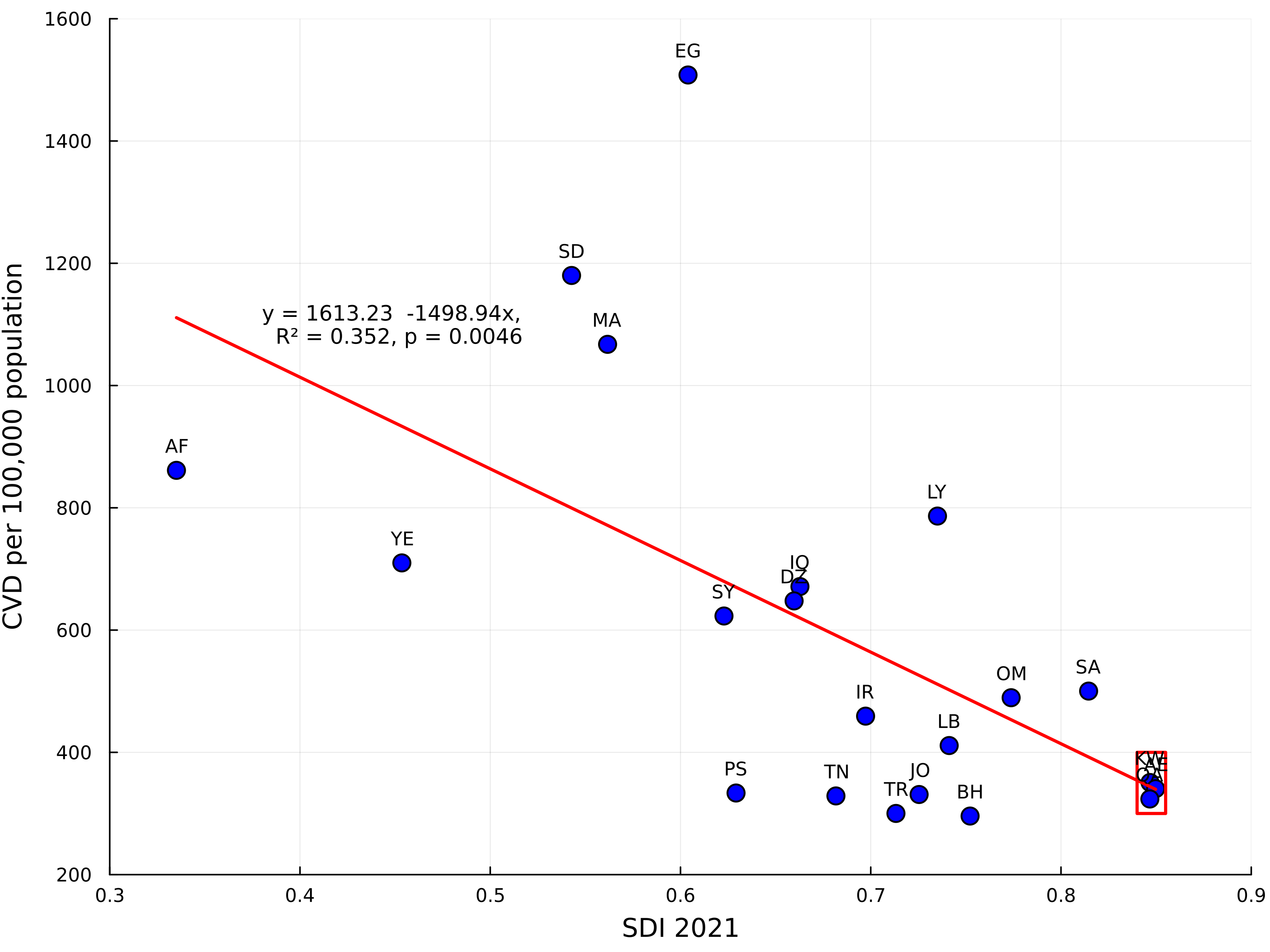

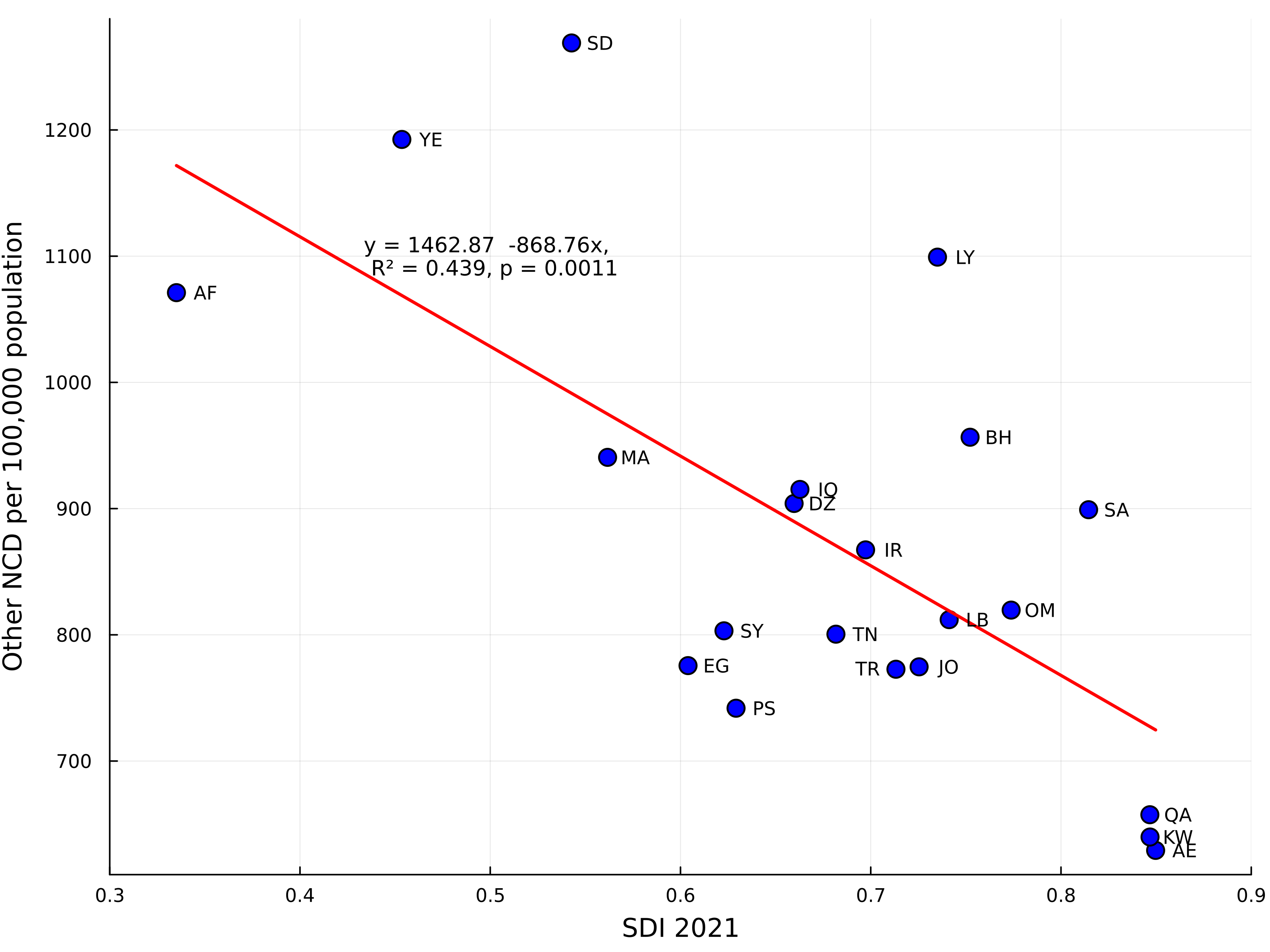
**

**
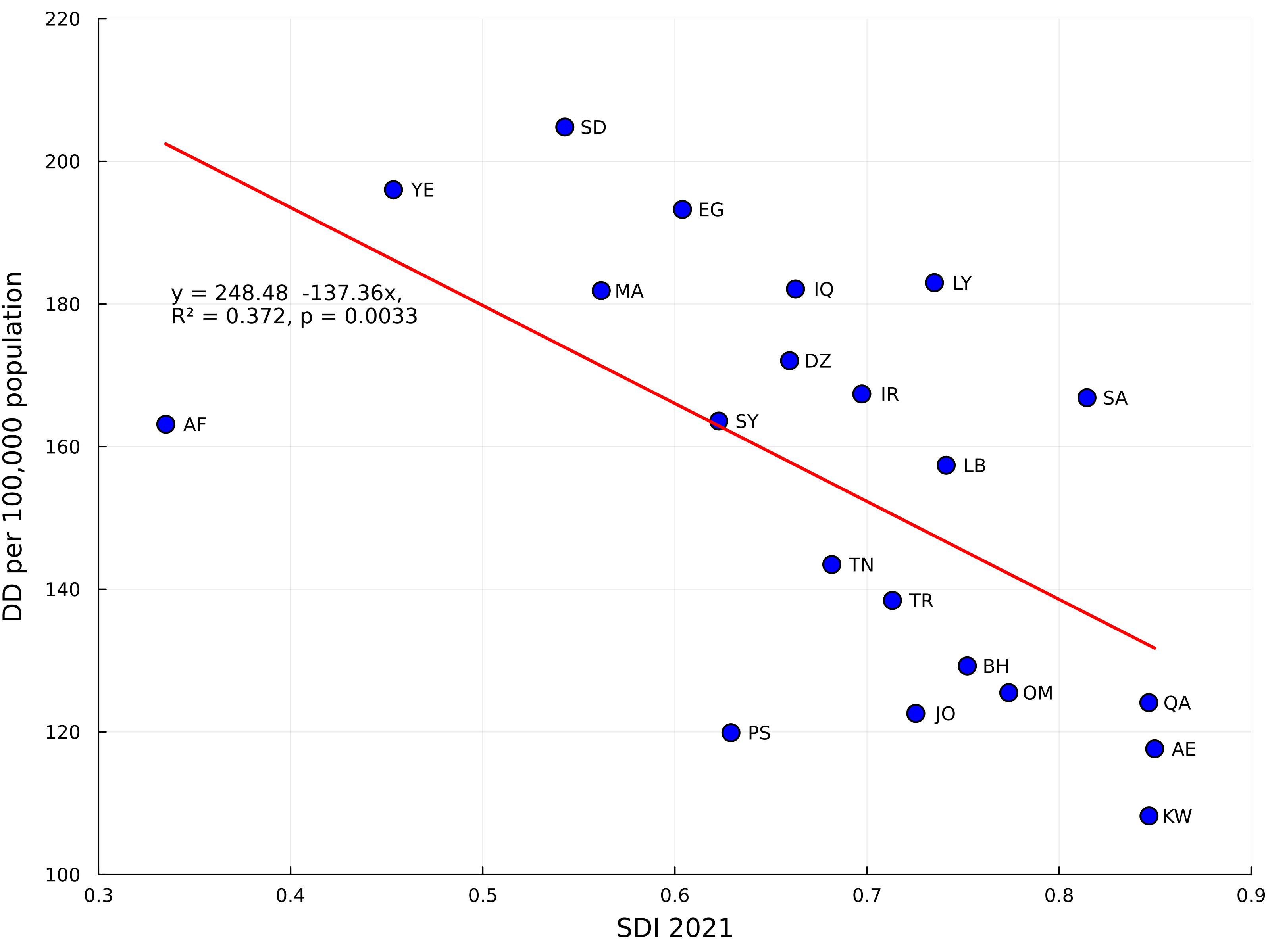

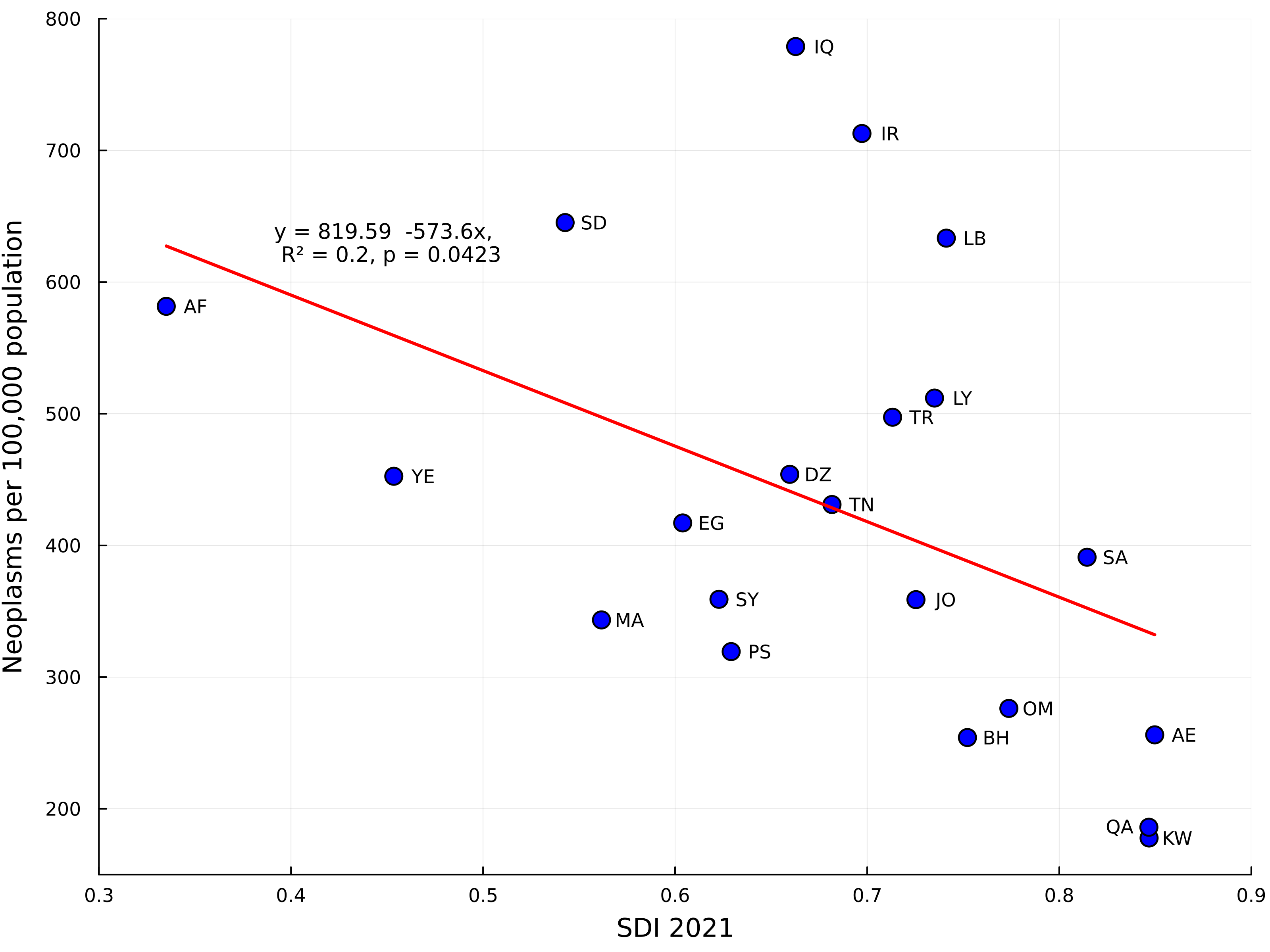
**

Abbreviations: CVD=Cardiovascular disease, NCD=Non-communicable disease, DD=Digestive diseases, SDI=Sociodemographic index, AE = United Arab Emirates; AF = Afghanistan; BH = Bahrain; DZ = Algeria; EG = Egypt; IQ = Iraq; IR = Iran; JO = Jordan; KW = Kuwait; LB = Lebanon; LY = Libya; MA = Morocco; MENA = Middle East and North Africa; OM = Oman; PS = Palestine; QA = Qatar; SA = Saudi Arabia; SD = Sudan; SY = Syrian Arab Republic; TN = Tunisia; TR = Turkiye; YE = Yemen;

**Appendix Table 1: Global Burden of Disease 2021 Cause Hierarchy**

| **Cause ID** | **Cause Name** | **Hierarchy Level** | **Cause Outline** |
| --- | --- | --- | --- |
| 294 | All causes | 0 | Total |
| 295 | Communicable, maternal, neonatal, and nutritional diseases | 1 | A |
| 409 | Non-communicable diseases | 1 | B |
| 410 | Neoplasms | 2 | B.1 |
| 444 | Lip and oral cavity cancer | 3 | B.1.1 |
| 447 | Nasopharynx cancer | 3 | B.1.2 |
| 450 | Other pharynx cancer | 3 | B.1.3 |
| 411 | Esophageal cancer | 3 | B.1.4 |
| 414 | Stomach cancer | 3 | B.1.5 |
| 441 | Colon and rectum cancer | 3 | B.1.6 |
| 417 | Liver cancer | 3 | B.1.7 |
| 453 | Gallbladder and biliary tract cancer | 3 | B.1.8 |
| 456 | Pancreatic cancer | 3 | B.1.9 |
| 423 | Larynx cancer | 3 | B.1.10 |
| 426 | Tracheal, bronchus, and lung cancer | 3 | B.1.11 |
| 459 | Malignant skin melanoma | 3 | B.1.12 |
| 462 | Non-melanoma skin cancer | 3 | B.1.13 |
| 1011 | Soft tissue and other extraosseous sarcomas | 3 | B.1.14 |
| 1012 | Malignant neoplasm of bone and articular cartilage | 3 | B.1.15 |
| 429 | Breast cancer | 3 | B.1.16 |
| 432 | Cervical cancer | 3 | B.1.17 |
| 435 | Uterine cancer | 3 | B.1.18 |
| 465 | Ovarian cancer | 3 | B.1.19 |
| 438 | Prostate cancer | 3 | B.1.20 |
| 468 | Testicular cancer | 3 | B.1.21 |
| 471 | Kidney cancer | 3 | B.1.22 |
| 474 | Bladder cancer | 3 | B.1.23 |
| 477 | Brain and central nervous system cancer | 3 | B.1.24 |
| 1008 | Eye cancer | 3 | B.1.25 |
| 1013 | Neuroblastoma and other peripheral nervous cell tumors | 3 | B.1.26 |
| 480 | Thyroid cancer | 3 | B.1.27 |
| 483 | Mesothelioma | 3 | B.1.28 |
| 484 | Hodgkin lymphoma | 3 | B.1.29 |
| 485 | Non-Hodgkin lymphoma | 3 | B.1.30 |
| 486 | Multiple myeloma | 3 | B.1.31 |
| 487 | Leukemia | 3 | B.1.32 |
| 489 | Other malignant neoplasms | 3 | B.1.33 |
| 490 | Other neoplasms | 3 | B.1.34 |
| 491 | Cardiovascular diseases | 2 | B.2 |
| 492 | Rheumatic heart disease | 3 | B.2.1 |
| 493 | Ischemic heart disease | 3 | B.2.2 |
| 494 | Stroke | 3 | B.2.3 |
| 498 | Hypertensive heart disease | 3 | B.2.4 |
| 504 | Non-rheumatic valvular heart disease | 3 | B.2.5 |
| 499 | Cardiomyopathy and myocarditis | 3 | B.2.6 |
| 1004 | Pulmonary Arterial Hypertension | 3 | B.2.7 |
| 500 | Atrial fibrillation and flutter | 3 | B.2.8 |
| 501 | Aortic aneurysm | 3 | B.2.9 |
| 502 | Lower extremity peripheral arterial disease | 3 | B.2.10 |
| 503 | Endocarditis | 3 | B.2.11 |
| 507 | Other cardiovascular and circulatory diseases | 3 | B.2.12 |
| 508 | Chronic respiratory diseases | 2 | B.3 |
| 509 | Chronic obstructive pulmonary disease | 3 | B.3.1 |
| 510 | Pneumoconiosis | 3 | B.3.2 |
| 515 | Asthma | 3 | B.3.3 |
| 516 | Interstitial lung disease and pulmonary sarcoidosis | 3 | B.3.4 |
| 520 | Other chronic respiratory diseases | 3 | B.3.5 |
| 526 | Digestive diseases | 2 | B.4 |
| 521 | Cirrhosis and other chronic liver diseases | 3 | B.4.1 |
| 992 | Upper digestive system diseases | 3 | B.4.2 |
| 529 | Appendicitis | 3 | B.4.3 |
| 530 | Paralytic ileus and intestinal obstruction | 3 | B.4.4 |
| 531 | Inguinal, femoral, and abdominal hernia | 3 | B.4.5 |
| 532 | Inflammatory bowel disease | 3 | B.4.6 |
| 533 | Vascular intestinal disorders | 3 | B.4.7 |
| 534 | Gallbladder and biliary diseases | 3 | B.4.8 |
| 535 | Pancreatitis | 3 | B.4.9 |
| 541 | Other digestive diseases | 3 | B.4.10 |
| 542 | Neurological disorders | 2 | B.5 |
| 543 | Alzheimer's disease and other dementias | 3 | B.5.1 |
| 544 | Parkinson's disease | 3 | B.5.2 |
| 545 | Idiopathic epilepsy | 3 | B.5.3 |
| 546 | Multiple sclerosis | 3 | B.5.4 |
| 554 | Motor neuron disease | 3 | B.5.5 |
| 972 | Headache disorders | 3 | B.5.6 |
| 557 | Other neurological disorders | 3 | B.5.7 |
| 558 | Mental disorders | 2 | B.6 |
| 559 | Schizophrenia | 3 | B.6.1 |
| 567 | Depressive disorders | 3 | B.6.2 |
| 570 | Bipolar disorder | 3 | B.6.3 |
| 571 | Anxiety disorders | 3 | B.6.4 |
| 572 | Eating disorders | 3 | B.6.5 |
| 575 | Autism spectrum disorders | 3 | B.6.6 |
| 578 | Attention-deficit/hyperactivity disorder | 3 | B.6.7 |
| 579 | Conduct disorder | 3 | B.6.8 |
| 582 | Idiopathic developmental intellectual disability | 3 | B.6.9 |
| 585 | Other mental disorders | 3 | B.6.10 |
| 973 | Substance use disorders | 2 | B.7 |
| 560 | Alcohol use disorders | 3 | B.7.1 |
| 561 | Drug use disorders | 3 | B.7.2 |
| 974 | Diabetes and kidney diseases | 2 | B.8 |
| 587 | Diabetes mellitus | 3 | B.8.1 |
| 589 | Chronic kidney disease | 3 | B.8.2 |
| 588 | Acute glomerulonephritis | 3 | B.8.3 |
| 653 | Skin and subcutaneous diseases | 2 | B.9 |
| 654 | Dermatitis | 3 | B.9.1 |
| 655 | Psoriasis | 3 | B.9.2 |
| 980 | Bacterial skin diseases | 3 | B.9.3 |
| 658 | Scabies | 3 | B.9.4 |
| 659 | Fungal skin diseases | 3 | B.9.5 |
| 660 | Viral skin diseases | 3 | B.9.6 |
| 661 | Acne vulgaris | 3 | B.9.7 |
| 662 | Alopecia areata | 3 | B.9.8 |
| 663 | Pruritus | 3 | B.9.9 |
| 664 | Urticaria | 3 | B.9.10 |
| 665 | Decubitus ulcer | 3 | B.9.11 |
| 668 | Other skin and subcutaneous diseases | 3 | B.9.12 |
| 669 | Sense organ diseases | 2 | B.10 |
| 981 | Blindness and vision loss | 3 | B.10.1 |
| 674 | Age-related and other hearing loss | 3 | B.10.2 |
| 679 | Other sense organ diseases | 3 | B.10.3 |
| 626 | Musculoskeletal disorders | 2 | B.11 |
| 627 | Rheumatoid arthritis | 3 | B.11.1 |
| 628 | Osteoarthritis | 3 | B.11.2 |
| 630 | Low back pain | 3 | B.11.3 |
| 631 | Neck pain | 3 | B.11.4 |
| 632 | Gout | 3 | B.11.5 |
| 639 | Other musculoskeletal disorders | 3 | B.11.6 |
| 640 | Other non-communicable diseases | 2 | B.12 |
| 641 | Congenital birth defects | 3 | B.12.1 |
| 594 | Urinary diseases and male infertility | 3 | B.12.2 |
| 603 | Gynecological diseases | 3 | B.12.3 |
| 613 | Hemoglobinopathies and hemolytic anemias | 3 | B.12.4 |
| 619 | Endocrine, metabolic, blood, and immune disorders | 3 | B.12.5 |
| 680 | Oral disorders | 3 | B.12.6 |
| 686 | Sudden infant death syndrome | 3 | B.12.7 |
| 687 | Injuries | 1 | C |

**Appendix Table 2: Level 1 mortality, YLL, YLD and DALY rates per 100,000 population with 95% UI and respective percentage among age groups, both sexes, in MENA, year 2023**

**(A) Mortality**


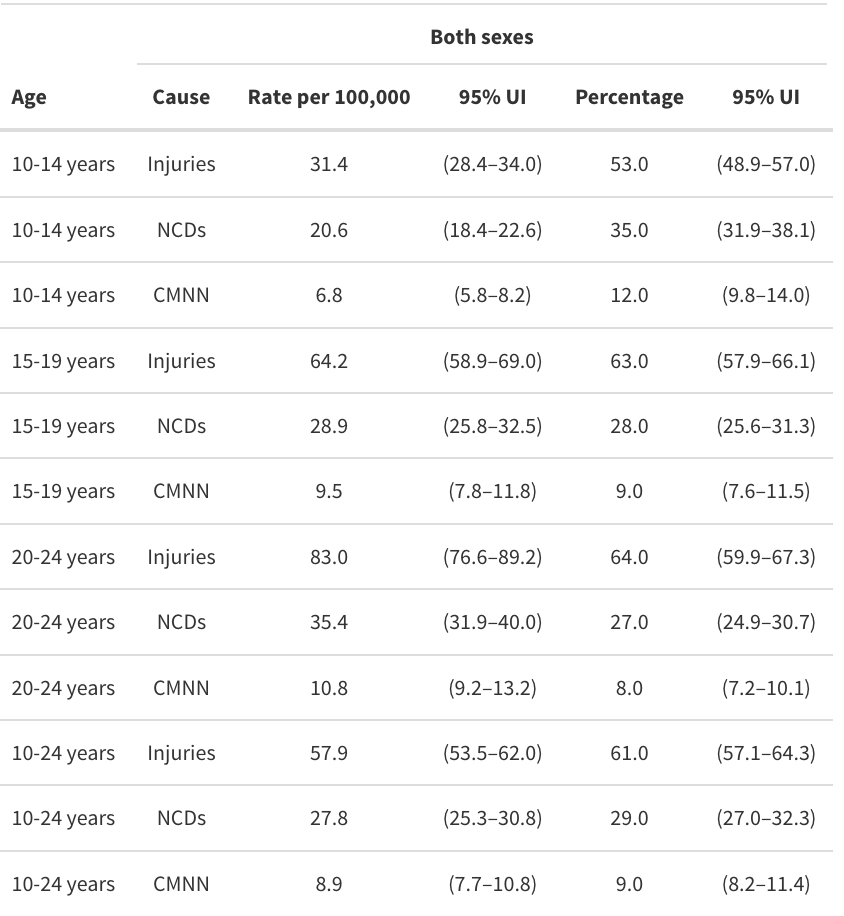


**(B) YLLs**

**
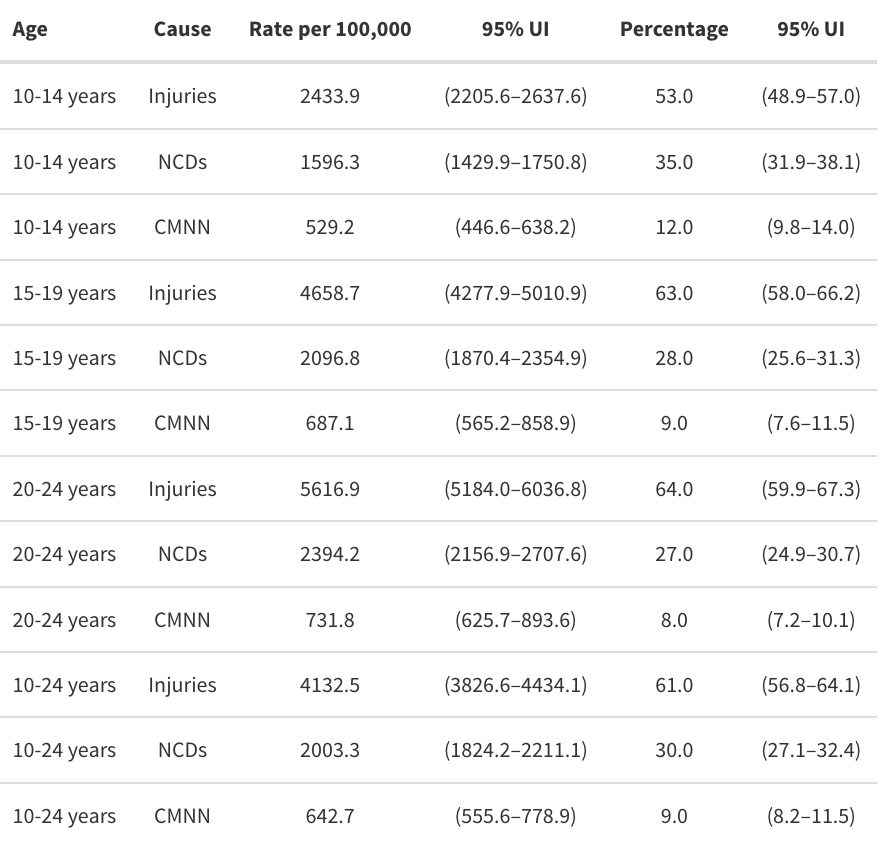
**

**(C) YLDs**

**
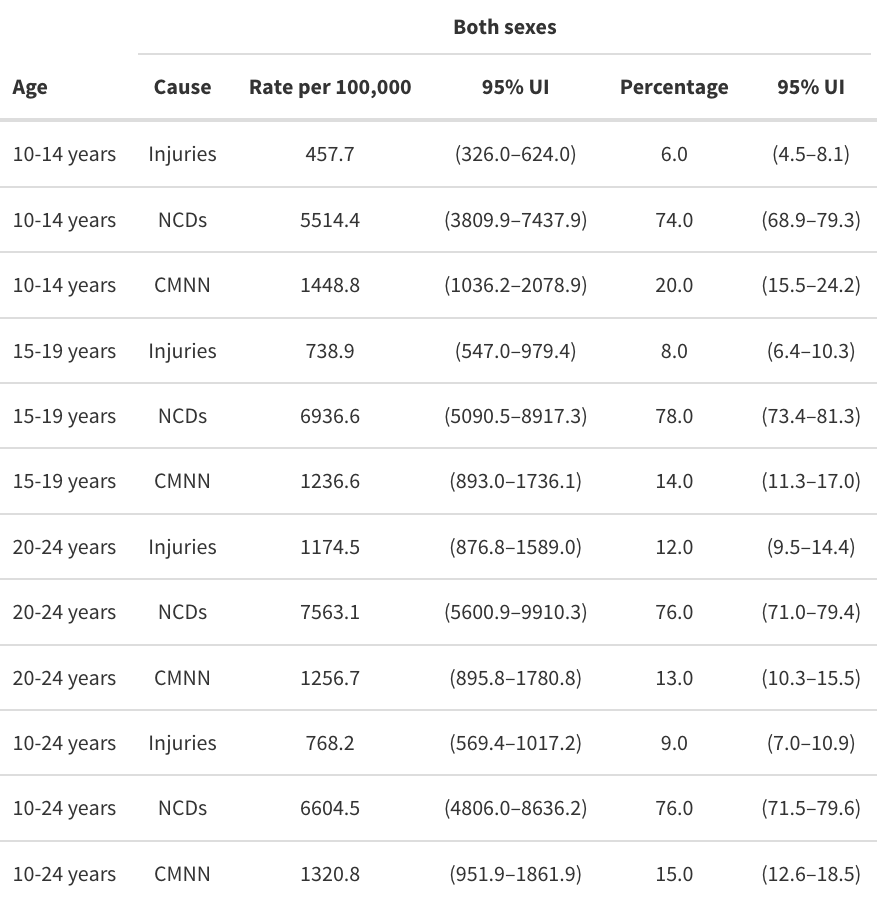
**

**(D) DALYs**


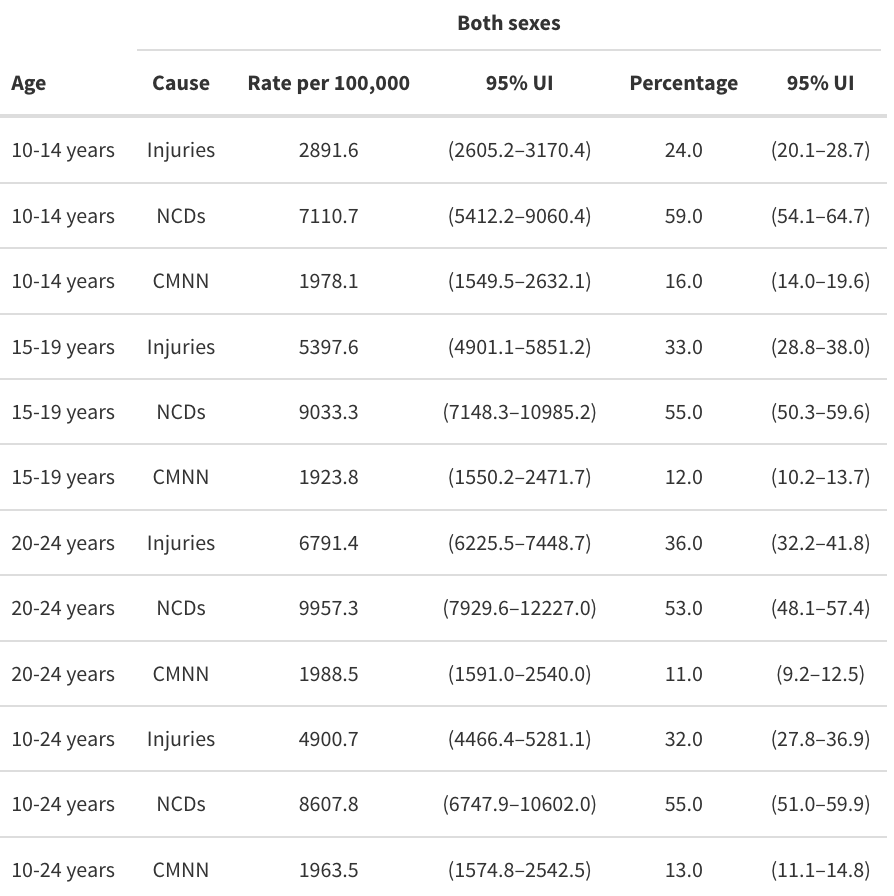


**Appendix Table 3: Level 1 mortality, YLL, YLD and DALY rates per 100,000 population with 95% UI and respective percentage among age groups, by sexes, in MENA, year 2023**

**(A) Mortality**

**
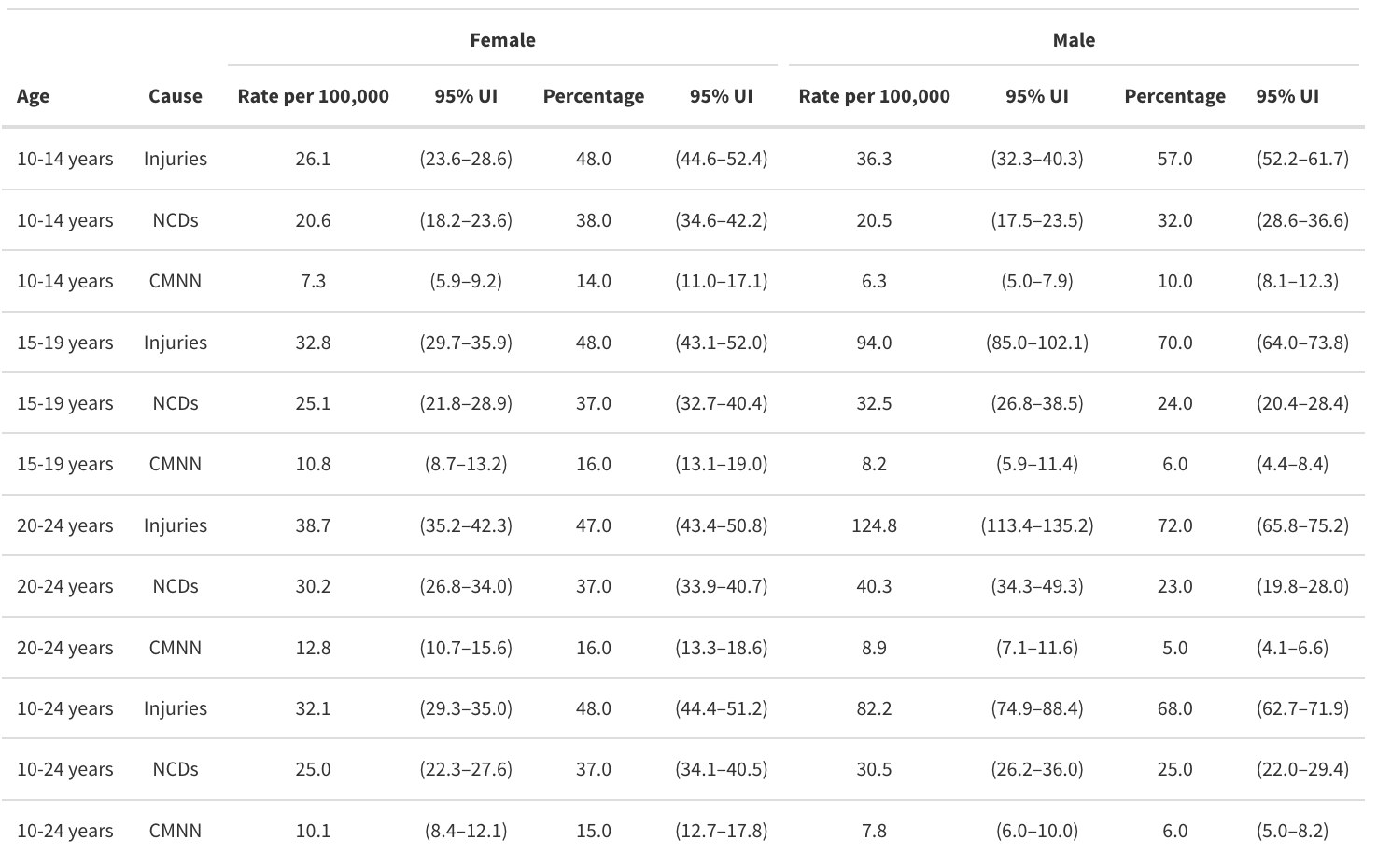
**

**(B) YLLs**

**
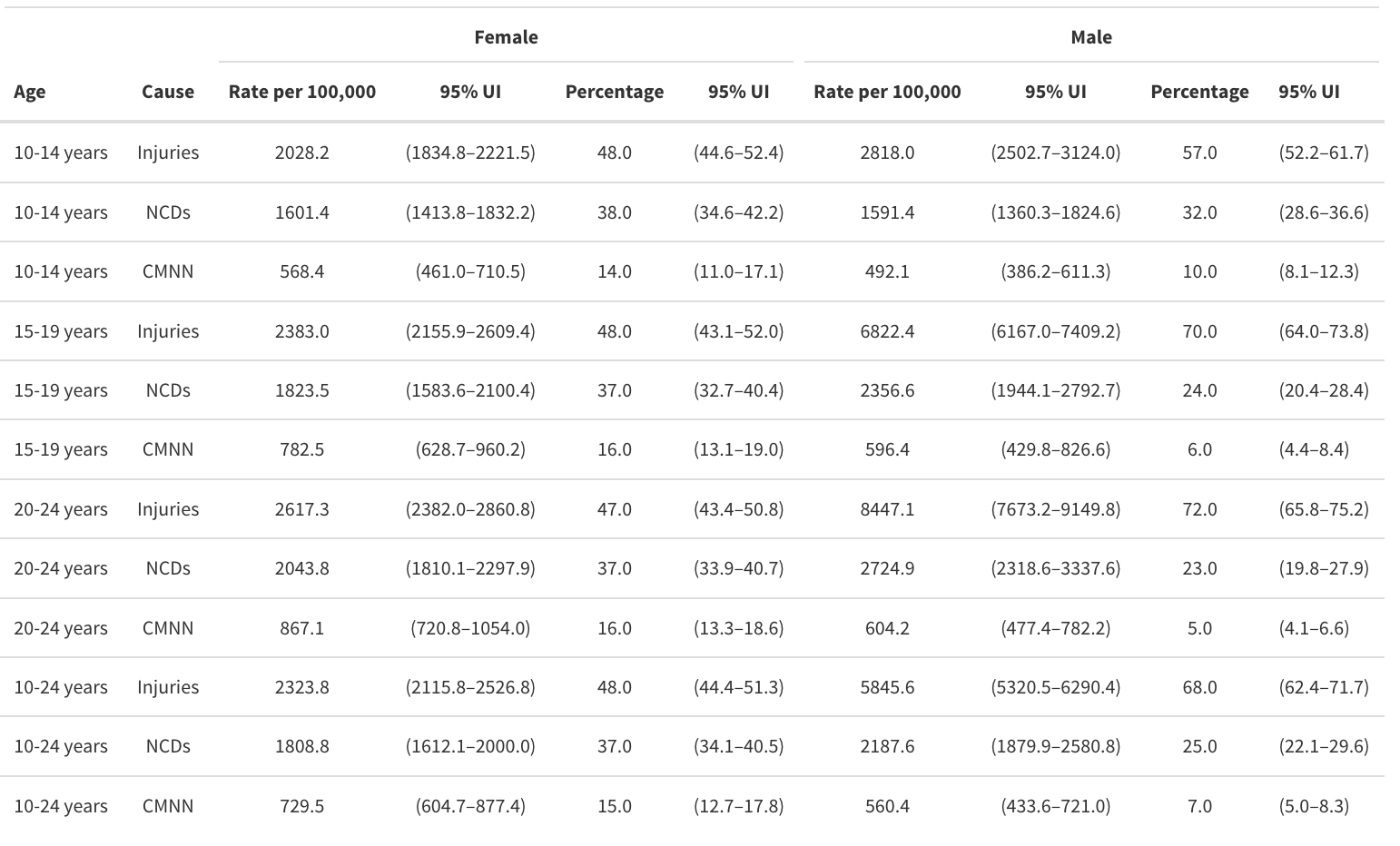
**

**(C) YLDs**

**
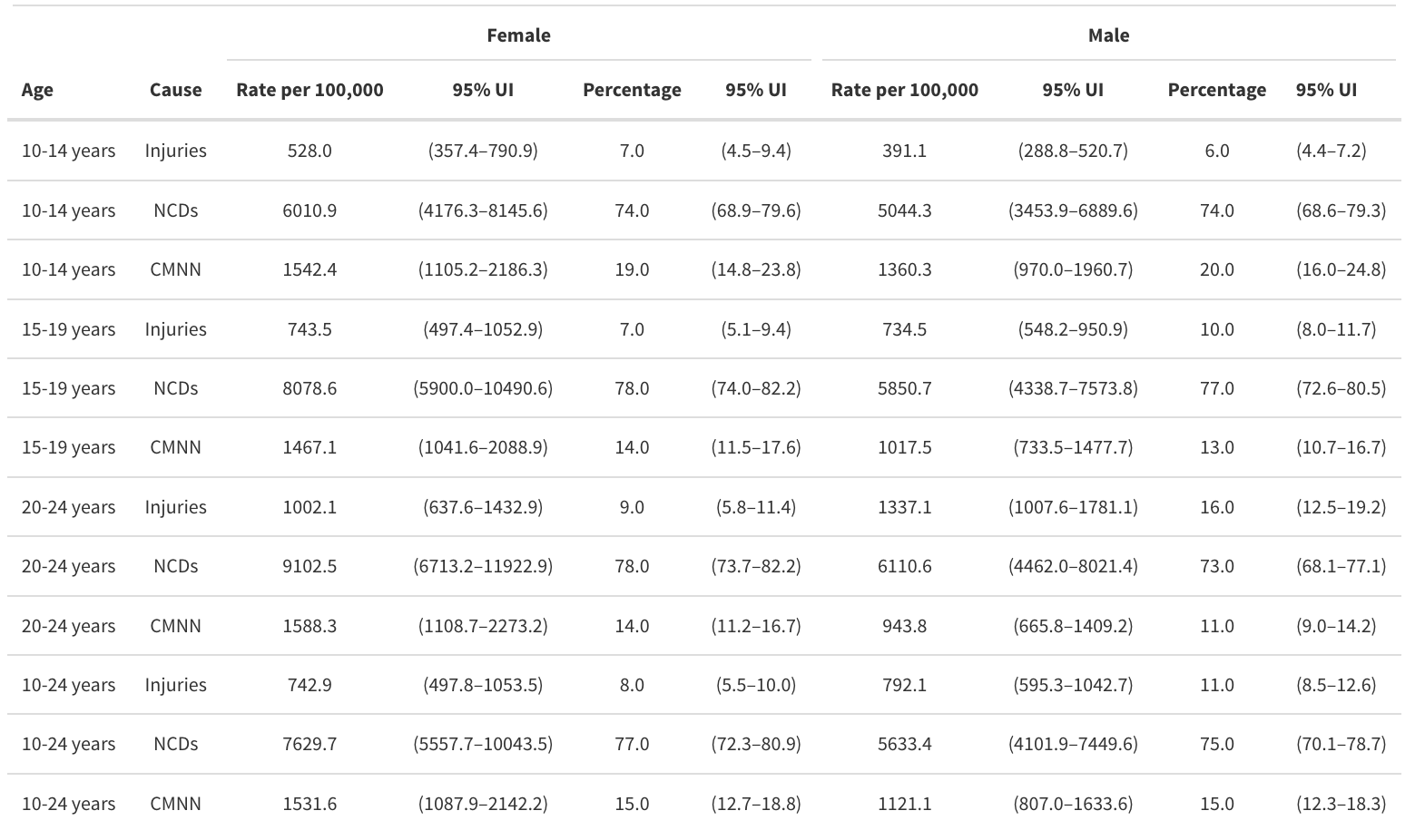
**

**(D) DALYs**


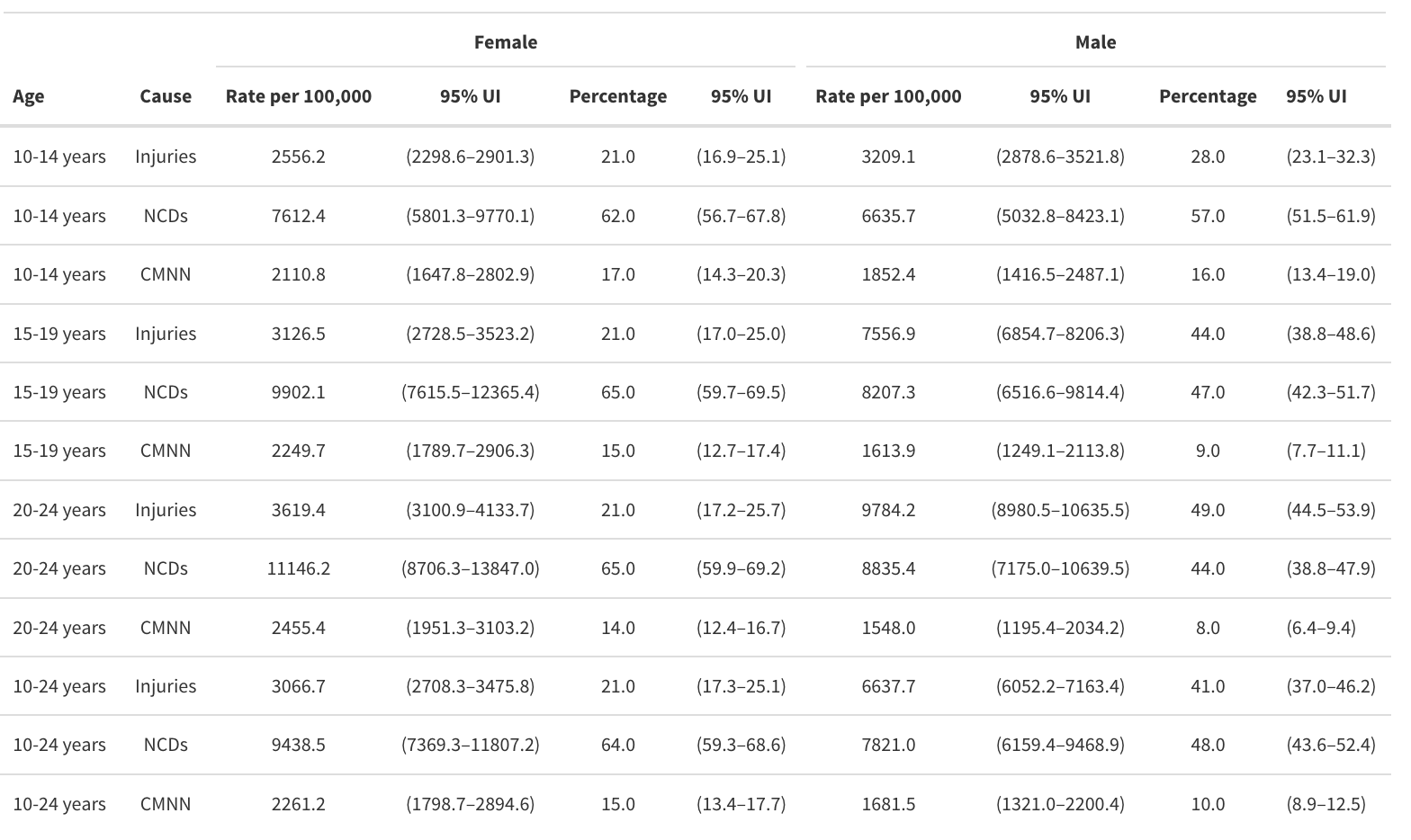


**Appendix Table 4. Adolescents (10-24 years old) YLL rates in MENA 1990 and 2023 and percentage change from 1990 to 2023**

| **10-24 years old YLL rates per 100,000 population** | | | | | | | | | |  |
| --- | --- | --- | --- | --- | --- | --- | --- | --- | --- | --- |
| **Cause** | **1990** | **Upper** | **Lower** | **2023** | **Upper** | **Lower** | **Percentage change** | **%Upper** | **%Lower** | |
| **All NCDs** | **3049.6** | **3445.8** | **2655.2** | **2003.3** | **2211.1** | **1824.2** | **-34.3%** | **-23.2%** | **-44.3%** | |
| **Neoplasms** | **638.3** | **724.7** | **551.6** | **500.9** | **562.2** | **436.5** | **-21.5%** | **-5.7%** | **-33.5%** | |
| Esophageal cancer | 0.9 | 1.2 | 0.6 | 0.8 | 1.0 | 0.6 | -12.4% | 30.3% | -43.5% | |
| Stomach cancer | 15.6 | 21.7 | 10.3 | 9.1 | 11.5 | 6.1 | -42.0% | -10.5% | -61.6% | |
| Liver cancer | 16.2 | 22.1 | 10.3 | 14.9 | 20.9 | 10.0 | -8.0% | 54.4% | -41.4% | |
| Larynx cancer | 1.2 | 1.7 | 0.8 | 1.2 | 1.5 | 0.9 | 0.2% | 41.4% | -32.0% | |
| Tracheal, bronchus, and lung cancer | 13.9 | 18.8 | 10.1 | 18.1 | 23.8 | 13.4 | 29.4% | 99.6% | -13.1% | |
| Breast cancer | 4.3 | 5.8 | 3.1 | 7.7 | 10.6 | 5.3 | 79.0% | 196.1% | 10.7% | |
| Cervical cancer | 4.7 | 7.9 | 2.4 | 3.2 | 5.2 | 1.9 | -31.5% | 44.1% | -64.4% | |
| Uterine cancer | 0.4 | 0.6 | 0.2 | 0.5 | 0.8 | 0.3 | 36.8% | 139.9% | -23.2% | |
| Prostate cancer | 0.3 | 0.4 | 0.2 | 0.4 | 0.6 | 0.3 | 32.1% | 115.7% | -25.1% | |
| Colon and rectum cancer | 10.6 | 14.7 | 7.8 | 13.4 | 17.5 | 9.7 | 27.0% | 89.3% | -13.1% | |
| Lip and oral cavity cancer | 1.7 | 2.3 | 1.2 | 1.8 | 2.4 | 1.4 | 11.0% | 58.7% | -27.0% | |
| Nasopharynx cancer | 6.3 | 8.9 | 4.2 | 3.4 | 4.6 | 2.2 | -46.7% | -16.3% | -70.8% | |
| Other pharynx cancer | 0.3 | 0.4 | 0.2 | 0.4 | 0.5 | 0.3 | 34.9% | 105.7% | -9.5% | |
| Gallbladder and biliary tract cancer | 0.6 | 0.7 | 0.4 | 0.6 | 0.8 | 0.4 | 3.2% | 36.8% | -30.0% | |
| Pancreatic cancer | 1.3 | 1.7 | 0.9 | 2.3 | 3.0 | 1.7 | 73.6% | 162.8% | 20.6% | |
| Malignant skin melanoma | 1.0 | 1.4 | 0.5 | 0.9 | 1.3 | 0.6 | -0.9% | 38.5% | -37.8% | |
| Non-melanoma skin cancer | 0.4 | 0.6 | 0.3 | 0.7 | 1.1 | 0.5 | 72.5% | 187.6% | 9.8% | |
| Ovarian cancer | 3.1 | 4.7 | 2.1 | 5.0 | 7.1 | 3.4 | 62.2% | 179.3% | -11.3% | |
| Testicular cancer | 3.6 | 5.6 | 2.2 | 3.7 | 5.4 | 2.4 | 2.6% | 104.3% | -46.0% | |
| Kidney cancer | 4.5 | 6.0 | 3.0 | 4.3 | 5.7 | 3.0 | -3.1% | 37.7% | -36.8% | |
| Bladder cancer | 2.0 | 2.9 | 1.3 | 1.5 | 2.0 | 1.0 | -25.8% | 35.6% | -52.0% | |
| Brain and central nervous system cancer | 94.0 | 125.0 | 65.4 | 88.4 | 111.9 | 66.2 | -6.0% | 24.0% | -26.5% | |
| Thyroid cancer | 2.3 | 3.2 | 1.7 | 2.1 | 2.8 | 1.6 | -8.4% | 37.9% | -38.6% | |
| Mesothelioma | 0.4 | 0.6 | 0.3 | 0.5 | 0.7 | 0.3 | 20.2% | 98.2% | -24.6% | |
| Hodgkin lymphoma | 21.1 | 31.3 | 11.8 | 10.6 | 15.8 | 5.5 | -49.8% | -18.2% | -68.1% | |
| Non-Hodgkin lymphoma | 42.1 | 54.9 | 31.0 | 36.2 | 47.5 | 27.2 | -14.0% | 29.7% | -43.4% | |
| Multiple myeloma | 0.6 | 0.9 | 0.4 | 1.1 | 1.5 | 0.8 | 69.3% | 184.3% | 2.0% | |
| Leukemia | 239.5 | 308.5 | 184.6 | 163.5 | 199.7 | 131.5 | -31.7% | -10.3% | -50.4% | |
| Other malignant neoplasms | 64.0 | 84.5 | 48.5 | 39.8 | 50.8 | 30.0 | -37.8% | -7.1% | -58.9% | |
| Eye cancer | 0.2 | 0.3 | 0.1 | 0.2 | 0.3 | 0.1 | -8.9% | 32.8% | -46.7% | |
| Soft tissue and other extraosseous sarcomas | 11.7 | 19.4 | 7.3 | 8.7 | 13.2 | 5.6 | -25.6% | 17.0% | -58.9% | |
| Malignant neoplasm of bone and articular cartilage | 59.4 | 97.1 | 38.6 | 47.6 | 68.0 | 35.1 | -19.9% | 27.5% | -48.9% | |
| Neuroblastoma and other peripheral nervous cell tumors | 1.5 | 2.2 | 1.0 | 1.8 | 2.6 | 1.2 | 20.7% | 97.3% | -21.3% | |
| Other neoplasms | 8.8 | 13.8 | 5.0 | 6.7 | 10.2 | 4.2 | -23.5% | 16.0% | -53.6% | |
| **CVDs** | **1236.5** | **1553.5** | **932.5** | **687.2** | **864.5** | **544.4** | **-44.4%** | **-21.7%** | **-60.7%** | |
| Rheumatic heart disease | 183.2 | 257.8 | 103.4 | 53.6 | 87.2 | 31.4 | -70.7% | -46.0% | -85.0% | |
| Ischemic heart disease | 338.9 | 471.7 | 237.3 | 245.8 | 326.5 | 183.4 | -27.5% | 10.7% | -54.5% | |
| Stroke | 476.7 | 650.5 | 329.2 | 218.9 | 303.8 | 152.0 | -54.1% | -22.3% | -70.8% | |
| Hypertensive heart disease | 29.2 | 46.5 | 16.2 | 23.4 | 34.6 | 15.6 | -19.8% | 43.1% | -55.3% | |
| Cardiomyopathy and myocarditis | 51.4 | 85.8 | 27.9 | 33.5 | 56.8 | 20.3 | -34.8% | 25.8% | -67.4% | |
| Aortic aneurysm | 1.4 | 2.2 | 0.8 | 2.4 | 3.5 | 1.6 | 74.4% | 265.3% | -12.6% | |
| Endocarditis | 11.2 | 18.0 | 7.1 | 7.0 | 10.8 | 4.8 | -37.4% | 12.1% | -64.3% | |
| Non-rheumatic valvular heart disease | 15.0 | 23.5 | 8.1 | 14.5 | 22.1 | 8.8 | -2.9% | 70.3% | -43.0% | |
| Pulmonary Arterial Hypertension | 13.8 | 24.4 | 6.8 | 5.9 | 10.2 | 3.3 | -57.0% | -14.4% | -80.6% | |
| Other cardiovascular and circulatory diseases | 115.8 | 178.4 | 67.9 | 82.1 | 111.9 | 56.8 | -29.1% | 22.0% | -58.8% | |
| **Chronic respiratory diseases** | **94.9** | **138.3** | **56.0** | **59.2** | **79.0** | **44.2** | **-37.7%** | **13.4%** | **-63.5%** | |
| Chronic obstructive pulmonary disease | 28.6 | 45.7 | 14.0 | 20.9 | 28.8 | 14.3 | -26.9% | 44.7% | -60.6% | |
| Pneumoconiosis | 0.6 | 1.3 | 0.3 | 0.6 | 1.2 | 0.3 | 2.0% | 188.5% | -67.8% | |
| Asthma | 47.6 | 74.8 | 26.4 | 20.8 | 31.1 | 14.0 | -56.3% | -16.5% | -75.4% | |
| Interstitial lung disease and pulmonary sarcoidosis | 3.0 | 6.0 | 1.3 | 3.6 | 5.9 | 2.1 | 22.3% | 158.0% | -57.9% | |
| Other chronic respiratory diseases | 15.2 | 27.3 | 7.9 | 13.2 | 19.6 | 8.1 | -13.2% | 89.0% | -57.1% | |
| **Digestive diseases** | **208.6** | **262.8** | **158.2** | **81.1** | **103.0** | **62.1** | **-61.1%** | **-39.9%** | **-72.9%** | |
| Cirrhosis and other chronic liver diseases | 144.3 | 185.5 | 106.3 | 53.8 | 69.0 | 40.6 | -62.7% | -44.1% | -75.8% | |
| Upper digestive system diseases | 27.7 | 43.4 | 17.8 | 6.6 | 11.5 | 4.1 | -76.1% | -57.6% | -87.4% | |
| Appendicitis | 9.3 | 15.3 | 4.0 | 2.9 | 4.6 | 1.7 | -68.4% | -25.6% | -84.7% | |
| Paralytic ileus and intestinal obstruction | 11.3 | 16.8 | 7.0 | 6.0 | 9.5 | 4.0 | -46.8% | 5.6% | -72.5% | |
| Inguinal, femoral, and abdominal hernia | 0.7 | 1.4 | 0.3 | 0.3 | 0.5 | 0.1 | -62.3% | 16.8% | -84.1% | |
| Inflammatory bowel disease | 3.9 | 6.5 | 2.2 | 2.8 | 4.4 | 1.7 | -27.3% | 59.6% | -64.1% | |
| Vascular intestinal disorders | 1.1 | 1.7 | 0.6 | 0.8 | 1.3 | 0.5 | -27.9% | 48.2% | -66.5% | |
| Gallbladder and biliary diseases | 2.0 | 3.3 | 1.1 | 1.7 | 2.7 | 1.0 | -14.9% | 58.7% | -60.8% | |
| Pancreatitis | 3.2 | 5.5 | 1.6 | 2.0 | 3.1 | 1.2 | -38.2% | 22.4% | -67.6% | |
| Other digestive diseases | 5.2 | 8.1 | 2.9 | 4.2 | 6.6 | 2.5 | -19.4% | 51.3% | -58.3% | |
| **Neurological disorders** | **112.8** | **152.3** | **71.7** | **103.6** | **127.6** | **84.2** | **-8.2%** | **44.7%** | **-34.9%** | |
| Parkinson's disease | 0.0 | 0.1 | 0.0 | 0.1 | 0.1 | 0.0 | 25.2% | 64.0% | -11.0% | |
| Idiopathic epilepsy | 104.3 | 143.4 | 63.2 | 70.1 | 91.7 | 52.8 | -32.8% | 10.7% | -53.3% | |
| Multiple sclerosis | 1.3 | 2.8 | 0.6 | 1.7 | 3.0 | 0.8 | 31.7% | 281.8% | -48.6% | |
| Motor neuron disease | 0.8 | 1.7 | 0.4 | 3.0 | 5.9 | 1.7 | 285.6% | 785.6% | 56.0% | |
| Other neurological disorders | 6.4 | 10.6 | 3.7 | 28.7 | 37.9 | 21.7 | 348.8% | 781.6% | 133.5% | |
| **Mental disorders** | **0.0** | **0.1** | **0.0** | **0.1** | **0.1** | **0.0** | **128.0%** | **1354.2%** | **-46.5%** | |
| Eating disorders | 0.0 | 0.1 | 0.0 | 0.1 | 0.1 | 0.0 | 128.0% | 1354.2% | -46.5% | |
| **Substance use disorders** | **44.6** | **62.3** | **31.2** | **50.0** | **72.0** | **35.7** | **12.0%** | **84.9%** | **-34.5%** | |
| Alcohol use disorders | 5.9 | 8.6 | 3.8 | 3.2 | 4.9 | 2.1 | -45.0% | -1.1% | -69.3% | |
| Drug use disorders | 38.7 | 56.5 | 25.6 | 46.7 | 68.8 | 32.3 | 20.6% | 108.7% | -31.3% | |
| **Diabetes and kidney diseases** | **181.7** | **235.6** | **138.7** | **156.4** | **191.6** | **121.4** | **-13.9%** | **15.9%** | **-41.7%** | |
| Diabetes mellitus | 45.2 | 65.7 | 31.9 | 36.0 | 46.1 | 27.6 | -20.3% | 21.4% | -43.2% | |
| Chronic kidney disease | 135.3 | 188.1 | 95.5 | 119.7 | 151.7 | 88.8 | -11.6% | 28.0% | -45.2% | |
| Acute glomerulonephritis | 1.1 | 2.2 | 0.4 | 0.7 | 1.1 | 0.4 | -38.0% | 73.3% | -75.4% | |
| **Skin and subcutaneous diseases** | **3.2** | **5.0** | **1.6** | **2.9** | **4.3** | **1.7** | **-10.6%** | **75.9%** | **-56.3%** | |
| Bacterial skin diseases | 0.4 | 1.1 | 0.0 | 0.7 | 1.2 | 0.3 | 59.7% | 2114.1% | -54.5% | |
| Decubitus ulcer | 2.0 | 3.3 | 1.0 | 1.5 | 2.6 | 0.8 | -23.0% | 58.0% | -63.2% | |
| Other skin and subcutaneous diseases | 0.8 | 1.4 | 0.3 | 0.7 | 1.2 | 0.3 | -15.9% | 79.3% | -64.3% | |
| **Musculoskeletal disorders** | **17.6** | **26.0** | **10.9** | **14.7** | **20.0** | **9.8** | **-16.5%** | **37.5%** | **-46.4%** | |
| Rheumatoid arthritis | 0.8 | 1.6 | 0.3 | 0.4 | 0.8 | 0.2 | -43.0% | 33.1% | -72.9% | |
| Other musculoskeletal disorders | 16.8 | 25.2 | 10.3 | 14.2 | 19.4 | 9.6 | -15.2% | 40.8% | -45.8% | |
| **Other non-communicable diseases** | **511.3** | **630.7** | **415.7** | **347.4** | **420.6** | **285.5** | **-32.0%** | **-10.4%** | **-48.3%** | |
| Congenital birth defects | 356.6 | 470.6 | 262.4 | 241.2 | 310.2 | 182.0 | -32.4% | -1.9% | -53.9% | |
| Urinary diseases and male infertility | 10.8 | 13.9 | 8.7 | 7.2 | 8.7 | 6.0 | -33.2% | -9.7% | -50.8% | |
| Gynecological diseases | 1.1 | 2.0 | 0.5 | 0.7 | 1.4 | 0.4 | -31.3% | 76.4% | -75.6% | |
| Hemoglobinopathies and hemolytic anemias | 106.9 | 138.4 | 83.7 | 52.6 | 68.3 | 38.6 | -50.8% | -21.3% | -66.3% | |
| Endocrine, metabolic, blood, and immune disorders | 35.9 | 51.2 | 23.7 | 45.7 | 62.5 | 31.8 | 27.2% | 105.6% | -19.8% | |

**Appendix Table 5. Adolescents (10-24 years old) YLD rates in MENA 1990 and 2023 and percentage change from 1990 to 2023**

| **10-24 years old YLL rates per 100,000 population** | | | | | | | | | | |
| --- | --- | --- | --- | --- | --- | --- | --- | --- | --- | --- |
| **Cause** | **1990** | **upper** | **lower** | **2023** | **upper** | **lower** | **percentage change** | **upper** | **lower** |  |
| **Non-communicable diseases** | **6079.09** | **7917.90** | **4450.17** | **6604.46** | **8636.22** | **4805.99** | **8.6%** | **21.9%** | **-3.4%** |  |
| **Neoplasms** | **6.17** | **9.24** | **4.21** | **9.82** | **15.31** | **6.05** | **59.1%** | **163.8%** | **5.7%** |  |
| Esophageal cancer | 0.01 | 0.01 | 0.00 | 0.01 | 0.01 | 0.01 | 48.9% | 134.3% | -10.1% |  |
| Stomach cancer | 0.12 | 0.18 | 0.07 | 0.13 | 0.21 | 0.07 | 13.1% | 99.2% | -31.5% |  |
| Liver cancer | 0.08 | 0.14 | 0.04 | 0.12 | 0.18 | 0.07 | 42.6% | 170.2% | -23.1% |  |
| Larynx cancer | 0.01 | 0.02 | 0.01 | 0.02 | 0.02 | 0.01 | 40.3% | 101.3% | -5.1% |  |
| Tracheal, bronchus, and lung cancer | 0.05 | 0.08 | 0.03 | 0.07 | 0.11 | 0.05 | 39.0% | 125.2% | -7.3% |  |
| Breast cancer | 0.14 | 0.23 | 0.08 | 0.49 | 0.81 | 0.27 | 241.7% | 510.8% | 91.1% |  |
| Cervical cancer | 0.11 | 0.22 | 0.05 | 0.12 | 0.23 | 0.06 | 14.6% | 185.5% | -45.4% |  |
| Uterine cancer | 0.01 | 0.02 | 0.01 | 0.02 | 0.04 | 0.01 | 158.6% | 386.8% | 43.3% |  |
| Prostate cancer | 0.01 | 0.02 | 0.01 | 0.04 | 0.06 | 0.02 | 244.0% | 539.8% | 81.2% |  |
| Colon and rectum cancer | 0.16 | 0.25 | 0.10 | 0.43 | 0.69 | 0.26 | 177.3% | 359.9% | 75.3% |  |
| Lip and oral cavity cancer | 0.03 | 0.05 | 0.02 | 0.06 | 0.10 | 0.04 | 86.3% | 193.5% | 18.4% |  |
| Nasopharynx cancer | 0.11 | 0.22 | 0.06 | 0.18 | 0.33 | 0.08 | 53.8% | 235.2% | -31.6% |  |
| Other pharynx cancer | 0.00 | 0.00 | 0.00 | 0.00 | 0.00 | 0.00 | 94.5% | 197.1% | 30.5% |  |
| Gallbladder and biliary tract cancer | 0.00 | 0.00 | 0.00 | 0.01 | 0.01 | 0.00 | 117.9% | 217.4% | 42.2% |  |
| Pancreatic cancer | 0.01 | 0.01 | 0.00 | 0.01 | 0.02 | 0.01 | 153.9% | 312.6% | 67.1% |  |
| Malignant skin melanoma | 0.04 | 0.07 | 0.02 | 0.08 | 0.16 | 0.04 | 133.5% | 358.2% | 20.8% |  |
| Non-melanoma skin cancer | 0.00 | 0.00 | 0.00 | 0.00 | 0.00 | 0.00 | -0.4% | 6.5% | -6.7% |  |
| Ovarian cancer | 0.08 | 0.13 | 0.04 | 0.19 | 0.32 | 0.11 | 141.4% | 345.0% | 39.7% |  |
| Testicular cancer | 0.10 | 0.19 | 0.04 | 0.44 | 0.78 | 0.20 | 351.7% | 1062.3% | 86.2% |  |
| Kidney cancer | 0.08 | 0.12 | 0.04 | 0.15 | 0.24 | 0.09 | 92.5% | 191.8% | 25.8% |  |
| Bladder cancer | 0.15 | 0.25 | 0.09 | 0.18 | 0.27 | 0.11 | 15.8% | 111.4% | -30.8% |  |
| Brain and central nervous system cancer | 0.78 | 1.36 | 0.43 | 1.14 | 1.99 | 0.64 | 45.2% | 171.2% | -21.4% |  |
| Thyroid cancer | 0.14 | 0.22 | 0.07 | 0.28 | 0.49 | 0.16 | 104.2% | 281.7% | 12.8% |  |
| Mesothelioma | 0.00 | 0.00 | 0.00 | 0.00 | 0.00 | 0.00 | 19.0% | 102.1% | -26.5% |  |
| Hodgkin lymphoma | 0.27 | 0.49 | 0.15 | 0.48 | 0.82 | 0.24 | 73.6% | 212.8% | 7.0% |  |
| Non-Hodgkin lymphoma | 0.36 | 0.62 | 0.20 | 0.93 | 1.71 | 0.39 | 156.7% | 425.7% | 19.4% |  |
| Multiple myeloma | 0.00 | 0.01 | 0.00 | 0.01 | 0.03 | 0.01 | 236.5% | 751.2% | 49.2% |  |
| Leukemia | 1.34 | 1.97 | 0.84 | 1.58 | 2.88 | 0.86 | 18.2% | 136.4% | -31.3% |  |
| Eye cancer | 0.01 | 0.01 | 0.00 | 0.01 | 0.02 | 0.00 | 47.7% | 190.7% | -16.5% |  |
| Soft tissue and other extraosseous sarcomas | 0.18 | 0.37 | 0.09 | 0.20 | 0.36 | 0.11 | 11.4% | 90.9% | -39.3% |  |
| Malignant neoplasm of bone and articular cartilage | 0.87 | 1.48 | 0.44 | 0.98 | 1.84 | 0.54 | 12.6% | 152.5% | -43.0% |  |
| Neuroblastoma and other peripheral nervous cell tumors | 0.01 | 0.02 | 0.00 | 0.03 | 0.07 | 0.01 | 216.9% | 714.8% | 33.6% |  |
| Other malignant neoplasms | 0.51 | 0.78 | 0.33 | 0.53 | 0.80 | 0.32 | 4.4% | 71.4% | -37.7% |  |
| Other neoplasms | 0.40 | 0.69 | 0.23 | 0.88 | 1.58 | 0.45 | 122.8% | 276.4% | 44.1% |  |
| **CVDs** | **120.68** | **157.83** | **90.23** | **115.13** | **155.34** | **82.94** | **-4.6%** | **1.5%** | **-10.4%** |  |
| Rheumatic heart disease | 22.11 | 36.42 | 13.72 | 23.93 | 38.70 | 14.42 | 8.2% | 13.0% | 3.2% |  |
| Ischemic heart disease | 8.40 | 12.45 | 5.61 | 7.49 | 10.95 | 4.92 | -10.8% | 0.9% | -20.4% |  |
| Stroke | 68.58 | 88.85 | 49.81 | 55.24 | 70.12 | 39.51 | -19.5% | -13.7% | -24.9% |  |
| Hypertensive heart disease | 0.93 | 1.41 | 0.56 | 1.07 | 1.60 | 0.62 | 14.6% | 36.8% | -5.1% |  |
| Cardiomyopathy and myocarditis | 2.72 | 4.45 | 1.60 | 3.49 | 5.53 | 1.97 | 28.3% | 52.7% | 8.9% |  |
| Pulmonary Arterial Hypertension | 0.08 | 0.13 | 0.05 | 0.08 | 0.12 | 0.05 | -2.6% | 0.8% | -6.1% |  |
| Atrial fibrillation and flutter | 0.00 | 0.00 | 0.00 | 0.00 | 0.00 | 0.00 | 0.0% | 0.0% | 0.0% |  |
| Lower extremity peripheral arterial disease | 0.00 | 0.00 | 0.00 | 0.00 | 0.00 | 0.00 | 0.0% | 0.0% | 0.0% |  |
| Endocarditis | 0.13 | 0.21 | 0.08 | 0.17 | 0.27 | 0.10 | 27.9% | 37.3% | 18.7% |  |
| Non-rheumatic valvular heart disease | 0.05 | 0.07 | 0.03 | 0.08 | 0.12 | 0.05 | 68.9% | 93.7% | 49.9% |  |
| Other cardiovascular and circulatory diseases | 17.68 | 26.12 | 10.79 | 23.59 | 35.46 | 14.06 | 33.4% | 49.3% | 17.3% |  |
| **Chronic respiratory diseases** | **229.37** | **350.62** | **143.29** | **239.17** | **356.61** | **154.54** | **4.3%** | **14.1%** | **-2.2%** |  |
| Chronic obstructive pulmonary disease | 15.01 | 18.07 | 11.94 | 14.80 | 18.11 | 11.30 | -1.4% | 11.6% | -11.9% |  |
| Pneumoconiosis | 0.08 | 0.14 | 0.04 | 0.08 | 0.14 | 0.04 | -2.8% | 10.0% | -18.8% |  |
| Asthma | 209.67 | 329.03 | 124.55 | 217.67 | 334.34 | 133.89 | 3.8% | 15.0% | -3.2% |  |
| Interstitial lung disease and pulmonary sarcoidosis | 0.15 | 0.27 | 0.07 | 0.18 | 0.32 | 0.08 | 22.4% | 35.6% | 14.5% |  |
| Other chronic respiratory diseases | 4.47 | 5.40 | 3.58 | 6.44 | 7.89 | 4.89 | 44.1% | 62.1% | 28.8% |  |
| **Digestive diseases** | **99.77** | **142.12** | **68.69** | **91.31** | **134.02** | **61.71** | **-8.5%** | **-3.9%** | **-13.1%** |  |
| Cirrhosis and other chronic liver diseases | 14.37 | 20.45 | 10.04 | 8.60 | 12.52 | 5.80 | -40.1% | -35.0% | -44.6% |  |
| Upper digestive system diseases | 48.11 | 82.06 | 25.42 | 47.99 | 83.51 | 25.09 | -0.2% | 2.1% | -3.9% |  |
| Appendicitis | 3.87 | 6.59 | 2.19 | 3.74 | 6.52 | 2.06 | -3.2% | 13.1% | -19.5% |  |
| Paralytic ileus and intestinal obstruction | 0.26 | 0.36 | 0.17 | 0.34 | 0.47 | 0.23 | 31.8% | 37.0% | 26.9% |  |
| Inguinal, femoral, and abdominal hernia | 12.85 | 23.18 | 7.01 | 11.21 | 18.50 | 6.18 | -12.8% | 10.6% | -28.3% |  |
| Inflammatory bowel disease | 1.26 | 1.87 | 0.80 | 1.48 | 2.22 | 0.95 | 17.0% | 30.4% | -1.8% |  |
| Vascular intestinal disorders | 0.13 | 0.24 | 0.06 | 0.14 | 0.28 | 0.06 | 13.0% | 19.4% | 7.6% |  |
| Gallbladder and biliary diseases | 15.61 | 24.04 | 8.95 | 14.87 | 23.92 | 8.60 | -4.8% | 4.3% | -12.9% |  |
| Pancreatitis | 1.33 | 2.36 | 0.64 | 1.31 | 2.35 | 0.61 | -1.6% | 1.5% | -4.7% |  |
| Other digestive diseases | 2.00 | 2.73 | 1.43 | 1.64 | 2.25 | 1.11 | -18.3% | -12.6% | -23.6% |  |
| **Neurological disorders** | **785.06** | **1114.10** | **529.25** | **787.34** | **1117.42** | **533.74** | **0.3%** | **8.7%** | **-7.1%** |  |
| Alzheimer's disease and other dementias | 0.00 | 0.00 | 0.00 | 0.00 | 0.00 | 0.00 | 0.0% | 0.0% | 0.0% |  |
| Parkinson's disease | 0.01 | 0.02 | 0.00 | 0.01 | 0.03 | 0.00 | 95.0% | 221.2% | 41.3% |  |
| Idiopathic epilepsy | 122.38 | 197.78 | 61.37 | 96.42 | 169.09 | 46.94 | -21.2% | 22.8% | -50.9% |  |
| Multiple sclerosis | 2.42 | 3.62 | 1.57 | 2.75 | 4.11 | 1.80 | 13.8% | 20.6% | 8.1% |  |
| Motor neuron disease | 0.57 | 0.84 | 0.38 | 0.56 | 0.83 | 0.37 | -1.7% | 1.1% | -4.5% |  |
| Headache disorders | 650.59 | 930.91 | 430.76 | 669.12 | 953.91 | 446.20 | 2.8% | 6.6% | 0.3% |  |
| Other neurological disorders | 9.09 | 14.63 | 4.67 | 18.47 | 32.04 | 9.27 | 103.1% | 211.0% | 27.8% |  |
| **Mental disorders** | **2382.52** | **3295.81** | **1598.88** | **2916.24** | **4165.83** | **2013.40** | **22.4%** | **52.5%** | **-7.0%** |  |
| Schizophrenia | 82.48 | 121.04 | 48.73 | 87.15 | 130.34 | 52.44 | 5.7% | 13.9% | -1.6% |  |
| Depressive disorders | 652.71 | 1006.04 | 427.63 | 944.68 | 1356.17 | 614.61 | 44.7% | 95.1% | 5.0% |  |
| Bipolar disorder | 118.35 | 181.40 | 63.62 | 121.49 | 188.49 | 64.02 | 2.7% | 7.0% | -1.7% |  |
| Anxiety disorders | 965.79 | 1488.83 | 545.43 | 1171.07 | 1951.61 | 650.22 | 21.3% | 98.7% | -30.0% |  |
| Eating disorders | 72.85 | 113.89 | 41.17 | 84.31 | 134.05 | 47.11 | 15.7% | 21.7% | 10.5% |  |
| Autism spectrum disorders | 120.36 | 240.50 | 55.85 | 152.67 | 330.43 | 62.59 | 26.8% | 234.4% | -58.7% |  |
| Attention-deficit/hyperactivity disorder | 115.64 | 175.14 | 68.44 | 110.03 | 166.64 | 65.19 | -4.8% | 1.7% | -9.6% |  |
| Conduct disorder | 177.75 | 298.53 | 100.44 | 169.34 | 281.70 | 96.10 | -4.7% | -1.5% | -7.4% |  |
| Idiopathic developmental intellectual disability | 38.89 | 79.06 | 16.04 | 35.56 | 72.57 | 12.48 | -8.6% | 29.2% | -42.5% |  |
| Other mental disorders | 37.70 | 58.94 | 20.54 | 39.95 | 63.07 | 22.31 | 6.0% | 12.8% | 0.2% |  |
| **Substance use disorders** | **125.08** | **170.02** | **80.16** | **140.05** | **189.05** | **90.68** | **12.0%** | **19.2%** | **5.9%** |  |
| Alcohol use disorders | 25.84 | 45.24 | 13.63 | 25.07 | 43.81 | 13.58 | -3.0% | 5.2% | -10.4% |  |
| Drug use disorders | 99.23 | 136.57 | 65.22 | 114.98 | 155.59 | 75.66 | 15.9% | 25.5% | 8.2% |  |
| **Diabetes and kidney diseases** | **47.65** | **70.10** | **32.24** | **94.55** | **140.03** | **61.92** | **98.4%** | **116.6%** | **79.5%** |  |
| Diabetes mellitus | 36.44 | 55.59 | 23.74 | 83.05 | 127.07 | 53.01 | 127.9% | 149.4% | 107.9% |  |
| Acute glomerulonephritis | 0.02 | 0.03 | 0.01 | 0.02 | 0.04 | 0.01 | 22.4% | 28.1% | 16.6% |  |
| Chronic kidney disease | 11.20 | 16.73 | 7.39 | 11.48 | 16.13 | 8.10 | 2.5% | 20.4% | -16.8% |  |
| **Skin and subcutaneous diseases** | **617.22** | **938.58** | **371.96** | **629.76** | **962.64** | **386.43** | **2.0%** | **6.3%** | **-1.5%** |  |
| Dermatitis | 408.08 | 701.50 | 218.27 | 403.23 | 684.44 | 217.81 | -1.2% | 0.1% | -2.7% |  |
| Psoriasis | 14.13 | 18.74 | 9.69 | 18.67 | 25.04 | 13.18 | 32.1% | 47.8% | 19.7% |  |
| Bacterial skin diseases | 1.45 | 2.73 | 0.73 | 1.47 | 2.78 | 0.73 | 1.0% | 5.1% | -3.4% |  |
| Scabies | 10.04 | 16.77 | 5.65 | 9.84 | 16.55 | 5.64 | -2.0% | 2.7% | -6.6% |  |
| Fungal skin diseases | 44.11 | 101.70 | 16.67 | 24.93 | 56.14 | 9.41 | -43.5% | -36.8% | -49.5% |  |
| Viral skin diseases | 38.11 | 58.00 | 25.06 | 37.84 | 57.11 | 24.85 | -0.7% | 2.3% | -3.7% |  |
| Acne vulgaris | 32.28 | 54.87 | 19.47 | 64.80 | 106.94 | 39.02 | 100.7% | 118.3% | 85.8% |  |
| Alopecia areata | 0.93 | 1.30 | 0.59 | 0.96 | 1.39 | 0.60 | 2.9% | 18.7% | -10.9% |  |
| Pruritus | 0.82 | 1.63 | 0.36 | 0.89 | 1.79 | 0.39 | 8.2% | 17.8% | -0.6% |  |
| Urticaria | 58.08 | 86.04 | 36.08 | 57.48 | 85.41 | 36.21 | -1.0% | 2.8% | -4.8% |  |
| Decubitus ulcer | 0.12 | 0.18 | 0.07 | 0.12 | 0.18 | 0.07 | 0.7% | 3.3% | -3.2% |  |
| Other skin and subcutaneous diseases | 9.07 | 16.86 | 4.42 | 9.55 | 17.73 | 4.61 | 5.2% | 8.0% | 2.1% |  |
| **Sense organ diseases** | **261.12** | **359.17** | **173.72** | **237.57** | **329.29** | **157.04** | **-9.0%** | **-6.2%** | **-12.2%** |  |
| Blindness and vision loss | 68.88 | 99.32 | 47.87 | 54.16 | 79.77 | 36.95 | -21.4% | -15.5% | -27.4% |  |
| Age-related and other hearing loss | 183.35 | 265.25 | 112.21 | 174.31 | 252.13 | 104.81 | -4.9% | -1.6% | -8.2% |  |
| Other sense organ diseases | 8.89 | 14.40 | 4.90 | 9.10 | 14.52 | 4.96 | 2.4% | 9.7% | -3.9% |  |
| **Musculoskeletal disorders** | **760.42** | **1045.81** | **510.59** | **783.40** | **1075.57** | **524.68** | **3.0%** | **6.1%** | **-0.3%** |  |
| Rheumatoid arthritis | 3.14 | 4.85 | 1.79 | 4.59 | 6.90 | 2.77 | 46.4% | 68.4% | 31.7% |  |
| Osteoarthritis | 0.00 | 0.00 | 0.00 | 0.00 | 0.00 | 0.00 | 0.0% | 0.0% | 0.0% |  |
| Low back pain | 588.15 | 823.28 | 401.00 | 578.98 | 824.97 | 393.97 | -1.6% | 1.8% | -4.9% |  |
| Neck pain | 124.97 | 207.54 | 67.13 | 123.81 | 205.36 | 66.21 | -0.9% | 2.3% | -4.4% |  |
| Gout | 0.29 | 0.52 | 0.13 | 0.34 | 0.61 | 0.16 | 18.6% | 27.0% | 13.0% |  |
| Other musculoskeletal disorders | 43.88 | 69.96 | 23.27 | 75.68 | 113.18 | 43.25 | 72.5% | 97.9% | 55.9% |  |
| **Other non-communicable diseases** | **644.02** | **920.54** | **453.65** | **560.11** | **796.52** | **397.94** | **-13.0%** | **-3.1%** | **-21.7%** |  |
| Congenital birth defects | 108.11 | 146.56 | 75.04 | 102.79 | 137.59 | 71.48 | -4.9% | -1.9% | -8.4% |  |
| Urinary diseases and male infertility | 7.74 | 13.75 | 4.49 | 8.53 | 16.25 | 4.56 | 10.1% | 28.1% | -4.6% |  |
| Gynecological diseases | 198.04 | 301.07 | 129.76 | 201.52 | 307.80 | 133.28 | 1.8% | 5.6% | -1.9% |  |
| Hemoglobinopathies and hemolytic anemias | 191.54 | 287.77 | 115.23 | 114.45 | 164.64 | 77.01 | -40.2% | -10.9% | -57.0% |  |
| Endocrine, metabolic, blood, and immune disorders | 46.14 | 78.42 | 26.43 | 41.79 | 70.22 | 23.41 | -9.4% | -3.6% | -16.5% |  |

**Appendix Table 6. Socio-demographic index in 2021 for the 21 MENA countries**

| Country | SDI 2021 | SDI group |
| --- | --- | --- |
| Afghanistan | 0.34 | Low |
| Algeria | 0.66 | Middle |
| Bahrain | 0.75 | High-middle |
| Egypt | 0.60 | Low-middle |
| Iran | 0.70 | Middle |
| Iraq | 0.66 | Middle |
| Jordan | 0.73 | High-middle |
| Kuwait | 0.85 | High |
| Lebanon | 0.74 | High-middle |
| Libya | 0.74 | High-middle |
| Morocco | 0.56 | Low-middle |
| Oman | 0.77 | High-middle |
| Palestine | 0.63 | Middle |
| Qatar | 0.85 | High |
| Saudi Arabia | 0.81 | High |
| Sudan | 0.54 | Low-middle |
| Syria | 0.62 | Middle |
| Tunisia | 0.68 | Middle |
| Turkiye | 0.71 | High-middle |
| UAE | 0.85 | High |
| Yemen | 0.45 | Low |

**Appendix table 7. Spearman rank-correlation coefficients and relative P-values for the association between DALY rates of level 2 NCDs and SDI in 21 MENA countries, both sexes, age 10-24, year 2023**

**
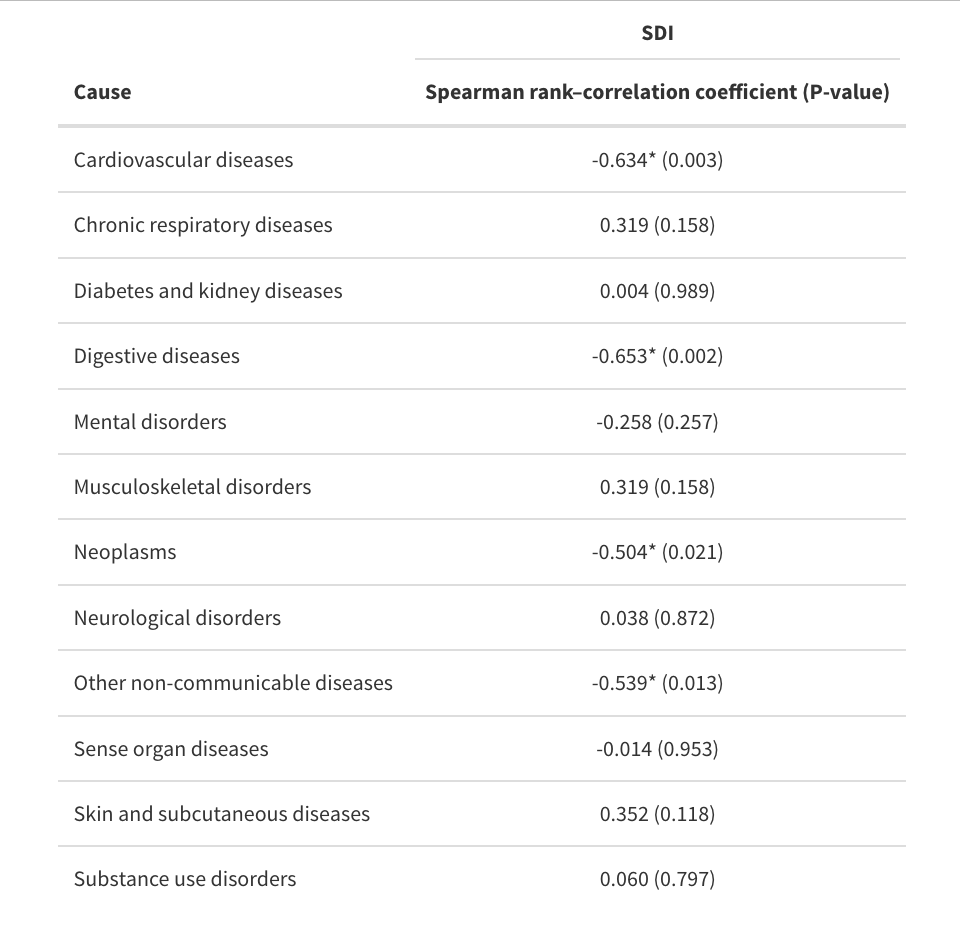
**

*P < 0.05 indicates statistical significance

**Appendix table 8A. Mortality rate per 100 000 population in adolescents aged 10–24 years in MENA countries in 1990, 2000, 2010, 2023, and percentage changes**

|  | **1990** | **2000** | **2010** | **2023** | **1990-2000** | **2000-2010** | **2010-2023** |
| --- | --- | --- | --- | --- | --- | --- | --- |
| Non-communicable diseases | 41.86 (36.42 to 47.29) | 34.80 (31.52 to 38.19) | 29.86 (27.25 to 33.05) | 27.81 (25.33 to 30.75) | -16.85% (-25.01 to -7.06) | -14.21% (-23.98 to -5.17) | -6.85% (-19.15 to 6.97) |
| Neoplasms | 8.74 (7.54 to 9.92) | 7.90 (6.93 to 8.86) | 7.00 (6.18 to 7.79) | 6.93 (6.05 to 7.78) | -9.62% (-19.75 to 2.57) | -11.32% (-20.40 to -1.57) | -0.97% (-14.21 to 11.56) |
| Esophageal cancer | 0.01 (0.01 to 0.02) | 0.01 (0.01 to 0.02) | 0.01 (0.01 to 0.02) | 0.01 (0.01 to 0.02) | -1.91% (-28.57 to 30.77) | 0.13% (-25.60 to 32.82) | -10.64% (-35.68 to 21.35) |
| Stomach cancer | 0.22 (0.15 to 0.31) | 0.18 (0.13 to 0.23) | 0.15 (0.11 to 0.19) | 0.13 (0.09 to 0.16) | -20.14% (-40.56 to 7.81) | -17.05% (-36.49 to 10.84) | -11.87% (-41.03 to 23.13) |
| Liver cancer | 0.22 (0.14 to 0.31) | 0.18 (0.12 to 0.24) | 0.17 (0.12 to 0.23) | 0.21 (0.14 to 0.29) | -20.04% (-46.12 to 29.07) | -4.42% (-35.87 to 51.23) | 22.22% (-25.11 to 99.75) |
| Larynx cancer | 0.02 (0.01 to 0.02) | 0.02 (0.01 to 0.02) | 0.02 (0.01 to 0.02) | 0.02 (0.01 to 0.02) | -2.39% (-28.63 to 31.44) | 1.00% (-25.90 to 34.51) | 1.76% (-27.09 to 40.04) |
| Tracheal, bronchus, and lung cancer | 0.20 (0.14 to 0.27) | 0.21 (0.16 to 0.28) | 0.22 (0.17 to 0.29) | 0.26 (0.19 to 0.34) | 7.72% (-21.65 to 48.41) | 4.49% (-22.29 to 43.13) | 15.78% (-18.87 to 65.83) |
| Breast cancer | 0.06 (0.04 to 0.08) | 0.07 (0.05 to 0.09) | 0.08 (0.06 to 0.11) | 0.11 (0.08 to 0.15) | 16.03% (-22.60 to 67.08) | 15.15% (-25.07 to 78.24) | 34.78% (-10.98 to 108.42) |
| Cervical cancer | 0.07 (0.03 to 0.11) | 0.06 (0.03 to 0.09) | 0.05 (0.03 to 0.08) | 0.05 (0.03 to 0.07) | -15.04% (-46.70 to 37.25) | -9.20% (-45.28 to 46.37) | -10.66% (-46.56 to 46.59) |
| Uterine cancer | 0.01 (0.00 to 0.01) | 0.01 (0.00 to 0.01) | 0.01 (0.00 to 0.01) | 0.01 (0.01 to 0.01) | 4.03% (-29.52 to 58.70) | 8.20% (-28.24 to 86.03) | 21.67% (-22.39 to 97.51) |
| Prostate cancer | 0.00 (0.00 to 0.01) | 0.00 (0.00 to 0.01) | 0.01 (0.00 to 0.01) | 0.01 (0.00 to 0.01) | 4.08% (-24.95 to 61.10) | 21.56% (-16.97 to 78.06) | 4.54% (-32.90 to 52.90) |
| Colon and rectum cancer | 0.15 (0.11 to 0.21) | 0.15 (0.12 to 0.19) | 0.17 (0.13 to 0.21) | 0.19 (0.14 to 0.25) | 1.22% (-22.40 to 33.00) | 10.02% (-15.80 to 61.00) | 14.81% (-18.85 to 59.62) |
| Lip and oral cavity cancer | 0.02 (0.02 to 0.03) | 0.02 (0.02 to 0.03) | 0.03 (0.02 to 0.03) | 0.03 (0.02 to 0.03) | -1.48% (-31.46 to 35.20) | 9.04% (-18.01 to 49.26) | 4.18% (-24.44 to 44.83) |
| Nasopharynx cancer | 0.09 (0.06 to 0.12) | 0.06 (0.05 to 0.09) | 0.05 (0.04 to 0.07) | 0.05 (0.03 to 0.06) | -26.22% (-48.86 to 7.00) | -15.99% (-41.43 to 26.23) | -13.39% (-44.29 to 27.73) |
| Other pharynx cancer | 0.00 (0.00 to 0.01) | 0.00 (0.00 to 0.01) | 0.01 (0.00 to 0.01) | 0.01 (0.00 to 0.01) | 3.66% (-24.00 to 44.09) | 25.58% (-3.69 to 66.79) | 3.74% (-26.05 to 46.30) |
| Gallbladder and biliary tract cancer | 0.01 (0.01 to 0.01) | 0.01 (0.01 to 0.01) | 0.01 (0.01 to 0.01) | 0.01 (0.01 to 0.01) | -4.32% (-28.32 to 26.04) | 11.79% (-15.14 to 45.42) | -3.35% (-28.45 to 24.14) |
| Pancreatic cancer | 0.02 (0.01 to 0.02) | 0.02 (0.02 to 0.03) | 0.03 (0.02 to 0.03) | 0.03 (0.02 to 0.04) | 9.63% (-15.22 to 46.01) | 23.28% (-7.64 to 65.82) | 29.35% (-7.75 to 76.86) |
| Malignant skin melanoma | 0.01 (0.01 to 0.02) | 0.01 (0.01 to 0.02) | 0.01 (0.01 to 0.02) | 0.01 (0.01 to 0.02) | 8.87% (-23.09 to 55.87) | -11.07% (-40.83 to 20.45) | 3.19% (-33.92 to 46.37) |
| Non-melanoma skin cancer | 0.01 (0.00 to 0.01) | 0.01 (0.00 to 0.01) | 0.01 (0.01 to 0.01) | 0.01 (0.01 to 0.02) | 7.86% (-23.52 to 52.73) | 36.01% (-0.96 to 97.62) | 17.74% (-23.67 to 76.84) |
| Ovarian cancer | 0.04 (0.03 to 0.07) | 0.05 (0.04 to 0.08) | 0.06 (0.04 to 0.07) | 0.07 (0.05 to 0.10) | 24.25% (-18.77 to 87.52) | 0.97% (-31.64 to 47.14) | 30.26% (-15.16 to 110.94) |
| Testicular cancer | 0.05 (0.03 to 0.08) | 0.05 (0.03 to 0.08) | 0.05 (0.03 to 0.07) | 0.05 (0.04 to 0.08) | -6.24% (-42.24 to 58.51) | -2.68% (-32.88 to 51.29) | 13.02% (-34.21 to 81.01) |
| Kidney cancer | 0.06 (0.04 to 0.08) | 0.06 (0.04 to 0.08) | 0.05 (0.04 to 0.07) | 0.06 (0.04 to 0.08) | -3.88% (-28.32 to 29.47) | -5.82% (-29.28 to 29.43) | 8.17% (-22.14 to 51.75) |
| Bladder cancer | 0.03 (0.02 to 0.04) | 0.02 (0.02 to 0.03) | 0.02 (0.01 to 0.03) | 0.02 (0.01 to 0.03) | -12.61% (-39.85 to 18.99) | -23.22% (-45.62 to 11.60) | 10.97% (-25.76 to 64.84) |
| Brain and central nervous system cancer | 1.27 (0.89 to 1.69) | 1.22 (0.88 to 1.55) | 1.15 (0.85 to 1.44) | 1.21 (0.90 to 1.53) | -4.58% (-21.93 to 16.70) | -5.21% (-20.39 to 24.66) | 4.94% (-15.93 to 38.17) |
| Thyroid cancer | 0.03 (0.02 to 0.04) | 0.03 (0.02 to 0.04) | 0.03 (0.02 to 0.04) | 0.03 (0.02 to 0.04) | -10.28% (-35.67 to 36.14) | 3.66% (-26.45 to 49.00) | -0.37% (-30.17 to 40.11) |
| Mesothelioma | 0.01 (0.00 to 0.01) | 0.01 (0.00 to 0.01) | 0.01 (0.01 to 0.01) | 0.01 (0.00 to 0.01) | 4.80% (-32.44 to 63.54) | 11.27% (-25.24 to 58.65) | 3.17% (-32.62 to 57.03) |
| Hodgkin lymphoma | 0.29 (0.16 to 0.43) | 0.22 (0.13 to 0.33) | 0.18 (0.10 to 0.26) | 0.15 (0.08 to 0.22) | -23.09% (-47.51 to 16.91) | -21.50% (-43.16 to 15.57) | -16.15% (-46.20 to 30.51) |
| Non-Hodgkin lymphoma | 0.58 (0.43 to 0.76) | 0.53 (0.42 to 0.68) | 0.47 (0.37 to 0.58) | 0.50 (0.38 to 0.66) | -9.09% (-31.00 to 21.43) | -11.00% (-34.74 to 20.16) | 7.23% (-19.01 to 41.95) |
| Multiple myeloma | 0.01 (0.01 to 0.01) | 0.01 (0.01 to 0.01) | 0.01 (0.01 to 0.02) | 0.02 (0.01 to 0.02) | 13.64% (-18.43 to 53.69) | 23.02% (-9.20 to 79.85) | 21.25% (-18.01 to 82.53) |
| Leukemia | 3.25 (2.51 to 4.18) | 2.88 (2.23 to 3.57) | 2.43 (1.96 to 2.92) | 2.24 (1.81 to 2.74) | -11.29% (-29.45 to 9.25) | -15.75% (-28.34 to -0.87) | -7.74% (-28.38 to 16.96) |
| Other malignant neoplasms | 0.86 (0.65 to 1.14) | 0.74 (0.57 to 0.93) | 0.61 (0.48 to 0.78) | 0.54 (0.41 to 0.69) | -13.65% (-36.28 to 21.51) | -18.18% (-38.01 to 9.31) | -11.28% (-38.80 to 22.15) |
| Other neoplasms | 0.12 (0.07 to 0.19) | 0.11 (0.06 to 0.17) | 0.09 (0.05 to 0.14) | 0.09 (0.06 to 0.14) | -7.81% (-33.59 to 28.22) | -19.88% (-39.90 to 8.12) | 4.44% (-31.86 to 48.97) |
| Cardiovascular diseases | 17.10 (12.88 to 21.48) | 13.42 (10.50 to 16.37) | 10.89 (8.74 to 13.62) | 9.64 (7.64 to 12.14) | -21.50% (-36.98 to 0.32) | -18.87% (-36.30 to 3.15) | -11.45% (-35.05 to 22.63) |
| Rheumatic heart disease | 2.48 (1.40 to 3.50) | 1.54 (1.01 to 2.26) | 0.93 (0.59 to 1.44) | 0.74 (0.43 to 1.19) | -38.09% (-61.47 to -6.15) | -39.22% (-60.95 to -8.20) | -21.04% (-55.83 to 35.02) |
| Ischemic heart disease | 4.82 (3.38 to 6.71) | 4.11 (3.04 to 5.41) | 3.68 (2.77 to 4.81) | 3.52 (2.63 to 4.68) | -14.76% (-37.15 to 24.32) | -10.38% (-33.99 to 28.57) | -4.38% (-33.17 to 34.88) |
| Stroke | 6.58 (4.55 to 8.99) | 4.98 (3.59 to 6.70) | 3.91 (2.89 to 5.25) | 3.07 (2.13 to 4.25) | -24.37% (-43.66 to 6.79) | -21.39% (-42.29 to 3.85) | -21.61% (-49.27 to 19.64) |
| Hypertensive heart disease | 0.41 (0.23 to 0.66) | 0.36 (0.21 to 0.55) | 0.35 (0.24 to 0.53) | 0.33 (0.22 to 0.49) | -13.25% (-46.37 to 32.18) | -3.41% (-38.58 to 50.13) | -3.67% (-42.67 to 44.78) |
| Cardiomyopathy and myocarditis | 0.69 (0.38 to 1.16) | 0.59 (0.34 to 0.98) | 0.50 (0.31 to 0.79) | 0.46 (0.28 to 0.78) | -15.11% (-46.11 to 38.19) | -15.27% (-44.78 to 35.56) | -7.98% (-46.80 to 53.33) |
| Aortic aneurysm | 0.02 (0.01 to 0.03) | 0.02 (0.01 to 0.03) | 0.03 (0.02 to 0.04) | 0.03 (0.02 to 0.05) | 8.40% (-29.09 to 73.13) | 38.98% (-9.66 to 104.85) | 16.57% (-32.87 to 105.39) |
| Endocarditis | 0.15 (0.10 to 0.25) | 0.12 (0.08 to 0.18) | 0.10 (0.07 to 0.16) | 0.10 (0.07 to 0.15) | -20.96% (-51.96 to 25.93) | -13.14% (-45.28 to 44.94) | -7.29% (-47.05 to 65.33) |
| Non-rheumatic valvular heart disease | 0.21 (0.12 to 0.33) | 0.21 (0.12 to 0.34) | 0.21 (0.14 to 0.32) | 0.21 (0.13 to 0.32) | -0.83% (-39.51 to 57.83) | -2.59% (-39.25 to 46.70) | 1.17% (-36.91 to 68.08) |
| Other cardiovascular and circulatory diseases | 1.53 (0.90 to 2.36) | 1.36 (0.85 to 2.01) | 1.07 (0.70 to 1.52) | 1.10 (0.76 to 1.50) | -10.89% (-36.90 to 35.37) | -21.54% (-47.47 to 12.83) | 2.78% (-35.61 to 68.57) |
| Chronic respiratory diseases | 1.31 (0.77 to 1.90) | 1.11 (0.74 to 1.56) | 0.96 (0.72 to 1.28) | 0.82 (0.61 to 1.10) | -15.10% (-39.33 to 17.35) | -13.63% (-36.06 to 16.90) | -14.17% (-42.97 to 25.71) |
| Chronic obstructive pulmonary disease | 0.41 (0.20 to 0.65) | 0.37 (0.23 to 0.54) | 0.34 (0.24 to 0.48) | 0.30 (0.20 to 0.41) | -10.18% (-40.53 to 45.02) | -6.94% (-40.04 to 33.00) | -12.16% (-45.93 to 38.45) |
| Pneumoconiosis | 0.01 (0.00 to 0.02) | 0.01 (0.00 to 0.01) | 0.01 (0.00 to 0.02) | 0.01 (0.00 to 0.02) | -8.09% (-55.83 to 83.64) | 8.25% (-41.19 to 97.83) | 2.81% (-52.69 to 109.61) |
| Asthma | 0.65 (0.36 to 1.02) | 0.51 (0.29 to 0.76) | 0.39 (0.27 to 0.56) | 0.29 (0.19 to 0.43) | -20.71% (-45.72 to 14.01) | -24.23% (-46.72 to 10.15) | -26.49% (-56.08 to 14.60) |
| Interstitial lung disease and pulmonary sarcoidosis | 0.04 (0.02 to 0.08) | 0.04 (0.02 to 0.08) | 0.05 (0.03 to 0.08) | 0.05 (0.03 to 0.08) | 4.82% (-40.40 to 82.15) | 16.07% (-32.04 to 94.90) | 1.58% (-49.91 to 71.69) |
| Other chronic respiratory diseases | 0.20 (0.11 to 0.36) | 0.18 (0.10 to 0.30) | 0.17 (0.11 to 0.26) | 0.18 (0.11 to 0.27) | -11.47% (-45.70 to 35.78) | -5.10% (-39.81 to 49.38) | 4.46% (-40.67 to 77.27) |
| Cirrhosis and other chronic liver diseases | 2.00 (1.47 to 2.57) | 1.46 (1.12 to 1.89) | 1.13 (0.85 to 1.48) | 0.75 (0.57 to 0.96) | -26.76% (-44.44 to -7.24) | -22.89% (-41.37 to 2.64) | -33.55% (-54.34 to -2.81) |
| Digestive diseases | 2.89 (2.19 to 3.63) | 2.11 (1.66 to 2.70) | 1.56 (1.21 to 2.03) | 1.13 (0.87 to 1.43) | -26.89% (-42.01 to -8.90) | -25.89% (-42.47 to -2.46) | -27.78% (-49.49 to 2.91) |
| Appendicitis | 0.13 (0.05 to 0.21) | 0.10 (0.05 to 0.17) | 0.06 (0.03 to 0.09) | 0.04 (0.02 to 0.06) | -23.91% (-56.73 to 36.73) | -40.00% (-63.37 to 3.01) | -30.12% (-63.10 to 30.13) |
| Paralytic ileus and intestinal obstruction | 0.15 (0.10 to 0.23) | 0.12 (0.07 to 0.19) | 0.09 (0.06 to 0.14) | 0.08 (0.05 to 0.13) | -25.63% (-54.20 to 20.36) | -24.94% (-53.91 to 21.90) | -3.89% (-43.65 to 62.68) |
| Inguinal, femoral, and abdominal hernia | 0.01 (0.00 to 0.02) | 0.01 (0.00 to 0.01) | 0.00 (0.00 to 0.01) | 0.00 (0.00 to 0.01) | -28.29% (-60.74 to 49.70) | -33.73% (-61.26 to 13.71) | -20.11% (-59.89 to 77.94) |
| Inflammatory bowel disease | 0.05 (0.03 to 0.09) | 0.05 (0.03 to 0.08) | 0.04 (0.02 to 0.06) | 0.04 (0.02 to 0.06) | -11.27% (-43.29 to 48.23) | -20.20% (-49.30 to 27.33) | 3.36% (-40.17 to 83.16) |
| Vascular intestinal disorders | 0.02 (0.01 to 0.02) | 0.01 (0.01 to 0.02) | 0.01 (0.01 to 0.02) | 0.01 (0.01 to 0.02) | -13.12% (-49.81 to 55.87) | -23.14% (-55.39 to 29.40) | 8.42% (-35.45 to 110.56) |
| Gallbladder and biliary diseases | 0.03 (0.02 to 0.05) | 0.03 (0.02 to 0.04) | 0.02 (0.01 to 0.03) | 0.02 (0.01 to 0.04) | -0.65% (-37.69 to 56.71) | -24.00% (-51.55 to 25.06) | 13.44% (-40.31 to 88.03) |
| Pancreatitis | 0.04 (0.02 to 0.08) | 0.04 (0.02 to 0.06) | 0.03 (0.02 to 0.05) | 0.03 (0.02 to 0.04) | -16.57% (-44.64 to 26.75) | -18.00% (-48.14 to 35.26) | -9.11% (-44.46 to 58.87) |
| Neurological disorders | 1.54 (0.98 to 2.08) | 1.32 (0.99 to 1.69) | 1.24 (1.00 to 1.52) | 1.43 (1.16 to 1.76) | -14.31% (-32.97 to 9.76) | -5.84% (-25.63 to 21.44) | 15.16% (-7.27 to 47.20) |
| Parkinson's disease | 0.00 (0.00 to 0.00) | 0.00 (0.00 to 0.00) | 0.00 (0.00 to 0.00) | 0.00 (0.00 to 0.00) | 1.09% (-24.26 to 24.92) | 12.74% (-9.26 to 41.52) | 10.02% (-15.12 to 51.16) |
| Idiopathic epilepsy | 1.43 (0.86 to 1.96) | 1.18 (0.84 to 1.55) | 1.02 (0.77 to 1.26) | 0.97 (0.73 to 1.26) | -17.24% (-35.80 to 6.85) | -13.79% (-32.36 to 12.31) | -4.79% (-26.22 to 31.05) |
| Multiple sclerosis | 0.02 (0.01 to 0.04) | 0.02 (0.01 to 0.04) | 0.02 (0.01 to 0.04) | 0.02 (0.01 to 0.04) | 13.10% (-46.94 to 144.49) | 6.26% (-48.86 to 135.38) | 11.30% (-55.71 to 164.95) |
| Motor neuron disease | 0.01 (0.01 to 0.02) | 0.01 (0.01 to 0.03) | 0.02 (0.01 to 0.04) | 0.04 (0.02 to 0.08) | 28.32% (-25.95 to 158.22) | 63.33% (0.41 to 196.41) | 85.54% (-10.20 to 230.52) |
| Other neurological disorders | 0.09 (0.05 to 0.15) | 0.11 (0.07 to 0.16) | 0.18 (0.12 to 0.25) | 0.40 (0.30 to 0.53) | 22.31% (-28.49 to 102.30) | 70.10% (12.01 to 153.09) | 117.98% (53.87 to 216.79) |
| Mental disorders | 0.00 (0.00 to 0.00) | 0.00 (0.00 to 0.00) | 0.00 (0.00 to 0.00) | 0.00 (0.00 to 0.00) | 44.41% (-73.70 to 721.39) | 26.74% (-64.53 to 352.68) | 24.80% (-73.89 to 511.32) |
| Alcohol use disorders | 0.08 (0.05 to 0.12) | 0.07 (0.05 to 0.11) | 0.06 (0.04 to 0.08) | 0.05 (0.03 to 0.07) | -14.01% (-44.30 to 34.90) | -22.59% (-50.87 to 17.86) | -16.85% (-52.15 to 40.61) |
| Drug use disorders | 0.56 (0.37 to 0.81) | 0.66 (0.45 to 0.94) | 0.73 (0.53 to 1.04) | 0.68 (0.47 to 0.99) | 18.53% (-20.50 to 85.55) | 11.27% (-18.15 to 78.76) | -7.79% (-44.38 to 51.12) |
| Eating disorders | 0.00 (0.00 to 0.00) | 0.00 (0.00 to 0.00) | 0.00 (0.00 to 0.00) | 0.00 (0.00 to 0.00) | 44.41% (-73.70 to 721.39) | 26.74% (-64.53 to 352.68) | 24.80% (-73.89 to 511.32) |
| Diabetes mellitus | 0.62 (0.44 to 0.88) | 0.53 (0.38 to 0.73) | 0.52 (0.41 to 0.65) | 0.50 (0.39 to 0.65) | -14.80% (-32.09 to 9.00) | -0.55% (-19.23 to 30.14) | -3.46% (-24.47 to 26.53) |
| Acute glomerulonephritis | 0.02 (0.01 to 0.03) | 0.01 (0.00 to 0.02) | 0.01 (0.01 to 0.02) | 0.01 (0.01 to 0.02) | -36.76% (-67.61 to 13.61) | 5.18% (-38.76 to 125.17) | -5.81% (-53.55 to 78.97) |
| Chronic kidney disease | 1.87 (1.32 to 2.60) | 1.54 (1.12 to 2.03) | 1.45 (1.15 to 1.88) | 1.67 (1.24 to 2.12) | -17.75% (-34.97 to 11.30) | -6.04% (-29.47 to 21.40) | 15.63% (-21.65 to 58.07) |
| Urinary diseases and male infertility | 0.15 (0.12 to 0.19) | 0.12 (0.10 to 0.14) | 0.11 (0.09 to 0.13) | 0.10 (0.08 to 0.12) | -20.23% (-34.87 to -6.30) | -10.03% (-24.53 to 12.21) | -5.89% (-25.13 to 21.91) |
| Gynecological diseases | 0.02 (0.01 to 0.03) | 0.01 (0.01 to 0.03) | 0.01 (0.01 to 0.02) | 0.01 (0.01 to 0.02) | -14.22% (-56.41 to 83.61) | -24.42% (-62.83 to 40.72) | 6.73% (-55.63 to 141.32) |
| Hemoglobinopathies and hemolytic anemias | 1.46 (1.14 to 1.89) | 1.18 (0.92 to 1.53) | 0.96 (0.75 to 1.25) | 0.73 (0.53 to 0.94) | -19.36% (-40.86 to 11.24) | -18.26% (-38.69 to 9.21) | -24.57% (-49.11 to 5.72) |
| Endocrine, metabolic, blood, and immune disorders | 0.49 (0.32 to 0.69) | 0.44 (0.31 to 0.62) | 0.48 (0.35 to 0.66) | 0.62 (0.43 to 0.85) | -8.70% (-43.30 to 32.90) | 7.35% (-22.37 to 54.32) | 31.27% (-12.59 to 86.71) |
| Musculoskeletal disorders | 0.24 (0.15 to 0.36) | 0.23 (0.15 to 0.34) | 0.22 (0.15 to 0.31) | 0.21 (0.14 to 0.28) | -5.28% (-35.08 to 41.35) | -5.49% (-33.83 to 37.73) | -5.81% (-38.33 to 36.66) |
| Rheumatoid arthritis | 0.01 (0.00 to 0.02) | 0.01 (0.00 to 0.02) | 0.01 (0.00 to 0.01) | 0.01 (0.00 to 0.01) | -20.32% (-58.41 to 51.91) | -20.78% (-56.36 to 49.96) | -9.01% (-54.17 to 72.88) |
| Other musculoskeletal disorders | 0.23 (0.14 to 0.35) | 0.22 (0.14 to 0.32) | 0.21 (0.14 to 0.30) | 0.20 (0.13 to 0.27) | -4.59% (-34.92 to 43.22) | -4.91% (-33.86 to 38.16) | -5.70% (-38.08 to 36.61) |
| Other non-communicable diseases | 6.86 (5.58 to 8.46) | 5.87 (4.94 to 6.91) | 5.18 (4.45 to 6.05) | 4.70 (3.87 to 5.70) | -14.41% (-31.60 to 2.69) | -11.86% (-27.74 to 5.67) | -9.17% (-29.66 to 13.44) |
| Congenital birth defects | 4.75 (3.50 to 6.28) | 4.12 (3.21 to 5.08) | 3.62 (2.91 to 4.55) | 3.24 (2.44 to 4.17) | -13.28% (-35.69 to 11.06) | -12.11% (-31.95 to 11.49) | -10.53% (-35.62 to 19.43) |
| Skin and subcutaneous diseases | 0.04 (0.02 to 0.07) | 0.04 (0.02 to 0.06) | 0.04 (0.03 to 0.06) | 0.04 (0.02 to 0.06) | -11.29% (-43.65 to 48.27) | 4.70% (-36.71 to 58.54) | -2.50% (-50.97 to 78.32) |
| Decubitus ulcer | 0.03 (0.01 to 0.05) | 0.02 (0.01 to 0.04) | 0.02 (0.01 to 0.04) | 0.02 (0.01 to 0.04) | -14.29% (-48.04 to 49.22) | 2.29% (-39.56 to 70.13) | -11.19% (-55.57 to 71.96) |
| Other skin and subcutaneous diseases | 0.01 (0.00 to 0.02) | 0.01 (0.00 to 0.02) | 0.01 (0.00 to 0.02) | 0.01 (0.00 to 0.02) | -18.33% (-59.03 to 58.67) | -3.95% (-50.23 to 83.20) | 8.43% (-50.15 to 134.98) |
| Substance use disorders | 0.64 (0.45 to 0.89) | 0.73 (0.51 to 1.03) | 0.79 (0.58 to 1.10) | 0.72 (0.52 to 1.04) | 14.25% (-20.59 to 70.58) | 7.92% (-20.33 to 68.14) | -8.43% (-43.40 to 45.68) |
| Diabetes and kidney diseases | 2.50 (1.91 to 3.25) | 2.07 (1.60 to 2.61) | 1.98 (1.61 to 2.42) | 2.18 (1.70 to 2.68) | -17.14% (-30.90 to 1.55) | -4.60% (-23.37 to 18.50) | 10.48% (-16.22 to 44.14) |
| Bacterial skin diseases | 0.01 (0.00 to 0.02) | 0.01 (0.00 to 0.01) | 0.01 (0.00 to 0.02) | 0.01 (0.00 to 0.02) | 16.65% (-65.57 to 606.05) | 24.80% (-50.21 to 283.53) | 12.00% (-55.18 to 159.10) |
| Upper digestive system diseases | 0.38 (0.25 to 0.60) | 0.25 (0.15 to 0.36) | 0.14 (0.09 to 0.22) | 0.09 (0.06 to 0.16) | -35.80% (-60.25 to -0.50) | -43.13% (-63.55 to -4.23) | -34.04% (-60.31 to 11.34) |
| Pulmonary Arterial Hypertension | 0.19 (0.09 to 0.33) | 0.13 (0.07 to 0.23) | 0.10 (0.06 to 0.17) | 0.08 (0.05 to 0.14) | -29.85% (-59.09 to 35.57) | -22.48% (-56.19 to 38.15) | -19.82% (-62.80 to 50.58) |
| Eye cancer | 0.00 (0.00 to 0.00) | 0.00 (0.00 to 0.00) | 0.00 (0.00 to 0.00) | 0.00 (0.00 to 0.00) | -13.86% (-43.44 to 26.04) | 0.35% (-39.59 to 50.89) | 5.49% (-34.77 to 62.24) |
| Soft tissue and other extraosseous sarcomas | 0.16 (0.10 to 0.27) | 0.14 (0.09 to 0.21) | 0.12 (0.08 to 0.17) | 0.12 (0.08 to 0.18) | -12.66% (-45.74 to 40.61) | -18.46% (-48.18 to 27.00) | 5.47% (-32.18 to 59.90) |
| Malignant neoplasm of bone and articular cartilage | 0.81 (0.53 to 1.33) | 0.76 (0.53 to 1.12) | 0.69 (0.49 to 0.91) | 0.66 (0.48 to 0.94) | -6.63% (-40.46 to 39.67) | -9.72% (-34.81 to 30.72) | -4.19% (-38.66 to 45.04) |
| Neuroblastoma and other peripheral nervous cell tumors | 0.02 (0.01 to 0.03) | 0.02 (0.01 to 0.03) | 0.02 (0.01 to 0.03) | 0.02 (0.02 to 0.04) | 0.44% (-28.94 to 40.42) | -3.48% (-27.24 to 39.30) | 25.55% (-19.88 to 89.77) |
| Other digestive diseases | 0.07 (0.04 to 0.11) | 0.06 (0.03 to 0.09) | 0.05 (0.03 to 0.07) | 0.06 (0.03 to 0.09) | -21.71% (-52.30 to 28.50) | -16.85% (-46.85 to 30.35) | 24.94% (-30.74 to 130.39) |

**Appendix table 8B. DALY rate per 100 000 population in adolescents aged 10–24 years in MENA countries in 1990, 2000, 2010, 2023, and percentage changes**

|  | **1990** | **2000** | **2010** | **2023** | **1990-2000** | **2000-2010** | **2010-2023** |
| --- | --- | --- | --- | --- | --- | --- | --- |
| Non-communicable diseases | 9128.65 (7381.00 to 10894.37) | 8637.13 (6977.74 to 10630.15) | 8279.77 (6603.86 to 10229.17) | 8607.79 (6747.88 to 10602.02) | -5.38% (-10.39 to -0.31) | -4.14% (-9.03 to 0.38) | 3.96% (-2.40 to 14.17) |
| Neoplasms | 644.51 (556.37 to 731.96) | 580.69 (509.14 to 650.86) | 512.91 (450.63 to 570.73) | 510.72 (446.87 to 574.20) | -9.90% (-19.92 to 2.43) | -11.67% (-20.74 to -1.87) | -0.43% (-13.49 to 11.98) |
| Esophageal cancer | 0.90 (0.61 to 1.20) | 0.88 (0.62 to 1.12) | 0.88 (0.66 to 1.12) | 0.79 (0.59 to 1.04) | -1.82% (-28.45 to 30.82) | 0.09% (-25.66 to 32.79) | -10.42% (-35.50 to 21.60) |
| Stomach cancer | 15.74 (10.38 to 21.87) | 12.56 (9.31 to 16.23) | 10.38 (7.42 to 13.65) | 9.19 (6.18 to 11.64) | -20.18% (-40.61 to 7.84) | -17.36% (-36.73 to 10.64) | -11.48% (-40.75 to 23.43) |
| Liver cancer | 16.24 (10.34 to 22.19) | 12.94 (8.94 to 17.74) | 12.25 (8.68 to 16.63) | 14.98 (10.08 to 21.08) | -20.29% (-46.27 to 28.91) | -5.37% (-36.35 to 49.73) | 22.28% (-25.18 to 99.60) |
| Larynx cancer | 1.19 (0.80 to 1.68) | 1.16 (0.82 to 1.59) | 1.17 (0.86 to 1.53) | 1.19 (0.88 to 1.57) | -2.32% (-28.64 to 31.53) | 0.96% (-25.89 to 34.41) | 1.95% (-27.03 to 40.26) |
| Tracheal, bronchus, and lung cancer | 14.00 (10.11 to 18.87) | 15.05 (11.22 to 19.69) | 15.65 (11.99 to 20.08) | 18.13 (13.47 to 23.86) | 7.50% (-21.83 to 48.19) | 4.02% (-22.66 to 42.56) | 15.79% (-18.86 to 66.01) |
| Breast cancer | 4.43 (3.17 to 5.96) | 5.18 (3.78 to 6.75) | 5.99 (4.38 to 8.05) | 8.16 (5.56 to 11.20) | 16.83% (-22.32 to 68.54) | 15.76% (-24.58 to 78.66) | 36.21% (-9.76 to 110.15) |
| Cervical cancer | 4.85 (2.49 to 8.13) | 4.13 (2.48 to 6.42) | 3.75 (2.31 to 5.66) | 3.37 (1.96 to 5.40) | -14.82% (-46.60 to 36.70) | -9.17% (-45.00 to 46.75) | -10.19% (-46.31 to 47.26) |
| Uterine cancer | 0.40 (0.25 to 0.59) | 0.42 (0.28 to 0.63) | 0.46 (0.31 to 0.66) | 0.57 (0.36 to 0.86) | 4.63% (-29.14 to 59.22) | 8.87% (-27.74 to 86.90) | 22.63% (-22.07 to 100.54) |
| Prostate cancer | 0.30 (0.20 to 0.45) | 0.32 (0.21 to 0.46) | 0.39 (0.26 to 0.57) | 0.42 (0.27 to 0.63) | 5.29% (-24.37 to 62.84) | 23.55% (-14.85 to 80.92) | 7.10% (-31.49 to 58.97) |
| Colon and rectum cancer | 10.71 (7.96 to 14.94) | 10.86 (8.26 to 13.71) | 11.96 (9.23 to 15.16) | 13.84 (10.04 to 17.93) | 1.39% (-22.18 to 33.40) | 10.06% (-15.66 to 61.30) | 15.72% (-17.85 to 60.60) |
| Lip and oral cavity cancer | 1.69 (1.24 to 2.31) | 1.66 (1.24 to 2.13) | 1.81 (1.37 to 2.30) | 1.90 (1.41 to 2.48) | -1.31% (-31.38 to 35.64) | 8.82% (-18.34 to 49.28) | 4.78% (-24.20 to 45.89) |
| Nasopharynx cancer | 6.42 (4.26 to 9.09) | 4.74 (3.44 to 6.31) | 3.99 (2.98 to 5.40) | 3.53 (2.39 to 4.95) | -26.14% (-48.72 to 7.15) | -15.91% (-41.30 to 27.16) | -11.37% (-43.57 to 32.35) |
| Other pharynx cancer | 0.28 (0.19 to 0.38) | 0.29 (0.22 to 0.40) | 0.37 (0.27 to 0.49) | 0.38 (0.27 to 0.53) | 3.68% (-23.93 to 44.11) | 25.51% (-3.76 to 66.75) | 3.86% (-26.02 to 46.41) |
| Gallbladder and biliary tract cancer | 0.59 (0.43 to 0.74) | 0.56 (0.41 to 0.77) | 0.63 (0.48 to 0.79) | 0.61 (0.45 to 0.79) | -4.24% (-28.24 to 26.12) | 11.80% (-15.19 to 45.42) | -3.03% (-28.08 to 24.48) |
| Pancreatic cancer | 1.31 (0.93 to 1.69) | 1.43 (1.06 to 1.82) | 1.76 (1.41 to 2.22) | 2.28 (1.71 to 2.98) | 9.40% (-15.39 to 45.61) | 22.80% (-7.94 to 65.27) | 29.49% (-7.51 to 76.87) |
| Malignant skin melanoma | 0.99 (0.55 to 1.42) | 1.09 (0.66 to 1.59) | 0.97 (0.61 to 1.36) | 1.03 (0.66 to 1.42) | 9.98% (-22.16 to 56.74) | -10.46% (-40.67 to 21.33) | 5.51% (-33.08 to 49.79) |
| Non-melanoma skin cancer | 0.42 (0.29 to 0.61) | 0.45 (0.32 to 0.63) | 0.62 (0.41 to 0.87) | 0.72 (0.49 to 1.08) | 7.85% (-23.46 to 52.60) | 35.71% (-1.05 to 97.02) | 17.64% (-23.64 to 76.51) |
| Ovarian cancer | 3.16 (2.12 to 4.81) | 3.94 (2.75 to 5.59) | 3.97 (2.90 to 5.30) | 5.19 (3.53 to 7.42) | 24.49% (-18.59 to 87.37) | 0.96% (-31.36 to 47.09) | 30.66% (-14.94 to 110.19) |
| Testicular cancer | 3.73 (2.21 to 5.81) | 3.55 (2.08 to 5.67) | 3.54 (2.50 to 4.97) | 4.16 (2.71 to 6.14) | -4.66% (-41.38 to 60.84) | -0.48% (-31.45 to 57.61) | 17.75% (-32.58 to 88.88) |
| Kidney cancer | 4.54 (3.08 to 6.10) | 4.36 (2.90 to 5.86) | 4.10 (2.86 to 5.35) | 4.47 (3.12 to 5.89) | -4.00% (-28.35 to 28.98) | -5.89% (-29.38 to 29.01) | 9.03% (-21.52 to 52.88) |
| Bladder cancer | 2.13 (1.36 to 3.15) | 1.88 (1.28 to 2.45) | 1.46 (1.12 to 1.95) | 1.64 (1.13 to 2.26) | -11.78% (-38.71 to 20.02) | -22.43% (-45.46 to 11.26) | 12.81% (-23.56 to 70.27) |
| Brain and central nervous system cancer | 94.81 (66.13 to 125.85) | 90.05 (65.06 to 114.12) | 85.03 (62.94 to 106.74) | 89.53 (66.88 to 113.72) | -5.02% (-22.29 to 16.09) | -5.57% (-20.44 to 24.30) | 5.29% (-15.73 to 38.27) |
| Thyroid cancer | 2.46 (1.79 to 3.44) | 2.24 (1.67 to 3.10) | 2.35 (1.85 to 3.09) | 2.41 (1.82 to 3.24) | -9.01% (-35.08 to 38.69) | 4.94% (-26.88 to 49.75) | 2.52% (-30.25 to 43.92) |
| Mesothelioma | 0.41 (0.27 to 0.62) | 0.43 (0.29 to 0.58) | 0.48 (0.35 to 0.64) | 0.49 (0.32 to 0.67) | 4.81% (-32.42 to 63.53) | 11.11% (-25.35 to 58.43) | 3.18% (-32.64 to 57.08) |
| Hodgkin lymphoma | 21.33 (12.01 to 31.68) | 16.43 (9.51 to 24.28) | 12.92 (7.17 to 19.38) | 11.04 (5.80 to 16.46) | -22.96% (-47.55 to 16.92) | -21.36% (-42.75 to 15.78) | -14.53% (-45.05 to 33.16) |
| Non-Hodgkin lymphoma | 42.48 (31.36 to 55.39) | 38.56 (30.92 to 49.74) | 34.29 (27.22 to 42.28) | 37.14 (28.02 to 48.62) | -9.23% (-31.18 to 21.30) | -11.08% (-34.77 to 19.73) | 8.34% (-18.40 to 42.59) |
| Multiple myeloma | 0.64 (0.42 to 0.91) | 0.73 (0.50 to 1.02) | 0.90 (0.63 to 1.22) | 1.10 (0.77 to 1.47) | 13.79% (-18.26 to 53.46) | 23.11% (-9.07 to 79.97) | 21.66% (-17.81 to 82.72) |
| Leukemia | 240.82 (185.50 to 310.12) | 212.62 (164.24 to 262.13) | 178.25 (143.30 to 214.18) | 165.05 (132.39 to 201.42) | -11.71% (-29.74 to 8.75) | -16.16% (-28.61 to -1.49) | -7.41% (-28.15 to 17.12) |
| Other malignant neoplasms | 64.52 (48.87 to 85.17) | 55.53 (42.93 to 69.58) | 45.18 (35.72 to 57.99) | 40.32 (30.41 to 51.43) | -13.94% (-36.52 to 21.24) | -18.63% (-38.34 to 8.58) | -10.77% (-38.25 to 22.83) |
| Other neoplasms | 9.21 (5.29 to 14.10) | 8.53 (5.11 to 12.98) | 6.98 (4.42 to 10.47) | 7.63 (4.84 to 11.49) | -7.38% (-32.89 to 26.74) | -18.23% (-37.41 to 8.11) | 9.33% (-28.33 to 54.41) |
| Cardiovascular diseases | 1357.22 (1048.08 to 1661.94) | 1086.40 (877.56 to 1302.11) | 895.19 (738.40 to 1092.08) | 802.37 (654.88 to 983.16) | -19.95% (-34.43 to 0.19) | -17.60% (-33.10 to 1.50) | -10.37% (-31.97 to 19.25) |
| Rheumatic heart disease | 205.29 (128.09 to 280.92) | 135.60 (96.68 to 188.95) | 91.85 (64.47 to 128.55) | 77.55 (54.30 to 111.93) | -33.95% (-55.28 to -5.31) | -32.26% (-52.26 to -6.61) | -15.57% (-45.08 to 26.74) |
| Ischemic heart disease | 347.29 (243.75 to 478.89) | 297.22 (220.13 to 386.51) | 265.94 (202.71 to 347.10) | 253.28 (191.57 to 333.32) | -14.42% (-36.50 to 23.74) | -10.53% (-33.54 to 27.17) | -4.76% (-32.88 to 33.14) |
| Stroke | 545.27 (401.38 to 719.98) | 423.99 (323.91 to 543.93) | 339.65 (258.52 to 431.81) | 274.16 (209.27 to 359.13) | -22.24% (-40.20 to 4.13) | -19.89% (-38.26 to 1.04) | -19.28% (-44.50 to 14.17) |
| Hypertensive heart disease | 30.10 (17.06 to 47.57) | 26.28 (15.86 to 39.77) | 25.41 (17.28 to 37.96) | 24.47 (16.57 to 35.53) | -12.70% (-44.73 to 30.81) | -3.31% (-37.11 to 47.52) | -3.70% (-40.94 to 41.87) |
| Cardiomyopathy and myocarditis | 54.16 (30.42 to 88.15) | 46.39 (27.98 to 73.53) | 39.63 (25.63 to 60.42) | 37.02 (23.68 to 60.57) | -14.33% (-45.10 to 35.31) | -14.58% (-42.48 to 31.61) | -6.58% (-43.78 to 51.03) |
| Atrial fibrillation and flutter | 0.00 (0.00 to 0.00) | 0.00 (0.00 to 0.00) | 0.00 (0.00 to 0.00) | 0.00 (0.00 to 0.00) | --- | --- | --- |
| Aortic aneurysm | 1.37 (0.81 to 2.15) | 1.48 (0.97 to 2.29) | 2.04 (1.42 to 3.08) | 2.38 (1.58 to 3.46) | 8.17% (-29.16 to 72.63) | 38.30% (-10.07 to 103.78) | 16.57% (-33.01 to 105.54) |
| Lower extremity peripheral arterial disease | 0.00 (0.00 to 0.00) | 0.00 (0.00 to 0.00) | 0.00 (0.00 to 0.00) | 0.00 (0.00 to 0.00) | --- | --- | --- |
| Endocarditis | 11.35 (7.25 to 18.17) | 8.96 (5.68 to 13.51) | 7.72 (4.91 to 11.73) | 7.19 (4.91 to 11.00) | -21.08% (-51.70 to 24.78) | -13.76% (-45.07 to 42.42) | -6.85% (-46.40 to 64.40) |
| Non-rheumatic valvular heart disease | 15.01 (8.16 to 23.57) | 14.88 (8.80 to 23.65) | 14.43 (9.56 to 22.32) | 14.61 (8.88 to 22.21) | -0.88% (-39.48 to 57.55) | -3.05% (-39.49 to 45.45) | 1.27% (-36.81 to 68.29) |
| Other cardiovascular and circulatory diseases | 133.53 (82.91 to 197.07) | 121.90 (79.89 to 168.65) | 101.05 (71.16 to 136.07) | 105.70 (77.28 to 138.81) | -8.70% (-32.22 to 31.70) | -17.11% (-41.18 to 11.70) | 4.60% (-26.60 to 52.60) |
| Chronic respiratory diseases | 324.32 (230.91 to 439.20) | 306.91 (223.20 to 418.01) | 300.61 (221.24 to 411.36) | 298.33 (212.89 to 414.89) | -5.37% (-14.63 to 4.15) | -2.05% (-10.86 to 5.96) | -0.76% (-10.16 to 9.39) |
| Chronic obstructive pulmonary disease | 43.60 (28.87 to 61.65) | 41.50 (30.99 to 54.61) | 40.20 (32.50 to 52.39) | 35.71 (28.00 to 44.75) | -4.80% (-28.49 to 23.10) | -3.13% (-25.42 to 22.86) | -11.18% (-34.18 to 17.22) |
| Pneumoconiosis | 0.71 (0.33 to 1.34) | 0.66 (0.34 to 1.10) | 0.72 (0.41 to 1.19) | 0.72 (0.42 to 1.29) | -6.61% (-52.38 to 67.92) | 8.11% (-35.71 to 84.55) | 0.55% (-50.08 to 84.18) |
| Asthma | 257.25 (172.84 to 374.00) | 242.64 (163.63 to 351.96) | 236.73 (156.52 to 343.09) | 238.48 (153.84 to 355.09) | -5.68% (-13.37 to 2.24) | -2.44% (-9.38 to 4.90) | 0.74% (-6.80 to 7.76) |
| Interstitial lung disease and pulmonary sarcoidosis | 3.12 (1.51 to 6.23) | 3.26 (1.90 to 5.84) | 3.77 (2.37 to 5.88) | 3.82 (2.34 to 6.16) | 4.50% (-39.50 to 73.50) | 15.53% (-30.75 to 89.94) | 1.30% (-47.56 to 63.35) |
| Other chronic respiratory diseases | 19.63 (12.23 to 31.74) | 18.84 (12.77 to 27.62) | 19.19 (13.88 to 26.44) | 19.61 (14.60 to 26.02) | -4.05% (-34.61 to 29.96) | 1.87% (-25.93 to 36.60) | 2.16% (-29.73 to 46.27) |
| Cirrhosis and other chronic liver diseases | 158.65 (119.48 to 200.21) | 117.42 (91.70 to 149.99) | 91.54 (71.30 to 117.91) | 62.43 (48.41 to 77.16) | -25.99% (-42.32 to -8.71) | -22.04% (-39.02 to 0.42) | -31.80% (-50.51 to -3.70) |
| Digestive diseases | 308.40 (248.29 to 385.99) | 250.42 (201.01 to 313.35) | 211.25 (166.37 to 267.86) | 172.44 (137.78 to 215.05) | -18.80% (-30.86 to -5.71) | -15.64% (-27.49 to -0.82) | -18.37% (-32.39 to -2.74) |
| Appendicitis | 13.15 (7.32 to 20.21) | 10.49 (6.37 to 16.60) | 7.63 (5.16 to 10.69) | 6.68 (4.50 to 9.48) | -20.19% (-46.44 to 15.10) | -27.25% (-48.97 to 1.28) | -12.54% (-37.01 to 20.00) |
| Paralytic ileus and intestinal obstruction | 11.57 (7.30 to 17.13) | 8.66 (5.54 to 13.80) | 6.59 (4.46 to 10.24) | 6.35 (4.34 to 9.85) | -25.16% (-53.33 to 19.16) | -23.85% (-52.28 to 20.92) | -3.61% (-41.48 to 59.78) |
| Inguinal, femoral, and abdominal hernia | 13.59 (7.77 to 23.78) | 13.55 (7.50 to 23.21) | 12.58 (7.21 to 21.07) | 11.49 (6.38 to 18.86) | -0.33% (-12.07 to 14.41) | -7.12% (-17.21 to 4.52) | -8.69% (-19.45 to 7.72) |
| Inflammatory bowel disease | 5.15 (3.37 to 7.85) | 4.79 (3.26 to 6.84) | 4.22 (3.02 to 6.00) | 4.30 (2.98 to 6.00) | -6.85% (-33.24 to 34.43) | -11.86% (-37.21 to 22.81) | 1.75% (-28.55 to 49.43) |
| Vascular intestinal disorders | 1.20 (0.71 to 1.88) | 1.06 (0.68 to 1.67) | 0.85 (0.56 to 1.26) | 0.92 (0.59 to 1.42) | -11.73% (-46.27 to 48.81) | -19.94% (-50.21 to 25.08) | 8.01% (-30.41 to 84.86) |
| Gallbladder and biliary diseases | 17.63 (11.00 to 26.10) | 16.96 (10.60 to 25.64) | 16.70 (10.16 to 25.33) | 16.58 (10.16 to 24.90) | -3.80% (-13.42 to 6.83) | -1.54% (-12.58 to 8.62) | -0.69% (-10.79 to 10.01) |
| Pancreatitis | 4.50 (2.75 to 7.01) | 3.97 (2.51 to 5.97) | 3.51 (2.29 to 5.02) | 3.27 (2.23 to 4.67) | -11.84% (-35.90 to 17.11) | -11.53% (-35.52 to 22.10) | -6.94% (-32.01 to 36.04) |
| Neurological disorders | 897.85 (636.97 to 1223.37) | 876.60 (625.72 to 1201.42) | 890.27 (633.05 to 1199.31) | 890.91 (627.55 to 1213.95) | -2.37% (-8.91 to 5.36) | 1.56% (-4.39 to 8.61) | 0.07% (-5.62 to 5.91) |
| Alzheimer's disease and other dementias | 0.00 (0.00 to 0.00) | 0.00 (0.00 to 0.00) | 0.00 (0.00 to 0.00) | 0.00 (0.00 to 0.00) | --- | --- | --- |
| Parkinson's disease | 0.05 (0.04 to 0.06) | 0.05 (0.04 to 0.07) | 0.06 (0.04 to 0.08) | 0.06 (0.05 to 0.09) | 4.84% (-17.46 to 26.87) | 16.40% (-3.21 to 40.79) | 11.02% (-10.20 to 39.28) |
| Idiopathic epilepsy | 226.64 (160.65 to 316.99) | 200.60 (140.45 to 266.75) | 180.74 (129.12 to 256.70) | 166.49 (111.40 to 244.74) | -11.49% (-29.54 to 13.64) | -9.90% (-27.24 to 16.24) | -7.88% (-28.27 to 19.24) |
| Multiple sclerosis | 3.72 (2.47 to 5.70) | 3.97 (2.82 to 5.56) | 4.42 (3.06 to 6.05) | 4.47 (3.16 to 6.47) | 6.68% (-20.45 to 44.22) | 11.24% (-15.63 to 50.24) | 1.18% (-27.72 to 44.71) |
| Motor neuron disease | 1.36 (0.88 to 2.35) | 1.57 (1.02 to 2.56) | 2.21 (1.47 to 3.51) | 3.61 (2.30 to 6.39) | 15.17% (-18.18 to 65.29) | 40.58% (0.05 to 102.63) | 63.71% (-7.45 to 158.89) |
| Other neurological disorders | 15.48 (10.37 to 22.97) | 18.50 (12.78 to 25.94) | 27.72 (20.39 to 37.71) | 47.15 (35.48 to 63.49) | 19.50% (-13.82 to 60.92) | 49.83% (14.32 to 99.68) | 70.10% (28.49 to 122.48) |
| Mental disorders | 2382.55 (1598.89 to 3295.83) | 2400.99 (1687.71 to 3164.17) | 2338.52 (1602.79 to 3092.23) | 2916.30 (2013.43 to 4165.94) | 0.77% (-11.72 to 15.14) | -2.60% (-14.35 to 11.00) | 24.71% (7.06 to 60.60) |
| Schizophrenia | 82.48 (48.73 to 121.04) | 88.81 (54.06 to 130.80) | 95.95 (57.80 to 139.68) | 87.15 (52.44 to 130.34) | 7.67% (0.45 to 15.27) | 8.03% (1.13 to 15.32) | -9.17% (-14.88 to -2.99) |
| Alcohol use disorders | 31.72 (18.55 to 50.90) | 31.35 (17.95 to 50.16) | 31.74 (17.93 to 52.99) | 28.30 (16.30 to 46.97) | -1.16% (-12.04 to 8.50) | 1.25% (-10.49 to 11.87) | -10.84% (-18.73 to -1.81) |
| Drug use disorders | 137.97 (98.89 to 179.97) | 164.46 (119.46 to 214.79) | 188.51 (140.91 to 244.28) | 161.71 (118.60 to 207.86) | 19.20% (6.79 to 36.14) | 14.62% (3.35 to 29.61) | -14.22% (-26.39 to -0.47) |
| Depressive disorders | 652.71 (427.63 to 1006.04) | 694.80 (473.00 to 997.48) | 727.52 (492.28 to 1036.10) | 944.68 (614.61 to 1356.17) | 6.45% (-11.97 to 32.59) | 4.71% (-13.45 to 30.40) | 29.85% (3.56 to 60.48) |
| Bipolar disorder | 118.35 (63.62 to 181.40) | 122.87 (64.42 to 190.98) | 128.46 (68.04 to 201.61) | 121.49 (64.02 to 188.49) | 3.82% (-0.72 to 8.31) | 4.55% (0.22 to 8.53) | -5.43% (-9.18 to -1.39) |
| Anxiety disorders | 965.79 (545.43 to 1488.83) | 928.33 (544.15 to 1413.62) | 820.32 (512.31 to 1178.80) | 1171.07 (650.22 to 1951.61) | -3.88% (-28.88 to 31.53) | -11.63% (-37.26 to 12.72) | 42.76% (-7.97 to 135.36) |
| Eating disorders | 72.87 (41.19 to 113.90) | 78.59 (44.24 to 124.41) | 87.60 (48.74 to 141.18) | 84.36 (47.14 to 134.11) | 7.84% (2.62 to 14.01) | 11.47% (5.22 to 18.36) | -3.70% (-8.65 to 2.30) |
| Autism spectrum disorders | 120.36 (55.85 to 240.50) | 121.18 (62.85 to 212.95) | 126.89 (67.20 to 214.08) | 152.67 (62.59 to 330.43) | 0.68% (-15.89 to 40.91) | 4.71% (-35.36 to 63.68) | 20.31% (-31.37 to 143.80) |
| Attention-deficit/hyperactivity disorder | 115.64 (68.44 to 175.14) | 116.69 (70.63 to 176.53) | 112.78 (67.63 to 169.85) | 110.03 (65.19 to 166.64) | 0.91% (-3.31 to 5.85) | -3.36% (-7.28 to 1.80) | -2.43% (-6.00 to 0.79) |
| Conduct disorder | 177.75 (100.44 to 298.53) | 172.41 (97.86 to 288.34) | 159.97 (91.13 to 263.63) | 169.34 (96.10 to 281.70) | -3.00% (-5.52 to -0.29) | -7.22% (-9.90 to -4.29) | 5.85% (2.73 to 8.75) |
| Idiopathic developmental intellectual disability | 38.89 (16.04 to 79.06) | 37.99 (15.62 to 76.08) | 36.50 (14.99 to 73.47) | 35.56 (12.48 to 72.57) | -2.32% (-11.91 to 7.32) | -3.92% (-20.56 to 13.73) | -2.60% (-30.66 to 16.73) |
| Other mental disorders | 37.70 (20.54 to 58.94) | 39.31 (22.00 to 62.11) | 42.53 (23.74 to 66.83) | 39.95 (22.31 to 63.07) | 4.29% (-1.14 to 10.24) | 8.18% (1.91 to 14.29) | -6.06% (-11.35 to -0.41) |
| Diabetes mellitus | 81.61 (60.82 to 108.32) | 84.64 (62.45 to 113.10) | 104.25 (79.05 to 140.69) | 119.05 (89.53 to 162.46) | 3.72% (-8.49 to 19.27) | 23.16% (9.08 to 39.34) | 14.20% (0.16 to 25.18) |
| Acute glomerulonephritis | 1.16 (0.46 to 2.22) | 0.74 (0.32 to 1.44) | 0.77 (0.40 to 1.29) | 0.73 (0.42 to 1.15) | -36.20% (-66.97 to 12.35) | 4.25% (-38.32 to 118.97) | -5.15% (-52.27 to 77.47) |
| Chronic kidney disease | 146.55 (106.50 to 199.60) | 122.84 (93.69 to 162.93) | 115.62 (94.56 to 148.74) | 131.15 (100.91 to 162.09) | -16.18% (-32.66 to 10.67) | -5.87% (-27.77 to 17.89) | 13.43% (-19.82 to 51.22) |
| Urinary diseases and male infertility | 18.52 (14.17 to 25.47) | 16.19 (12.16 to 22.87) | 16.36 (12.13 to 24.09) | 15.72 (11.66 to 23.14) | -12.60% (-23.54 to -4.06) | 1.04% (-10.38 to 13.81) | -3.88% (-15.74 to 8.83) |
| Gynecological diseases | 199.12 (131.21 to 301.84) | 203.80 (135.11 to 310.97) | 215.27 (143.58 to 328.40) | 202.26 (134.15 to 308.47) | 2.35% (-0.58 to 5.14) | 5.63% (3.05 to 8.64) | -6.04% (-8.51 to -3.34) |
| Hemoglobinopathies and hemolytic anemias | 298.41 (216.36 to 422.39) | 248.99 (179.08 to 339.11) | 209.54 (153.96 to 278.00) | 167.03 (120.85 to 219.07) | -16.56% (-29.59 to 2.20) | -15.85% (-26.51 to -0.75) | -20.29% (-33.32 to -6.24) |
| Endocrine, metabolic, blood, and immune disorders | 82.05 (56.98 to 125.39) | 78.94 (56.45 to 113.91) | 78.38 (55.20 to 108.96) | 87.46 (63.46 to 123.96) | -3.78% (-20.35 to 17.03) | -0.71% (-15.91 to 19.17) | 11.59% (-7.96 to 39.80) |
| Musculoskeletal disorders | 777.98 (527.68 to 1066.52) | 803.24 (537.89 to 1097.79) | 837.00 (566.66 to 1135.01) | 798.07 (541.39 to 1088.26) | 3.25% (0.93 to 5.60) | 4.20% (1.75 to 6.94) | -4.65% (-7.14 to -1.98) |
| Rheumatoid arthritis | 3.92 (2.37 to 5.63) | 4.06 (2.49 to 6.05) | 4.55 (2.78 to 6.59) | 5.04 (3.08 to 7.41) | 3.53% (-13.70 to 20.64) | 12.02% (-2.07 to 26.27) | 10.85% (-6.50 to 25.94) |
| Osteoarthritis | 0.00 (0.00 to 0.00) | 0.00 (0.00 to 0.00) | 0.00 (0.00 to 0.00) | 0.00 (0.00 to 0.00) | --- | --- | --- |
| Low back pain | 588.15 (401.00 to 823.28) | 605.29 (406.50 to 859.72) | 614.94 (407.42 to 860.29) | 578.98 (393.97 to 824.97) | 2.92% (0.02 to 5.90) | 1.59% (-1.64 to 4.48) | -5.85% (-8.76 to -2.65) |
| Neck pain | 124.97 (67.13 to 207.54) | 124.95 (66.42 to 206.59) | 129.28 (68.56 to 212.87) | 123.81 (66.21 to 205.36) | -0.01% (-3.15 to 3.24) | 3.46% (-0.37 to 7.21) | -4.23% (-7.52 to -1.17) |
| Gout | 0.29 (0.13 to 0.52) | 0.31 (0.15 to 0.57) | 0.37 (0.18 to 0.65) | 0.34 (0.16 to 0.61) | 9.31% (6.30 to 14.08) | 16.40% (12.70 to 20.74) | -6.79% (-10.29 to -4.03) |
| Other musculoskeletal disorders | 60.66 (38.46 to 88.15) | 68.62 (42.92 to 100.80) | 87.87 (55.47 to 128.28) | 89.90 (56.11 to 127.35) | 13.12% (0.81 to 26.65) | 28.05% (16.25 to 41.83) | 2.31% (-7.00 to 13.47) |
| Other non-communicable diseases | 1155.28 (922.03 to 1436.71) | 1056.64 (851.68 to 1318.45) | 989.36 (791.74 to 1257.93) | 907.53 (722.29 to 1128.77) | -8.54% (-18.29 to 0.08) | -6.37% (-13.56 to 1.10) | -8.27% (-17.80 to -0.20) |
| Congenital birth defects | 464.72 (353.64 to 585.63) | 414.90 (339.65 to 490.73) | 374.90 (311.68 to 455.79) | 344.01 (277.99 to 415.83) | -10.72% (-30.32 to 7.93) | -9.64% (-25.09 to 7.17) | -8.24% (-26.47 to 12.39) |
| Skin and subcutaneous diseases | 620.42 (374.57 to 941.29) | 615.48 (377.00 to 937.05) | 608.76 (373.82 to 924.28) | 632.62 (389.80 to 965.49) | -0.80% (-2.54 to 1.37) | -1.09% (-2.38 to 0.41) | 3.92% (2.38 to 5.81) |
| Dermatitis | 408.08 (218.27 to 701.50) | 402.10 (215.77 to 688.03) | 393.22 (213.22 to 671.07) | 403.23 (217.81 to 684.44) | -1.47% (-2.94 to -0.01) | -2.21% (-3.64 to -0.72) | 2.54% (0.86 to 3.82) |
| Psoriasis | 14.13 (9.69 to 18.74) | 15.84 (11.31 to 21.21) | 17.28 (11.97 to 23.29) | 18.67 (13.18 to 25.04) | 12.09% (1.25 to 23.86) | 9.10% (-1.49 to 20.52) | 8.02% (-2.30 to 18.26) |
| Scabies | 10.04 (5.65 to 16.77) | 9.98 (5.67 to 16.63) | 10.00 (5.68 to 16.64) | 9.84 (5.64 to 16.55) | -0.60% (-4.81 to 3.45) | 0.21% (-3.87 to 5.28) | -1.61% (-5.68 to 2.10) |
| Fungal skin diseases | 44.11 (16.67 to 101.70) | 34.98 (13.37 to 79.27) | 29.26 (11.09 to 66.23) | 24.93 (9.41 to 56.14) | -20.70% (-25.80 to -15.60) | -16.33% (-19.35 to -12.23) | -14.82% (-19.57 to -10.11) |
| Viral skin diseases | 38.11 (25.06 to 58.00) | 37.63 (24.63 to 56.79) | 36.75 (24.13 to 56.32) | 37.84 (24.85 to 57.11) | -1.26% (-3.81 to 1.54) | -2.32% (-5.09 to 0.31) | 2.95% (-0.15 to 6.38) |
| Acne vulgaris | 32.28 (19.47 to 54.87) | 41.93 (25.09 to 70.10) | 49.78 (30.18 to 83.49) | 64.80 (39.02 to 106.94) | 29.87% (22.81 to 37.44) | 18.74% (13.08 to 25.27) | 30.17% (23.59 to 38.18) |
| Alopecia areata | 0.93 (0.59 to 1.30) | 0.95 (0.62 to 1.35) | 0.97 (0.61 to 1.37) | 0.96 (0.60 to 1.39) | 2.42% (-12.13 to 15.95) | 2.29% (-10.35 to 16.22) | -1.77% (-14.27 to 14.12) |
| Pruritus | 0.82 (0.36 to 1.63) | 0.84 (0.36 to 1.69) | 0.90 (0.40 to 1.79) | 0.89 (0.39 to 1.79) | 2.74% (-5.46 to 10.66) | 6.50% (-1.29 to 14.72) | -1.07% (-9.45 to 7.58) |
| Urticaria | 58.08 (36.08 to 86.04) | 57.63 (36.02 to 86.68) | 56.53 (35.31 to 83.11) | 57.48 (36.21 to 85.41) | -0.77% (-4.80 to 2.69) | -1.91% (-5.73 to 1.93) | 1.68% (-2.68 to 6.17) |
| Decubitus ulcer | 2.12 (1.10 to 3.40) | 1.83 (0.97 to 3.11) | 1.85 (1.15 to 2.82) | 1.66 (0.95 to 2.69) | -13.84% (-45.30 to 46.54) | 1.38% (-38.40 to 62.22) | -10.30% (-51.57 to 65.48) |
| Other skin and subcutaneous diseases | 9.86 (5.13 to 17.38) | 9.84 (5.31 to 17.69) | 10.15 (5.24 to 18.85) | 10.21 (5.37 to 18.57) | -0.16% (-6.59 to 5.55) | 3.10% (-2.90 to 8.61) | 0.57% (-4.76 to 7.42) |
| Sense organ diseases | 261.12 (173.72 to 359.17) | 255.73 (170.85 to 354.69) | 255.00 (168.83 to 353.96) | 237.57 (157.04 to 329.29) | -2.06% (-4.20 to 0.03) | -0.28% (-2.76 to 1.77) | -6.84% (-9.29 to -4.82) |
| Age-related and other hearing loss | 183.35 (112.21 to 265.25) | 181.07 (110.14 to 259.07) | 182.51 (111.70 to 259.41) | 174.31 (104.81 to 252.13) | -1.24% (-4.26 to 1.53) | 0.80% (-1.94 to 3.88) | -4.50% (-7.30 to -1.83) |
| Other sense organ diseases | 8.89 (4.90 to 14.40) | 9.03 (4.82 to 14.62) | 9.11 (4.98 to 14.82) | 9.10 (4.96 to 14.52) | 1.53% (-4.93 to 8.97) | 0.95% (-5.81 to 8.90) | -0.11% (-6.80 to 6.68) |
| Oral disorders | 92.45 (52.38 to 151.07) | 93.82 (53.38 to 152.14) | 94.91 (53.95 to 155.69) | 91.04 (51.55 to 151.58) | 1.48% (-4.54 to 8.89) | 1.16% (-0.68 to 3.66) | -4.08% (-6.01 to -1.82) |
| Headache disorders | 650.59 (430.76 to 930.91) | 651.91 (443.04 to 920.13) | 675.13 (451.72 to 949.97) | 669.12 (446.20 to 953.91) | 0.20% (-2.16 to 2.86) | 3.56% (0.80 to 6.36) | -0.89% (-3.20 to 1.57) |
| Substance use disorders | 169.69 (120.71 to 221.29) | 195.82 (142.25 to 251.31) | 220.25 (163.90 to 287.05) | 190.01 (142.53 to 239.66) | 15.39% (4.68 to 29.50) | 12.48% (3.18 to 25.35) | -13.73% (-24.84 to -1.92) |
| Diabetes and kidney diseases | 229.31 (181.59 to 285.62) | 208.22 (168.05 to 262.23) | 220.64 (180.21 to 264.99) | 250.93 (202.38 to 301.37) | -9.20% (-21.33 to 6.64) | 5.97% (-10.07 to 22.49) | 13.73% (-5.26 to 35.34) |
| Bacterial skin diseases | 1.87 (1.00 to 3.23) | 1.93 (1.10 to 3.23) | 2.05 (1.16 to 3.47) | 2.13 (1.30 to 3.62) | 3.38% (-27.36 to 53.43) | 6.28% (-17.16 to 35.26) | 3.79% (-25.25 to 33.66) |
| Blindness and vision loss | 68.88 (47.87 to 99.32) | 65.63 (45.22 to 95.02) | 63.38 (42.72 to 91.63) | 54.16 (36.95 to 79.77) | -4.71% (-8.78 to -0.87) | -3.44% (-7.54 to 0.45) | -14.54% (-18.27 to -10.46) |
| Upper digestive system diseases | 75.79 (50.82 to 113.04) | 67.72 (43.86 to 105.16) | 62.69 (37.74 to 101.76) | 54.62 (32.03 to 88.98) | -10.64% (-26.65 to 2.84) | -7.43% (-19.38 to 4.02) | -12.88% (-21.24 to -5.89) |
| Pulmonary Arterial Hypertension | 13.87 (6.83 to 24.52) | 9.69 (5.01 to 17.00) | 7.46 (4.31 to 12.12) | 6.00 (3.32 to 10.28) | -30.09% (-59.20 to 34.49) | -23.03% (-56.27 to 36.77) | -19.58% (-62.24 to 50.27) |
| Eye cancer | 0.18 (0.11 to 0.30) | 0.16 (0.10 to 0.25) | 0.16 (0.10 to 0.27) | 0.17 (0.11 to 0.28) | -13.63% (-43.19 to 24.73) | 0.50% (-38.94 to 48.89) | 6.71% (-31.53 to 59.99) |
| Soft tissue and other extraosseous sarcomas | 11.91 (7.38 to 19.82) | 10.37 (6.73 to 15.20) | 8.43 (5.80 to 12.23) | 8.92 (5.75 to 13.59) | -12.93% (-45.92 to 39.45) | -18.69% (-48.70 to 27.12) | 5.87% (-32.27 to 60.69) |
| Malignant neoplasm of bone and articular cartilage | 60.27 (39.21 to 98.52) | 56.11 (39.47 to 82.82) | 50.43 (35.73 to 67.11) | 48.57 (35.82 to 69.30) | -6.90% (-40.80 to 39.95) | -10.12% (-35.24 to 30.36) | -3.70% (-38.45 to 45.42) |
| Neuroblastoma and other peripheral nervous cell tumors | 1.47 (0.99 to 2.23) | 1.47 (0.97 to 2.15) | 1.41 (0.95 to 1.94) | 1.79 (1.22 to 2.66) | 0.03% (-29.21 to 39.59) | -3.83% (-27.36 to 39.21) | 26.76% (-19.90 to 92.99) |
| Other digestive diseases | 7.17 (4.69 to 10.16) | 5.80 (4.15 to 8.14) | 4.92 (3.54 to 6.67) | 5.80 (4.15 to 8.31) | -19.12% (-44.06 to 16.55) | -15.19% (-37.78 to 12.34) | 18.02% (-21.24 to 81.39) |
